# Monoclonal Anti-AMP Antibodies Are Sensitive and Valuable Tools for Detecting Patterns of AMPylation

**DOI:** 10.1016/j.isci.2020.101800

**Published:** 2020-11-13

**Authors:** Dorothea Höpfner, Joel Fauser, Marietta S. Kaspers, Christian Pett, Christian Hedberg, Aymelt Itzen

**Affiliations:** 1 Department of Biochemistry and Signaltransduction, University Medical Center Hamburg-Eppendorf (UKE), Martinistr. 52, 20246 Hamburg, Germany; 2 Center for Integrated Protein Science Munich (CIPSM), Department Chemistry, Technical University of Munich, Lichtenbergstrasse 4, 85747 Garching, Germany; 3 Chemical Biology Center (KBC), Department of Chemistry, Umeå University, Linnaeus väg 10, 90187 Umeå, Sweden; 4 Center for Structural Systems Biology (CSSB), University Medical Center Hamburg-Eppendorf (UKE), Notkestraße 85, Building 15, 22607 Hamburg, Germany

**Keywords:** Biochemistry, Biomolecules, Molecular Biology, Biotechnology

## Abstract

AMPylation is a post-translational modification that modifies amino acid side chains with adenosine monophosphate (AMP). Recently, a role of AMPylation as a universal regulatory mechanism in infection and cellular homeostasis has emerged, driving the demand for universal tools to study this modification. Here, we describe three monoclonal anti-AMP antibodies (mAbs) from mouse that are capable of protein backbone-independent recognition of AMPylation, in denatured (western blot) as well as native (ELISA, IP) applications, thereby outperforming previously reported tools. These antibodies are highly sensitive and specific for AMP modifications, highlighting their potential as tools for new target identification, as well as for validation of known targets. Interestingly, applying the anti-AMP mAbs to various cancer cell lines reveals a previously undescribed broad and diverse AMPylation pattern. In conclusion, these anti-AMP mABs will further advance the current understanding of AMPylation and the spectrum of modified targets.

## Highlights

• Generation of murine monoclonal anti-AMP antibodies via synthetic AMPylated peptide
• Characterization in the applications western blot, ELISA, and immunoprecipitation
• Sensitive and specific recognition of AMPylation independent of protein backbone
• Expansion of toolbox for the detection and enrichment of AMPylation

## Graphical Abstract

**Figure:**
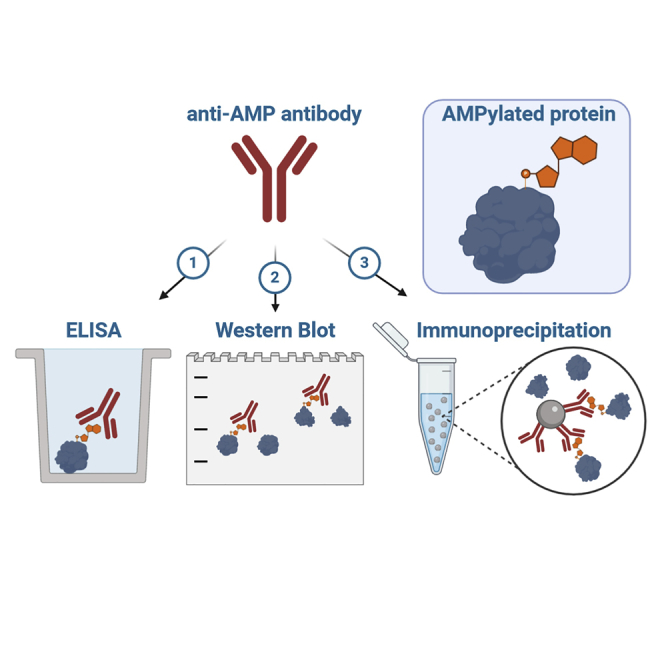

## Introduction

Post-translational modifications (PTMs) are diverse covalent alterations that modulate the activity, localization, stability, and specificity of proteins. One such PTM is AMPylation (also referred to as adenylylation) that occurs in both prokaryotes and eukaryotes. Enzymes utilize adenosine triphosphate (ATP) as donor substrate to transfer the adenosine monophosphate (AMP) to hydroxyl-bearing amino acid side chains (e.g., tyrosine, serine, threonine) of a target protein, with pyrophosphate being released as a side product. There are three different classes of AMPylators or protein adenylyl transferases known to date: DNA-β-Polymerase-like AMPylators with their most prominent member being glutamine synthetase adenylyltransferase from *Escherichia coli* (Kingdon et al., 1967); FIC (*Filamentation induced by cyclic AMP*) enzymes as represented by human FICD (Engel et al., 2012), IbpA from *Histophilus somni* (Worby et al., 2009), or VopS from *Vibrio parahaemolyticus* (Yarbrough et al., 2009); and, the most recently discovered, pseudokinases, specifically the highly conserved SelO (Sreelatha et al., 2018).

AMPylation has been studied over 50 years (Kingdon et al., 1967) and has gained recent attention with the identification of small GTPases as targets of AMPylating enzymes during various bacterial infections (Worby et al., 2009; Yarbrough et al., 2009). The discovery of FICD as the only metazoan FIC protein (Engel et al., 2012; Worby et al., 2009) and its modification of the endoplasmic reticulum (ER) chaperone BiP (Ham et al., 2014; Sanyal et al., 2015) illustrates a role of AMPylation in protein homeostasis (Preissler et al, 2015, 2016). Recent findings on AMPylation by pseudokinases (Sreelatha et al., 2018) hints at a broader occurrence of this modification as a general mechanism, and not just in the context of bacterial infections as previously thought.

However, despite a high prevalence of predicted FIC enzymes based on their conserved sequence, especially in pathogenic bacteria (Khater and Mohanty, 2015), only a limited amount of AMPylation targets are known. This discrepancy between the number of enzymes and identified targets highlights the challenge of detecting AMPylation. Available tools are both limited and are associated with disadvantages in terms of necessary resources and/or studying AMPylation in a physiologically relevant context. ATP analogs have reduced intracellular uptake (Plagemann and Wohlhueter, 1980) (although recent work established a cell-permeable pronucleotide probe, Kielkowski et al., 2020), are outcompeted by the high endogenous concentrations of ATP, and are hampered by the potential inability of enzymes to use these analogs as substrates. When used in cell lysates, spatial and temporal regulation is abrogated.

Antibodies targeting AMPylation could overcome many of these challenges as well as offer further applications as an orthogonal approach. Ideally, such an antibody would be able to detect AMPylation with high sensitivity and specificity toward both native and denatured proteins, thus enabling western blot (WB) detection as well as enrichment by immunoprecipitation (IP). Previously generated polyclonal antibodies using AMPylated peptides do not fulfill these desired properties (Hao et al., 2011; Smit et al., 2011).

Here, we generate three new monoclonal antibodies from mice that recognize AMPylation independently of the protein backbone, under denatured as well as native conditions. In addition to target validation, they can serve as new tools for target identification. As new target identification hinges on proper positive controls, and false-negatives may not be detected, a thorough characterization of the antibodies' behavior in the specific application is crucial.

## Results

**Figure 1:**
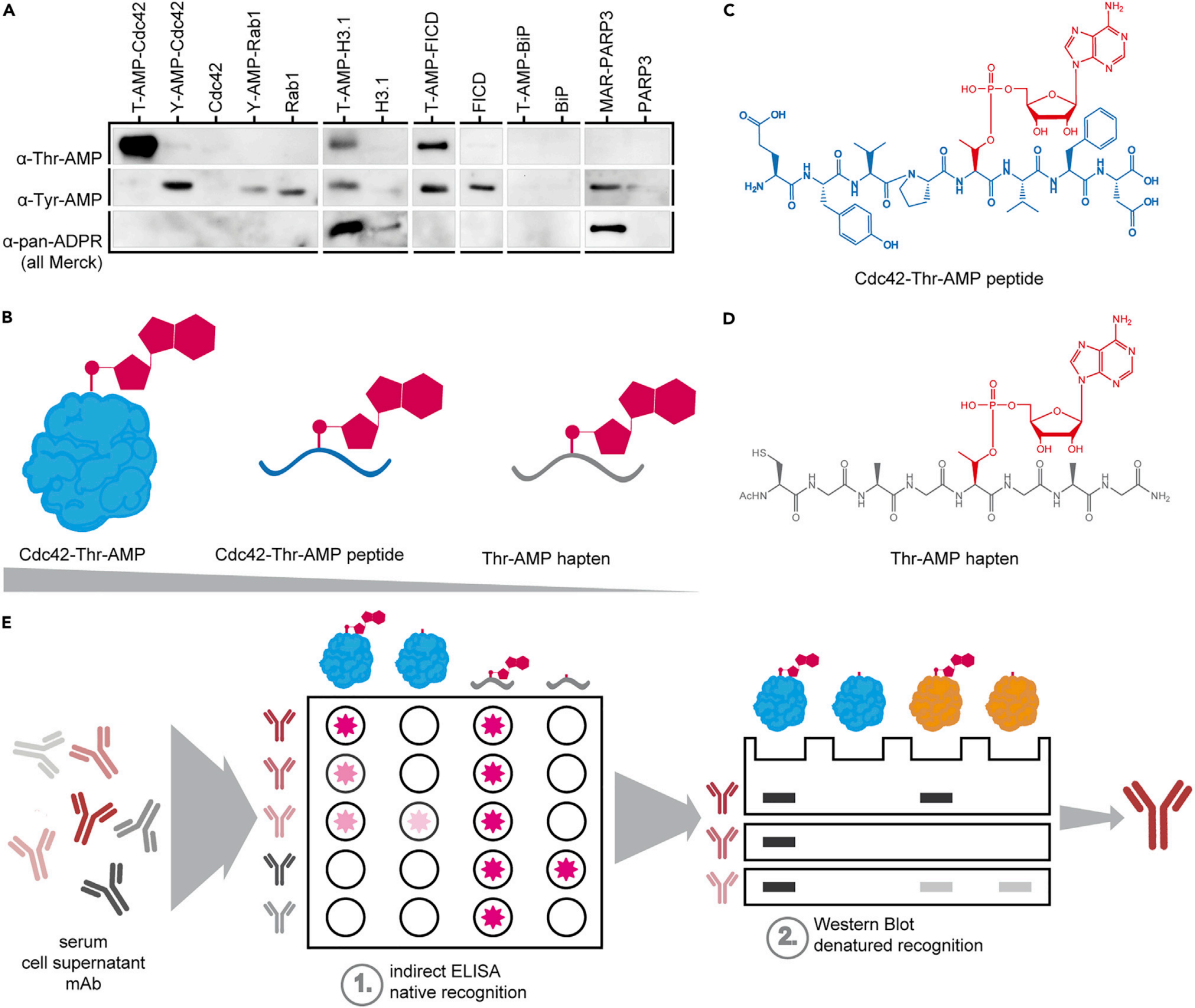
Motivation, Hapten Design, and Selection Strategy for the Generation of Monoclonal Anti-AMP Antibodies (A) Performance of commercially available anti-AMP antibodies on AMPylated proteins. 50 ng of indicated recombinant protein was analyzed by WB using anti-threonine-AMP (Merck) and anti-tyrosine-AMP (Merck) antibodies as well as anti-ADPR binding probe (Merck) as indicated. Rows represent identical WBs. (B) Reductive approach of hapten design. Instead of using intact AMPylated protein or AMPylated peptide (blue) from a naturally occurring target such as Cdc42, the peptide backbone's complexity was reduced (gray) to ensure development of antibodies against the AMP-moiety (red) alone. (C) Representation of the peptide 31-38aa in naturally occurring Cdc42-Thr-AMP with its complex side chains (blue). The AMP-modified threonine is indicated in red. (D) See also Figures S1 and S2. Representation of Thr-AMP hapten peptide with its reduced complexity of a glycine-alanine backbone, N-terminal acetylation, and C-terminal amidation. An N-terminal cysteine was included to enable fusion to carrier protein. The AMP-modified threonine is indicated in red. (E) See also Figure S3. Illustration of the stepwise selection process of mice (sera), clones (supernatant), and subclones (supernatant) and confirmation of purified antibodies during antibody generation. Candidates were first subjected to ELISA against the AMPylated hapten peptide as well as AMPylated Cdc42-Thr and counterscreened against their unmodified counterparts; positive clones evaluated for their performance in WB on various AMPylated proteins and their unmodified counterparts.

Previously published and commercially available antibodies claimed to recognize AMPylated threonine and tyrosine, respectively, independent of the peptide backbone and protein environment (see Sigma-Aldrich 09-890 and ABS184) (Hao et al., 2011). However, evaluation of their performance in WB on various recombinant proteins with different modified amino acid side chains (such as Rho GTPase Cdc42 AMPylated at threonine35 [VopS, Yarbrough et al., 2009] or tyrosine32 [IbpA, Worby et al., 2009], Rab GTPase Rab1b modified at tyrosine77 (DrrA from *Legionella pneumophila,* Müller et al., 2010), Histone H3.1 modified at threonine (FICD, Truttmann et al., 2016), the ER chaperone BiP/Grp-78 modified at threonine518 (FICD, Preissler et al., 2015), and the FIC enzyme FICD auto-modified at threonine80,183 + serine79 (Sanyal et al., 2015) showed that they do not recognize all AMPylations (Figure 1A): the commercially available anti-Thr-AMP antibody (Sigma-Aldrich 09-890) successfully recognized Cdc42-Thr-AMP and FICD-Thr-AMP, whereas the detection of H3.1-Thr-AMP and BiP-Thr-AMP was less sensitive and in the case of the latter no longer possible at 50 ng. The commercially available anti-Tyr-AMP antibody (ABS184, Merck) showed unsatisfactory performance by cross-reacting with unmodified Rab1b and FICD, respectively, as well as H3.1-Thr-AMP, in addition to exhibiting a generally weak detection signal. As both anti-AMP antibodies did not yield broad recognition of AMPylation, we considered whether available anti-ADP-ribosylation antibodies might also be able to detect AMPylation, because both modifications share the AMP moiety. We therefore tested the commercially available anti-pan-ADP-ribose-binding reagent (MABE1016, Merck) and, while detecting mono-ADP-ribosylated PARP3 (by autocatalysis, Vyas et al., 2014), found it unable to detect AMPylation with the exception of H3.1-Thr-AMP.

This number of false-positive and false-negative signals in commercially available anti-Tyr-AMP and anti-ADPR antibodies as well as the low sensitivity in the anti-Thr-AMP antibody led us to the development of new monoclonal anti-AMP antibodies in mice.

### Design and Synthesis of the AMP-Bearing Peptide

Previously published antibodies worked mostly against denatured targets in WB and were only evaluated against small GTPases (dependent on the peptide used for immunization) (Hao et al., 2011; Smit et al., 2011). Our goal was to create a universal tool that can recognize AMPylation, not only on the rising number of known AMPylated proteins but also on unknown targets independent of backbone and protein environment, in denatured as well as native applications. This would allow for target enrichment from complex samples as well as target validation and characterization.

Instead of using an AMPylated peptide derived from a naturally occurring target protein as hapten, we chose a reductive approach that aimed to develop the antibody against the AMP side chain moiety alone, but not against the peptide sequence itself (Figure 1B). The strategy was therefore to reduce the peptide backbone (Figure 1C) to a non-immunogenic 8 amino acid sequence of glycine and alanine, long enough to not unintentionally cause an immune reaction to the termini, but short enough to diminish the immune response to the peptide itself. To lower the charge at the termini and simulate a natural protein peptide backbone, the peptide was N-terminally acetylated and C-terminally amidated. The AMPylated threonine was introduced in the middle of the synthesized peptide via the use of an AMPylated building block (Albers et al., 2011; Smit et al., 2011). An N-terminal cysteine was incorporated to enable fusion to carrier proteins for immunization (Figure 1D; for synthesis see Figures S1 and S2).

As this reductive strategy of incorporating AMPylated threonine into a short glycine-alanine backbone has never been tested before and posed the risk of abolishing immunogenicity, we decided on a broad approach, choosing two different carrier proteins as hapten conjugates and two different mice breeds for immunization. In total, 10 mice were immunized with either BSA or keyhole limpet hemocyanin (KLH) conjugates, each of which were injected into three BALB/c and two C57BL/6 mice by GenScript.

To ensure backbone-independent recognition of AMPylation, antibody candidates were reversely selected by a stepwise screening procedure during all stages of development (Figure 1E). The screening process started with all candidates that were able to recognize the hapten with its reduced backbone complexity, proceeding to filter all candidates that were capable of recognition of native threonine-AMPylated Cdc42 as determined by ELISA (Figure 1E, first step), and subsequently testing recognition of multiple modified proteins in denatured state via WB (Figure 1E, second step). Only candidates positive for all these criteria and all target proteins were taken into consideration and used for further development (see Figure S3).

Our selection strategy, followed by rigorous characterization, aimed to overcome the aforementioned pitfalls of currently available antibodies and created three new antibodies against AMPylation: One promising clone, 17G6, with sensitive recognition of all AMPylated proteins in WB independent of their modified side chain, native recognition of Cdc42-Thr-AMP in ELISA, and low background was selected for subcloning and subsequent production and purification. Another candidate, 7C11, was selected for showing a bias in WB for threonine-modified protein. One further clone, 1G11, was selected due to its development of a Tyr-AMP-specific recognition, despite immunization with a threonine-modified peptide. All monoclonal antibodies were derived from C57 BL/6 mice. 17G6's immunogen was a BSA-fusion, whereas 7C11's and 1G11's immunogens were KLH-fusions (see Figure S3). For all three hybridoma cell lines, antibody variable domain sequencing was performed by GenScript as a service (for sequencing results see Supplemental Information).

### Generated Anti-AMP Antibodies Are Highly Specific for AMPylation

**Figure 2:**
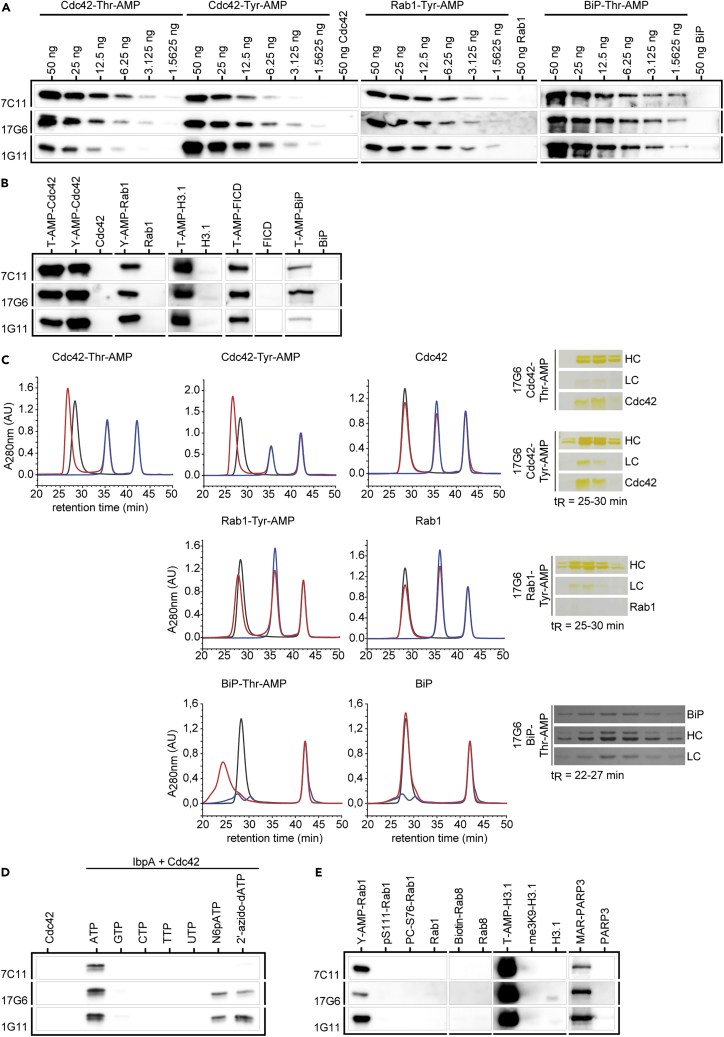
Evaluation of Specificity and Selectivity of Generated Anti-AMP Antibodies (A) Detection limits of AMPylated recombinant protein by the monoclonal anti-AMP antibodies in WB. Dilution rows starting from 50 ng recombinant Cdc42-Thr-AMP, -Tyr-AMP, Rab1-Tyr-AMP, and BiP-Thr-AMP, respectively, were analyzed in WB using all three monoclonal anti-AMP antibodies as indicated. Unmodified controls were loaded at 50 ng on the same blots as their modified counterparts. Cdc42, Rab1, and BiP represent three separate WBs. (B) Broadness of AMPylated target recognition by the monoclonal anti-AMP antibodies in WB. 50 ng recombinant protein was analyzed in WB using all three monoclonal anti-AMP antibodies as indicated. Rows represent identical WBs. (C) See also Figure S4. Native binding of AMPylated Cdc42, Rab1, and BiP by monoclonal anti-AMP antibody 17G6 analyzed by analytical size exclusion chromatography. 40 μg antigen for Cdc42 and Rab1, or 20 μg BiP was mixed with 60 μg antibody, including 50 μM vitamin B12 (t_R_ = 42.5 min) as internal standard. In black antibody 17G6 alone, in blue antigen alone as indicated, in red co-incubation of antibody 17G6 and antigen as indicated. Shifted antibody peaks (red) were fractionated and analyzed for co-elution of antibody (light chain, LC; heavy chain, HC) and antigen by silver-stained SDS PAGE (cropped rows represent identical gels). (D) See also Figure S5. Evaluation of specificity toward AMPylation by the monoclonal anti-AMP antibodies in WB. Cdc42 was NMPylated by IbpA using ATP, GTP, CTP, UTP, N6pATP, and 2′-azido-dATP. 50 ng recombinant Cdc42, unmodified or modified with nucleotides as indicated, was analyzed in WB using all three monoclonal anti-AMP antibodies as indicated. Rows represent identical WBs. (E) Cross-reactivity with other PTMs by the monoclonal anti-AMP antibodies in WB. 50 ng of recombinant protein was analyzed in WB using all three monoclonal anti-AMP antibodies as indicated. Rows represent identical WBs.

The three new anti-AMP antibodies 17G6, 7C11, and 1G11 were subsequently produced by roller bottle cell culture, purified from the supernatant via Protein A affinity capture, and tested for their performance in the recognition of denatured protein targets via WB. Here, sensitivity and detection limits, specificity toward AMPylation as opposed to incorporation of other nucleotides, and cross-reactivity with other PTMs were evaluated. In addition, native binding as previously shown by ELISA was confirmed by protein complex formation between the antibodies and AMPylated antigens on size exclusion chromatography. To determine the detection limits of recognition (Figure 2A), we tested all three antibodies in WB against a dilution series of recombinant Cdc42-Tyr-AMP, -Thr-AMP, Rab1-Tyr-AMP, and BiP-Thr-AMP, which were enzymatically modified by IbpA, VopS, DrrA, and FICD, respectively, in the presence of ATP and Mg^2+^. All antibodies showed similar performance on all the targets and modified side chains: all three antibodies were able to recognize as little as 2 ng or even lower amounts of AMPylated protein, while showing no recognition of the unmodified controls (50 ng). Antibody 1G11 detected modified Cdc42 at the Thr side chain with less sensitivity than at the Tyr side chain, and 7C11 detected Tyr-modified GTPases with less sensitivity than Thr-modified protein. Antibody 17G6 did not show a preference for a specific AMPylated side chain.

Next, we aimed to confirm backbone-independent recognition of AMPylation by applying all three antibodies to a broad range of AMPylated targets (Figure 2B), and indeed, the recognition of AMPylation was not limited to small GTPases. AMPylated FICD, BiP, and H3 were also recognized, representing very different protein classes, sizes, and folds. It therefore seems likely that other proteins will be recognized as well, which is a crucial prerequisite for target identification. Native binding of the antibodies to their modified antigens was investigated by complex formation with different AMPylated proteins using size exclusion chromatography (Figures 2C and S4). The shift of retention time of the antibody peaks upon incubation with AMPylated proteins toward higher molecular weight but no shift for incubation with non-modified counterparts for all antibodies illustrates strong and specific binding of modified targets. The shifted antibody peaks were collected by fractionation and analyzed by SDS PAGE for their co-elution with the antigens. Indeed, in case of AMPylated antigens, the antibodies co-eluted with their antigens. In this experiment, the same preferences for side chains were observed as already shown from studies by WB (denaturing conditions): antibody 1G11 shows a preference for AMPylated tyrosine, exemplified by a striking peak shift upon Rab1-Tyr-AMP binding but little shift for Cdc42-Thr-AMP (see Figure S4). Antibody 17G6 shows broad recognition of all modified targets, including a very prominent peak shift upon incubation with AMPylated BiP (Figure 2C), whereas 7C11 prefers threonine AMPylation and does not show binding of Rab1-Tyr-AMP (Figure S4). Notably, Rab1-Tyr-AMP appears to be a difficult antigen for native as well as denatured recognition by the new antibodies: Already during selection, Rab1-Tyr-AMP recognition in WB was one of the main hurdles for most candidates, and there were only few candidates who showed a strong signal in WB.

To test the antibodies' specificity toward the transferred nucleotide and their ability to differentiate AMPylation from, e.g., GMPylation, recombinant IbpA was used to introduce UMPylation, GMPylation, CMPylation, and TMPylation onto Cdc42 (Mattoo et al., 2011) (Figure 2D). In addition, the recognition of two reactive ATP analogs that have been previously described in the context of AMPylation, N6-Propargyl-ATP (N6pATP) (Grammel et al., 2011; Yu et al., 2014) and 2′-Azido-2′-dATP (Wang and Silverman, 2016), were tested (for mass spectrometric [MS] verification of incorporation see Figure S5). All antibodies successfully differentiated between the nucleotides and specifically recognized AMPylation in Cdc42. Using ATP analogs instead of ATP, we could confirm that the antibodies are also sensitive to base and ribose modifications, and only antibodies 17G6 and 1G11 showed slight recognition of modified ATP analogs (Figure 2D). This preference for AMPylation suggests a recognition of the adenine ring system by the antibodies.

After proving that the antibodies are sensitive and specific for AMPylation, we tested various other common PTMs for their ability to cross-react with the antibodies to rule out false-positive signals from competing modifications (Figure 2E). We tested phosphorylated (pS111) and phosphocholinated (PC-S76) Rab1b in direct comparison to its AMPylation, as well as biotinylated Rab8, trimethylated (me3K9) H3.1 in direct comparison to its AMPylation, and mono-ADP-ribosylated (MARylated) PARP3. To our satisfaction, the antibodies did not recognize phosphorylation, phosphocholination, biotinylation, or trimethylation on the chosen example proteins. However, the antibodies cross-reacted with MARylation on PARP3, most likely recognizing the present adenosine moiety.

### Anti-AMP Antibodies Can Shift Bias between AMPylation and MARylation

**Figure 3:**
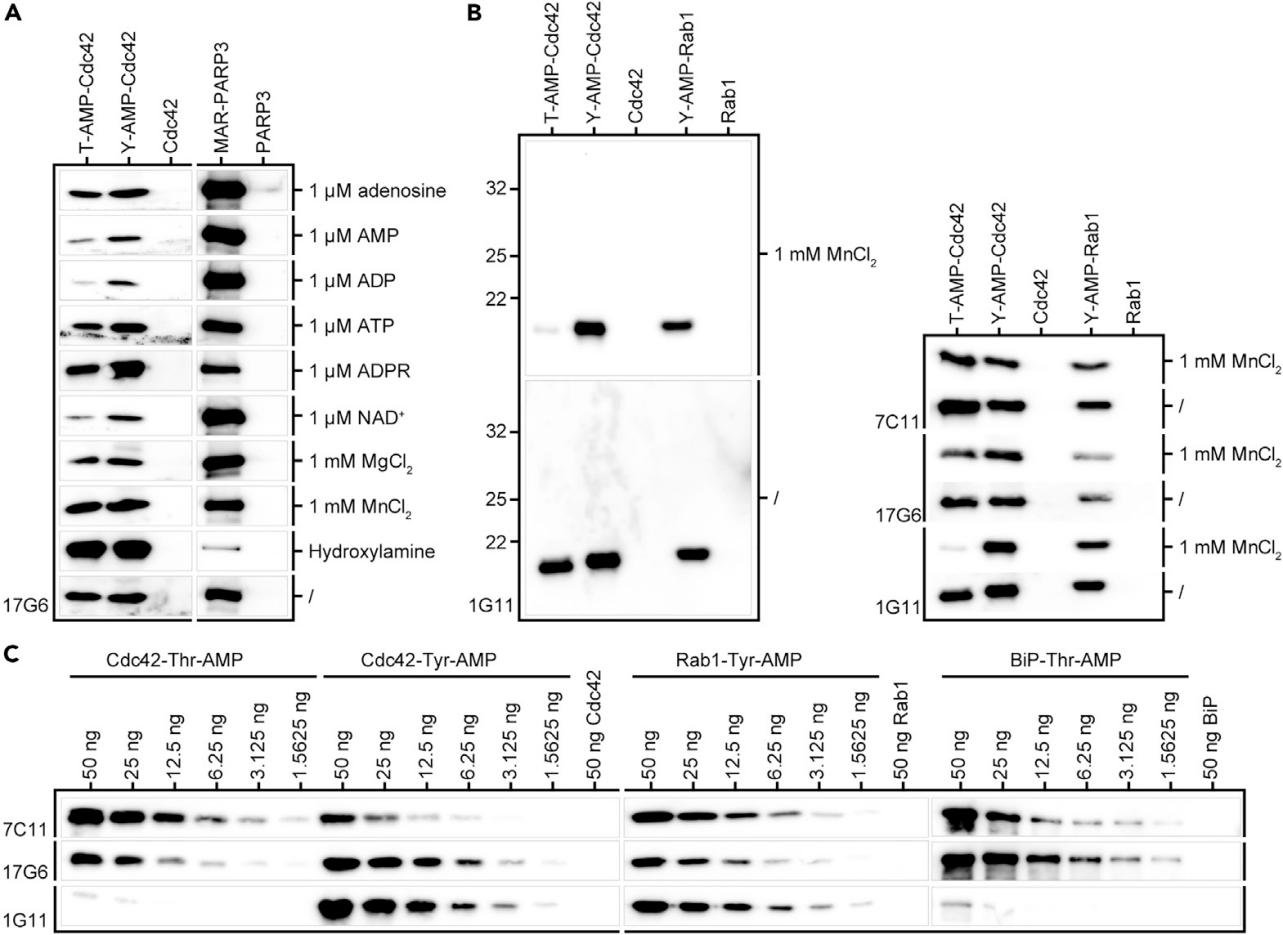
Detection of AMPylation and MARylation in the Presence of Additives and the Influence of MnCl2 on the Detection of AMPylation (A) Recognition of AMPylation versus MARylation by antibody 17G6 either in the presence of additives as indicated during primary antibody incubation or after hydroxylamine treatment. 50 ng recombinant Cdc42-Thr-AMP, -Tyr-AMP, as well as mono-ADP-ribosylated PARP3 and their unmodified counterparts as indicated were analyzed in WB using 17G6. Additives as indicated, with the exception of hydroxylamine, were added during incubation with primary anti-AMP antibody overnight at 4°C. Hydroxylamine treatment to cleave off ADP-ribosylation at Asp and Glu residues took place for 8 h after blotting before primary antibody incubation. Rows represent identical WBs. (B) Influence of 1 mM MnCl_2_ on antibody background and side chain specificity. 50 ng recombinant protein as indicated were analyzed in WB using all three monoclonal anti-AMP antibodies in the presence or absence of 1 mM MnCl_2_ during primary antibody incubation as indicated. Rows represent identical WBs. Left panel: exemplary full representation of the cropped rows of 1G11 (right) to illustrate change in background. (C) Detection limits under the influence of 1 mM MnCl_2_. Dilution rows starting from 50 ng recombinant Cdc42-Thr-AMP, -Tyr-AMP, Rab1-Tyr-AMP, and BiP-Thr-AMP, respectively, were analyzed in WB using all three monoclonal anti-AMP antibodies as indicated in the presence of 1 mM MnCl_2_ during primary antibody incubation. Unmodified controls were loaded at 50 ng on the same blots as their modified counterparts. Cdc42, Rab1, and BiP represent three separate WBs.

Our findings show that the developed anti-AMP antibodies also detect mono-ADP-ribosylation as exemplified by auto-modified PARP3 (Figure 2E). We therefore screened different additives to the primary antibody incubation step during WB for their ability to abrogate reactivity with ADP-ribosylation, while still keeping the recognition of AMPylation intact (Figure 3A). Adenine, AMP, ADP, ATP, ADP-Ribose (ADPR), and nicotinamide adenine dinucleotide (NAD^+^) were selected for their similarity to both modifications and their potential ability to compete with binding of ADP-ribosylation or AMPylation and displace modified proteins. MnCl_2_ and MgCl_2_ were chosen as divalent cations for their potential to complex the negatively charged diphosphate present in ADP-ribosylation but not AMPylation, thereby shielding negative charge that could potentially be relevant for antibody binding. Hydroxylamine treatment of the membrane after blotting reportedly results in specific cleavage of ADP-ribosylation at aspartate and glutamate side chains (Moss et al., 1983), but has not been previously tested regarding the stability of AMPylation. None of the tested additives were able to selectively reduce reactivity toward neither AMPylation nor MARylation, without significantly reducing overall sensitivity of the antibodies at the same time. Nevertheless, AMP, ADP, ATP, and NAD^+^ were able to reduce AMPylation signals to some extent, whereas the MARylation signal remained largely unaffected. However, keeping in mind that PARP3 has 14 reported auto-MARylation sites (Vyas et al., 2014), whereas Cdc42 is single AMPylated, this loss of signal in AMPylation but not MARylation might be due to the multiple modifications on MAR-PARP3 and therefore not be easily translatable toward other single ADP-ribosylated proteins, where these additives might also result in signal loss. As expected, hydroxylamine treatment resulted in a strong loss of MARylation signal due to cleavage of the ADP-ribosyl group. By contrast, the AMPylation signal remained entirely unaffected. The residual signal of auto-modified PARP3 is most likely resulting from the two reported auto-modification sites at lysine6 and lysine37 (Vyas et al., 2014) that will not be cleaved by hydroxylamine.

The addition of 1 mM MnCl_2_ during the primary antibody incubation step, although not affecting signal intensity, resulted in a significantly reduced background. Strikingly, the addition of MnCl_2_ also resulted in a sharply enhanced tyrosine specificity for antibody 1G11 in the presence of MnCl_2_ (Figure 3B). The addition of MnCl_2_ was therefore further evaluated in regard to the previously tested detection limits of the antibodies. We confirmed that the detection limits of antibodies 17G6 and 7C11 toward AMPylated antigens was not significantly changed by addition of MnCl_2_, whereas 1G11's ability to detect Cdc42-Thr-AMP as well as BiP-Thr-AMP was greatly diminished (Figure 3C).

In summary, our newly developed antibodies are a combined tool for detection of AMPylation and mono-ADP-ribosylation. By addition of MnCl_2_ to the primary antibody incubation steps in WB, the background of the antibodies can be significantly reduced and 1G11 shows pronounced tyrosine specificity. Treating membranes with hydroxylamine after blotting results in the cleavage of glutamate- and aspartate-linked ADP-ribosylation, whereas AMPylation remains unaffected. Therefore, the combination of all three antibodies with addition of MnCl_2_ and hydroxylamine treatment results in a tool kit that is able to sensitively detect ADP-ribosylation and AMPylation, to differentiate between the two, and, in the case of AMPylation, not only to recognize targets in general but also to give information on their modified side chains.

### Generated Anti-AMP Antibodies Recognize Diverse Cellular AMPylation

After thorough characterization and evaluation of our produced antibodies on purified and MS-confirmed antigens, we next evaluated the antibody performance on cell lysates in known contexts under denatured as well as native conditions. The reproduction of previous results with these new tools is crucial for the trust in future findings and a smooth transition from previously used tools.

**Figure 4:**
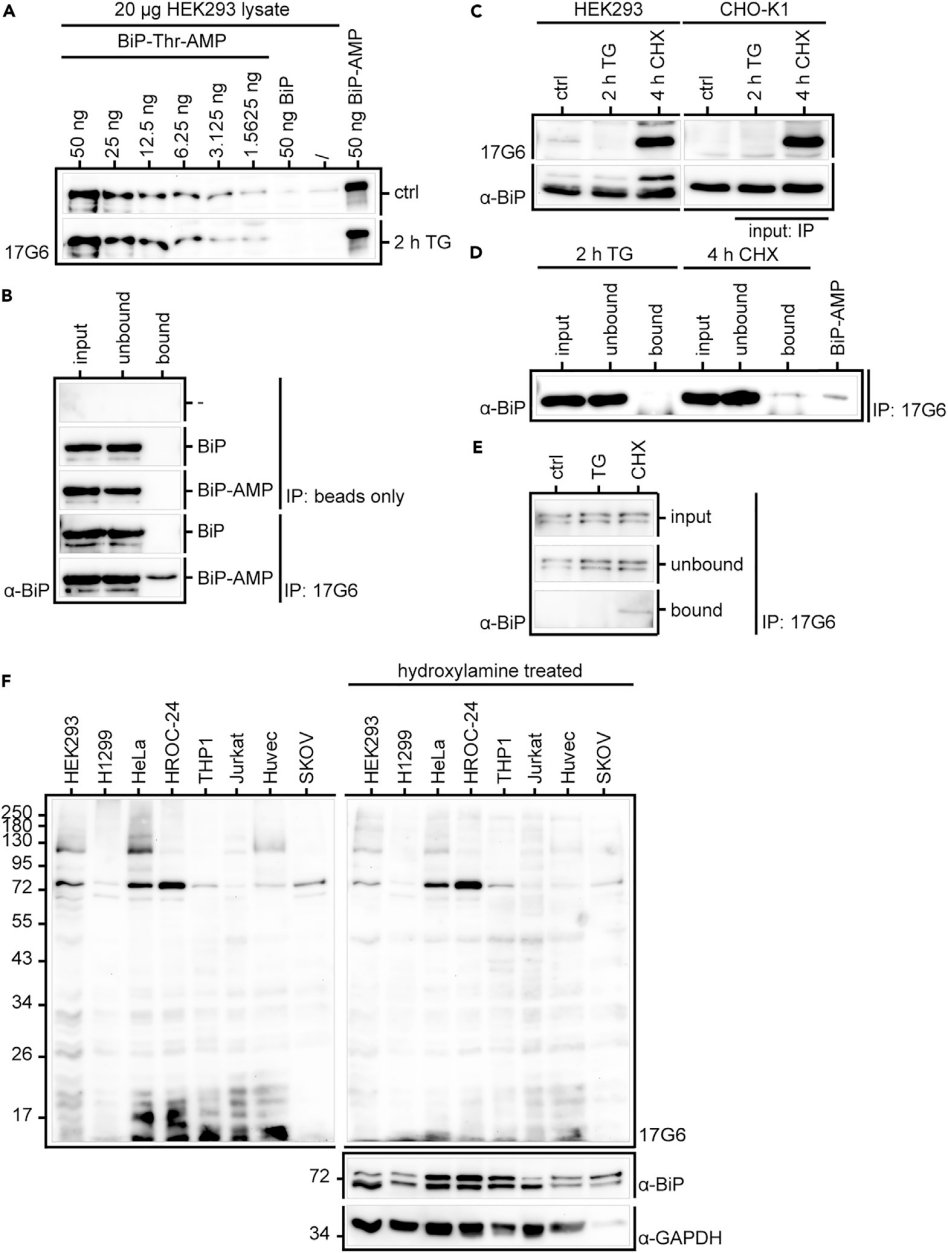
Evaluation of Generated Anti-AMP Antibodies in IP and Application to Whole-Cell Lysate (A) Analysis of BiP-AMPylation detection levels in HEK293 lysate, either untreated (ctrl) or treated by TG, in WB by antibody 17G6 in the presence of MnCl_2_. 20 μg HEK293 lysate was spiked with a titration of recombinant BiP-AMP or 50 ng unmodified BiP as indicated. Unspiked lysate and 50 ng purified BiP-AMP alone served as negative and positive ctrl, respectively. Rows represent identical WBs. (B) IP with antibody 17G6 on recombinant BiP-AMP. 20 μg recombinant BiP or BiP-AMP in 500 μL was precipitated by 10 μg 17G6 and Protein A/G Magnetic Beads or beads alone. Bound protein was eluted with 100 μL 1× Laemmli. Samples with and without addition of 17G6 were treated identically. 7.5 μL each of input and unbound samples supplemented with 5× Laemmli buffer and 2.5 μL elution were analyzed in WB using an anti-BiP antibody. Rows of IP: beads only and IP: 17G6 represent the same WB. (C) Analysis of BiP-AMPylation upon ER stress by TG or CHX in WB. 20 μg total protein lysate of HEK293 and CHO-K1 cells, untreated or treated with either TG for 2 h or CHX for 4 h was analyzed in WB using antibody 17G6 in the presence of MnCl_2_, before the blot was stripped and reprobed with an anti-BiP antibody. 10 ng recombinant Cdc42-Tyr-AMP as well as unmodified Cdc42 was blotted as control (see Supplemental Information). The indicated CHO-K1 samples served as input for the IP in Figure 4D. All samples represent one WB. (D) IP of endogenous BiP-AMP with antibody 17G6 from TG- and CHX-treated (as indicated) CHO-K1 cell lysates. 1 mg (in 500 μL) total protein lysate of CHO-K1 cells treated with either TG for 2 h or CHX for 4 h was precipitated by 10 μg 17G6 and Protein A/G Magnetic Beads. Bound proteins were eluted with 100 μL 1× Laemmli and concentrated to 20 μL. 5 μL each of input and unbound sample supplemented with 6× Laemmli buffer and 20 μL concentrated elution were analyzed in WB using an anti-BiP antibody. 50 ng recombinant BiP-AMP was blotted as control. (E) IP of endogenous BiP-AMP with antibody 17G6 from untreated (ctrl) and TG- and CHX-treated (as indicated) CHO-K1 cell lysates after methanol/chloroform precipitation. 250 μg total protein lysate of CHO-K1 cells, untreated or treated with either TG for 2 h or CHX for 4 h, was precipitated by methanol/chloroform precipitation. Denatured protein from the resuspended protein pellet was immunoprecipitated by 5 μg 17G6 in 125 μL Protein A/G Magnetic Beads. Bound proteins were eluted with 50 μL 1× Laemmli. 5 μL each of input and unbound sample supplemented with 6× Laemmli buffer and 20 μL of elution were analyzed in WB using an anti-BiP antibody. All rows represent the same WB. 50 ng recombinant BiP-AMP and BiP were blotted as control. (F) See also Figures S6 and S7. Analysis of AMPylation patterns in various immortalized and cancer cell lines in WB by antibody 17G6 in the presence of MnCl_2_. 20 μg total protein lysate per lane of cell lines as indicated was probed with antibody 17G6 using 1 mM MnCl_2_ as additive during primary antibody incubation. Afterward the blot was treated with 1 M hydroxylamine to cleave ADP-ribosylation at aspartate and glutamate residues, and reprobed with antibody 17G6 in the presence of 1 mM MnCl_2_. The identical blot was stripped and reprobed with antibodies against BiP and GAPDH as loading control.

Previously, it was shown that BiP AMPylation by FICD is lost in cells upon stimulation of ER stress, e.g., by thapsigargin (TG) (Ham et al., 2014; Preissler et al., 2015). Cycloheximide (CHX), in contrast, will stall protein production, therefore relieving the ER of protein load, causing a significant increase in BiP AMPylation. To reproduce these findings, we first set out to confirm the detection levels of BiP-AMP in the environment of cell lysates (Figure 4A). Accordingly, we spiked 20 μg HEK293 lysate per lane, either untreated (ctrl) or treated for 2 h with TG to remove endogenously AMPylated BiP, with a titration of recombinant BiP-AMP. As controls, the lysates were either additionally spiked with 50 ng unmodified BiP or not. Probing the WB with antibody 17G6 in the presence of MnCl_2_ we could confirm the same low detection limits of up to 1.5 ng for BiP-AMP in lysate as previously shown in Figure 3C on purified protein. To verify that the antibodies' previously confirmed ability to bind native AMPylated protein would translate into a successful IP, we applied antibody 17G6 in the context of BiP-AMPylation (Figure 4B). Using recombinant BiP and BiP-AMP showed that IP is dependent on the presence of antibody 17G6, as beads alone do not pull down either modified or unmodified BiP, and specific for AMPylation, as the non-modified BiP is not precipitated by antibody 17G6.

Having thus established that antibody 17G6 is capable of detecting BiP-AMP in cell lysates at low amounts in WB, as well as of binding BiP-AMP in IP, we applied both techniques to the aforementioned BiP-AMPylation in the context of ER stress. We reproduced these findings, representative for all our antibodies, with antibody 17G6 and MnCl_2_ as an additive in HEK293 as well as CHO-K1 cells (Figure 4C) and could confirm these previously published results (Ham et al., 2014; Preissler et al., 2015). Although the initial endogenous amount of AMPylated BiP appears slightly higher in HEK293 than CHO-K1 cells, both cell lines similarly react to TG treatment for 2 h, with the complete removal of BiP-AMPylation, and to CHX treatment for 4 h with a strong increase in AMPylated BiP.

To verify that antibody 17G6's ability to immunoprecipitate recombinant BiP (Figure 4B) would successfully translate into an IP in the environment of cell lysate, we applied antibody 17G6 to the aforementioned (Figure 4C) TG as well as CHX-treated CHO-K1 cells (Figure 4D). The detection of a successful IP from cell lysates was hampered by the detection limit of the anti-BiP antibody, which did not recognize BiP at less than 50 ng per lane. With detection limits of the anti-AMP antibodies far lower, the difficulty to detect a band by the anti-AMP antibody 17G6 in untreated CHO-K1 whole-cell lysates (in WB, Figure 4C) hints at very low endogenous amounts of AMPylated BiP, whereas the less sensitive anti-BiP antibody leads to a more pronounced signal. Therefore, we had to assume that the percentage of AMPylated BiP in the untreated CHO-K1 cell lysates was very low. Consequently, AMPylation was stimulated by CHX treatment to create enough pull-down material for detection with anti-BiP antibody. Antibody 17G6 was able to immunoprecipitate AMPylated BiP from CHX-treated cell lysates, whereas the detection by anti-BiP antibody remains rather faint (Figure 4D). This effect may be explained by the high intracellular concentrations of nucleotides such as AMP, ADP, and ATP that will compete for binding with the anti-AMP antibodies. To overcome this challenge, we extracted the proteome from untreated (ctrl) as well as TG- and CHX-treated CHO-K1 cell lysates by methanol/chloroform precipitation to eliminate competing nucleotides. Denatured AMPylated BiP was immunoprecipitated from the resuspended protein pellets by antibody 17G6 (Figure 4E). Enriched BiP-AMP could be detected in the bound sample from CHX-treated CHO-K1 cells at much higher amounts in relation to the input than without methanol/chloroform precipitation (Figure 4D). The yield and efficiency of the IP of AMPylated BiP was thereby greatly improved.

After the thorough characterization of our antibodies' performance in WB, we asked the question whether these sensitive tools were able to detect new AMPylation bands in cell lysates. We therefore screened a number of available immortalized and cancer cell lines for occurrence of AMPylation bands using our newly developed monoclonal antibodies (Figures 4F and S6). Indeed, our anti-AMP antibodies were able to detect a multitude of bands in the range of 58–245 kDa and 11–22 kDa. Strikingly, some bands differed among cell lysates, whereas others were distinctive and reoccurring. Some cell lines, such as THP-1, show very little to no AMPylation signals, whereas other cell lines such as HeLa, HEK293, and HROC-24 cells show strong AMPylation signals. Treatment of membranes with hydroxylamine to cleave off ADP-ribosylation at aspartate and glutamate residues does remove bands at 11–22 kDa but does not significantly diminish the bands at 70–80 kDa. Furthermore, these bands are not detected by the anti-pan-ADPR-binding reagent (Merck) (Figure S7), or tyrosine-specific anti-AMP antibody 1G11 (Figure S6), but both anti-AMP antibodies 7C11 (Figure S6) and 17G6 (Figure 4F), strongly suggesting AMPylation at threonine residues. As antibody 1G11 switches between a general recognition of all AMPylated side chains (Figures 2A and 2B) and a tyrosine-specific recognition in the presence of MnCl_2_ (Figures 3B and 3C), we reprobed the blot previously incubated with antibody 1G11 in the presence of MnCl_2_ with antibody 1G11 alone (Figure S6), both before and after hydroxylamine treatment. In both cases, a band pattern very similar to the blots of 7C11 and 17G6 arose, further supporting a predominance of threonine AMPylation in human cells. The reoccurring band at 70 kDa, most likely representing BiP-AMP, strongly differs in intensity among cell lysates: whereas H1299, THP-1, Jurkat, and Huvec cells do not show this band at all, it is strongly represented in HeLa, HROC-24, and HEK293 cells. Probing the same cell lysates with both commercial anti-AMP antibodies (Merck) resulted in no significant bands for the anti-Thr-AMP antibody (Figure S7) as expected based on its performance on recombinant AMPylated protein (Figure 1A). Although the anti-Tyr-AMP did detect a band at around 30 kDa in THP-1 as well as Jurkat cell lysates (Figure S7), it did not recognize the positive control of Cdc42-Tyr-AMP, thus the significance of the band remains doubtful.

## Discussion

Here, we report and characterize three new monoclonal anti-AMP antibodies that recognize AMPylation independent of the protein backbone. To reduce the inherent batch-to-batch variability of the previously published polyclonal antibodies, as well as generate defined specificities, we created monoclonal antibodies in mice. The reproducibility crisis of antibodies in recent years (Baker, 2015), as well as the limitations of commercially available anti-AMPylation-antibodies (Hao et al., 2011) (Sigma-Aldrich ABS184 and 09-890) let us to perform a thorough evaluation of the new monoclonal antibodies' performance in two different applications. For denatured recognition, we tested sensitivity, specificity, and cross-reactivity of our antibodies in WBs. For native recognition, we studied complex formation of the antibody with different modified targets by size exclusion chromatography, as well as confirmed native binding in IP experiments. In WB, the antibodies show cross-reactivity toward MARylation. Although hydroxylamine treatment of membranes proves helpful in cleaving off MARylation at aspartate and glutamate residues, it might not always completely remove the MARylation signal, either due to the abundance of the modified protein or due to modification on residues other than aspartate and glutamate, such as lysine. Therefore, a careful evaluation of findings with orthogonal methods such as MS is crucial. In IP, low abundance of AMPylation might hinder efficient enrichment, especially if high amounts of competing nucleotides or mono-ADP-ribosylation are present. A proteome extraction by methanol/chloroform protein precipitation might help to get rid of competing nucleotides and enrich AMPylated protein in a denatured state.

The antibodies were generated with the help of an AMPylated synthetic peptide with reduced backbone complexity. A major bottleneck in antibody generation based on synthesized peptides, which is also reported for the generation of other anti-PTM antibodies such as anti-phospho antibodies (Archuleta et al., 2011), is the phenomenon of predominantly positive peptide ELISA readings against modified hapten that do not translate to a positive WB performance. Common procedure is to only select via ELISA against the modified hapten. According to Archuleta et al. (2011), this method selects antibodies whose performance fails in other applications in 25%–50% cases. However, in our selection process we observed a high correlation between positive ELISA readings against modified protein, which we performed in addition to peptide ELISA, and good WB performance. The inclusion of a native AMPylated protein in the form of Cdc42-Thr-AMP in the ELISA screening process allowed us to generate monoclonal antibodies combined with efficient preselection of candidates before WB evaluation. We therefore recommend including native modified protein in the ELISA screening process for all anti-PTM antibodies.

First efforts in the creation of anti-AMP antibodies were undertaken in 1984 by fusing AMP directly to the carrier protein BSA (Chung and Rhee, 1984), thus generating murine monoclonal antibodies that were purified from ascitic fluid and employed in the purification of AMPylated glutamine synthetase. Later on, other antibodies were accidentally produced by aiming for ADP-ribose antibodies, where the hapten was degraded to contain AMP, resulting in antibodies recognizing free 5′-AMP (Bredehorst et al., 1978; Meyer and Hilz, 1986). Hao et al. (2011) achieved polyclonal antibodies by immunization of rabbits with a synthetic seven-amino-acid-long Rac1-peptide containing a threonine AMPylation (now commercially available as Anti-pan-AMPylated Threonine Antibody 09-890, Sigma-Aldrich Merck). After depletion with tyrosine-AMPylated protein the serum was reported to detect threonine AMPylation independently of protein backbone and structure, in WB as well as in IP. The most recent antibody was produced by an AMPylated Rab1b peptide of 13 amino acids coupled to KLH in rabbit, resulting in polyclonal serum, aided again by efficient synthesis of AMPylated peptides (Smit et al., 2011). However, both published rabbit antibodies are hampered by low sensitivity and little characterization, especially concerning cross-reactivity with other PTMs and recognition of targets outside the protein class of small GTPases or BiP. In addition, all recently developed antibodies are polyclonal with the accompanying challenges of batch-to-batch reproducibility and reliability of tool development on the basis of that antibody. Considering the special challenges connected with the generation of antibodies that target PTMs (Hattori and Koide, 2018), and their necessity for extensive characterization, polyclonal antibodies are not an ideal choice. A stringent retesting of every new batch regarding proper AMP recognition and lack of cross-reactivity would have to be performed before application to cell lysates. Previous antibodies therefore represent no general recognition tool of AMPylation, especially if searching for new targets and effects where the number of potential false-negative or false-positive findings would render them unreliable. Our experiments show that all commercially available anti-AMP antibodies offer no broad recognition of targets, despite claiming to recognize AMPylation backbone independently, and are exhibiting a significant amount of false-positive and negative reactions in our in-house testing. The limitations in performance and cross-reactivity of both anti-ADPR reagents and anti-AMPylation antibodies in combination causes the danger of false-positives for ADP-ribosylation as well as false-negatives in AMPylated proteins, and a bias in AMPylation research toward small GTPases and threonine modifications (Figure 1A). As many researchers lack suitable positive and negative controls of protein of interest, these performance failures might never be detected.

Little is known about AMPylation in eukaryotic cells outside the modification of BiP in the context of ER stress. In accordance with recent publications (Kielkowski et al., 2020; Sreelatha et al., 2018), the application of our new monoclonal antibodies to cell lysates of immortalized and cancer cell lines hints at a much stronger prevalence of AMPylation than ever perceived. The limited number of tools, especially in medium to high throughput, has hampered reliable detection of AMPylation in cellular systems. Our antibodies expand the available toolbox by offering sensitive detection and enrichment of AMPylation, while at the same time requiring little resources that might hamper applicability in a standard laboratory. These antibodies therefore open new opportunities in an expanding research field. The recent antibody “reproducibility crisis,” especially in regard to antibodies targeting PTMs (Baker, 2015; Egelhofer et al., 2011), suggested a thorough characterization of the AMPylation specificity and sensitivity of our new monoclonal antibodies in the applications WB, ELISA, and IP. With their high sensitivity and broad target recognition, they overcome the limitations of previously published anti-AMP antibodies and create opportunities for new target identification and study of cellular AMPylation. Our data suggest that they can successfully be used for enrichment of AMPylated proteins and peptides for MS to overcome the limitation of low occurrence of AMPylation in proteomic studies. As all three monoclonal antibodies are sequenced (see Supplemental Information), thereby enabling recombinant antibody production, they form a good basis for long-term reproducibility in AMPylation research.

### Limitations of the Study

The present study generated and characterizes three new monoclonal anti-AMP antibodies in the applications WB, ELISA, and IP. In WB, the antibodies show cross-reactivity toward MARylation: although hydroxylamine treatment of membranes proves helpful in cleaving off MARylation at aspartate and glutamate residues, it might not always completely remove MARylation signal, either due to a high abundance of the modified protein or modification on residues other than aspartate and glutamate, such as lysine. Therefore, a careful evaluation of findings with orthogonal methods such as MS, etc., is crucial. In IP, low abundance of AMPylation might hinder efficient enrichment, especially if high amounts of competing nucleotides or mono-ADP-ribosylation are present. Other applications such as immunofluorescence and immunohistochemistry remain uncharacterized.

### Resource Availability

#### Lead Contact

Further information and requests for resources and reagents should be directed to and will be fulfilled by the Lead Contact, Aymelt Itzen (a.itzen@uke.de).

#### Materials Availability

Plasmids generated in this study are available upon request. Reasonable requests for the generated monoclonal anti-AMP antibodies will be fulfilled by the Lead Contact, Aymelt Itzen.

#### Data and Code Availability

This study did not generate or analyze datasets or code. All uncropped WB and gel images along with their dynamic range are depicted in the Supplemental Information (Data S1). The antibody sequencing results of all three monoclonal anti-AMP antibodies are included in the Supplemental Information.

## Methods

All methods can be found in the accompanying Transparent Methods supplemental file.

## Supplemental Information

Document S1. Transparent Methods, Figures S1–S7, Tables S1 and S2, and Data S1

## Footnotes

Supplemental Information can be found online at https://doi.org/10.1016/j.isci.2020.101800.

## References

[bib1] Albers M.F., Van Vliet B., Hedberg C. (2011). Amino acid building blocks for efficient Fmoc solid-phase synthesis of peptides adenylylated at serine or threonine. Org. Lett..

[bib2] Archuleta A.J., Stutzke C.A., Nixon K.M., Browning M.D., Kalyuzhny A.E. (2011). Optimized protocol to make phospho-specific antibodies that work. Signal transduction immunohistochemistry: methods and protocols, methods in molecular biology.

[bib3] Baker M. (2015). Blame it on the antibodies. Nature.

[bib4] Bredehorst R., Ferro A.M., Hilz H. (1978). Production of antibodies against ADP-ribose and 5’-AMP with the aid of N6-carboxymethylated ADP-ribose conjugates. Eur. J. Biochem..

[bib5] Chung H.K., Rhee S.G. (1984). Separation of glutamine synthetase species with different states of adenylylation by chromatography on monoclonal anti-AMP antibody affinity columns. Proc. Natl. Acad. Sci. U S A.

[bib6] Egelhofer T.A., Minoda A., Klugman S., Lee K., Kolasinska-Zwierz P., Alekseyenko A.A., Cheung M.S., Day D.S., Gadel S., Gorchakov A.A. (2011). An assessment of histone-modification antibody quality. Nat. Struct. Mol. Biol..

[bib7] Engel P., Goepfert A., Stanger F.V., Harms A., Schmidt A., Schirmer T., Dehio C. (2012). Adenylylation control by intra- or intermolecular active-site obstruction in Fic proteins. Nature.

[bib8] Grammel M., Luong P., Orth K., Hang H.C. (2011). A chemical reporter for protein AMPylation. J. Am. Chem. Soc..

[bib9] Ham H., Woolery A.R., Tracy C., Stenesen D., Krämer H., Orth K. (2014). Unfolded protein response-regulated Drosophila Fic (dFic) protein reversibly AMPylates BiP chaperone during endoplasmic reticulum homeostasis. J. Biol. Chem..

[bib10] Hao Y.H., Chuang T., Ball H.L., Luong P., Li Y., Flores-Saaib R.D., Orth K. (2011). Characterization of a rabbit polyclonal antibody against threonine-AMPylation. J. Biotechnol..

[bib11] Hattori T., Koide S. (2018). Next-generation antibodies for post-translational modifications. Curr. Opin. Struct. Biol..

[bib12] Khater S., Mohanty D. (2015). In silico identification of AMPylating enzymes and study of their divergent evolution. Sci. Rep..

[bib13] Kielkowski P., Buchsbaum I.Y., Kirsch V.C., Bach N.C., Drukker M., Cappello S., Sieber S.A. (2020). FICD activity and AMPylation remodelling modulate human neurogenesis. Nat. Commun..

[bib14] Kingdon H.S., Shapiro B.M., Stadtman E.R. (1967). Regulation of glutamine synthetase, VIII. ATP: glutamine synthetase adenylyltransferase, an enzyme that catalyzes alterations in the regulatory properties of glutamine synthetase. Proc. Natl. Acad. Sci. U S A.

[bib15] Mattoo S., Durrant E., Chen M.J., Xiao J., Lazar C.S., Manning G., Dixon J.E., Worby C.A. (2011). Comparative analysis of Histophilus somni immunoglobulin-binding protein A (IbpA) with other Fic domain-containing enzymes reveals differences in substrate and nucleotide specificities. J. Biol. Chem..

[bib16] Meyer T., Hilz H. (1986). Production of anti-(ADP-ribose) antibodies with the aid of a dinucleotide-pyrophosphatase-resistant hapten and their application for the detection of mono(ADP-ribosyl)ated polypeptides. Eur. J. Biochem..

[bib17] Moss J., Yost D.A., Stanley S.J. (1983). Amino acid-specific ADP-ribosylation. J. Biol. Chem..

[bib18] Müller M.P., Peters H., Blümer J., Blankenfeldt W., Goody R.S., Itzen A. (2010). The Legionella effector protein DrrA AMPylates the membrane traffic regulator Rab1b. Science.

[bib19] Plagemann P.G.W., Wohlhueter R.M. (1980). Permeation of nucleosides, nucleic acid bases, and nucleotides in Animal cells. Curr. Top. Membr. Transp..

[bib20] Preissler S., Rato C., Chen R., Antrobus R., Ding S., Fearnley I.M., Ron D. (2015). AMPylation matches BiP activity to client protein load in the endoplasmic reticulum. Elife.

[bib21] Preissler S., Rato C., Perera L.A., Saudek V., Ron D. (2016). FICD acts bifunctionally to AMPylate and de-AMPylate the endoplasmic reticulum chaperone BiP. Nat. Struct. Mol. Biol..

[bib22] Sanyal A., Chen A.J., Nakayasu E.S., Lazar C.S., Zbornik E.A., Worby C.A., Koller A., Mattoo S. (2015). A novel link between fic (filamentation induced by cAMP)-mediated adenylylation/AMPylation and the unfolded protein response. J. Biol. Chem..

[bib23] Smit C., Blümer J., Eerland M.F., Albers M.F., Müller M.P., Goody R.S., Itzen A., Hedberg C. (2011). Efficient synthesis and applications of peptides containing adenylylated tyrosine residues. Angew. Chem. Int. Ed..

[bib24] Sreelatha A., Yee S.S., Lopez V.A., Park B.C., Kinch L.N., Pilch S., Servage K.A., Zhang J., Jiou J., Karasiewicz-Urbańska M. (2018). Protein AMPylation by an evolutionarily conserved pseudokinase. Cell.

[bib25] Truttmann M.C., Cruz V.E., Guo X., Engert C., Schwartz T.U., Ploegh H.L. (2016). The Caenorhabditis elegans protein FIC-1 is an AMPylase that covalently modifies heat-shock 70 family proteins, translation elongation factors and histones. PLoS Genet..

[bib26] Vyas S., Matic I., Uchima L., Rood J., Zaja R., Hay R.T., Ahel I., Chang P. (2014). Family-wide analysis of poly(ADP-ribose) polymerase activity. Nat. Commun..

[bib27] Wang P., Silverman S.K. (2016). DNA-catalyzed introduction of azide at tyrosine for peptide modification. Angew. Chem. Int. Ed..

[bib28] Worby C.A., Mattoo S., Kruger R.P., Corbeil L.B., Koller A., Mendez J.C., Zekarias B., Lazar C., Dixon J.E. (2009). The fic domain: regulation of cell signaling by adenylylation. Mol. Cell.

[bib29] Yarbrough M.L., Li Y., Kinch L.N., Grishin N.V., Ball H.L., Orth K. (2009). AMPylation of Rho GTPases by Vibrio VopS disrupts effector binding and downstream signaling. Science.

[bib30] Yu X., Woolery A.R., Luong P., Hao Y.H., Grammel M., Westcott N., Park J., Wang J., Bian X., Demirkan G. (2014). Copper-catalyzed azide-alkyne cycloaddition (click chemistry)-based detection of global pathogen-host AMPylation on self-assembled human protein microarrays. Mol. Cell. Proteomics.

