# Monoclonal Anti-AMP Antibodies Are Sensitive and Valuable Tools for Detecting Patterns of AMPylation

**DOI:** 10.1016/j.isci.2021.102731

**Published:** 2021-06-26

**Authors:** Dorothea Höpfner, Joel Fauser, Marietta S. Kaspers, Christian Pett, Christian Hedberg, Aymelt Itzen

## Main text

(iScience *23*, 101800-1–101800-14; December 18, 2020)

As a result of an author oversight in the originally published version of this article, a citation for the creation of the HROC24 cells (Maletzki et al., 2012) was omitted. This error has now been corrected in the manuscript and transparent methods. The authors apologize for the error and any inconvenience that may have resulted.
