# Supplementary material for: Monoclonal Anti-AMP Antibodies Are Sensitive and Valuable Tools for Detecting Patterns of AMPylation
Source: iScience. 2020 Nov 13;23(12):101800. doi: 10.1016/j.isci.2020.101800 (PMC7704405; doi:10.1016/j.isci.2020.101800)
Supplement: Document S1. Transparent Methods, Figures S1–S7, Tables S1 and S2, and Data S1 [file mmc1.pdf]

iScience, Volume *23*

## Supplemental Information

# Monoclonal Anti-AMP Antibodies

# Are Sensitive and Valuable Tools

# for Detecting Patterns of AMPylation

**Dorothea Höpfner, Joel Fauser, Marietta S. Kaspers, Christian Pett, Christian Hedberg, and Aymelt Itzen**

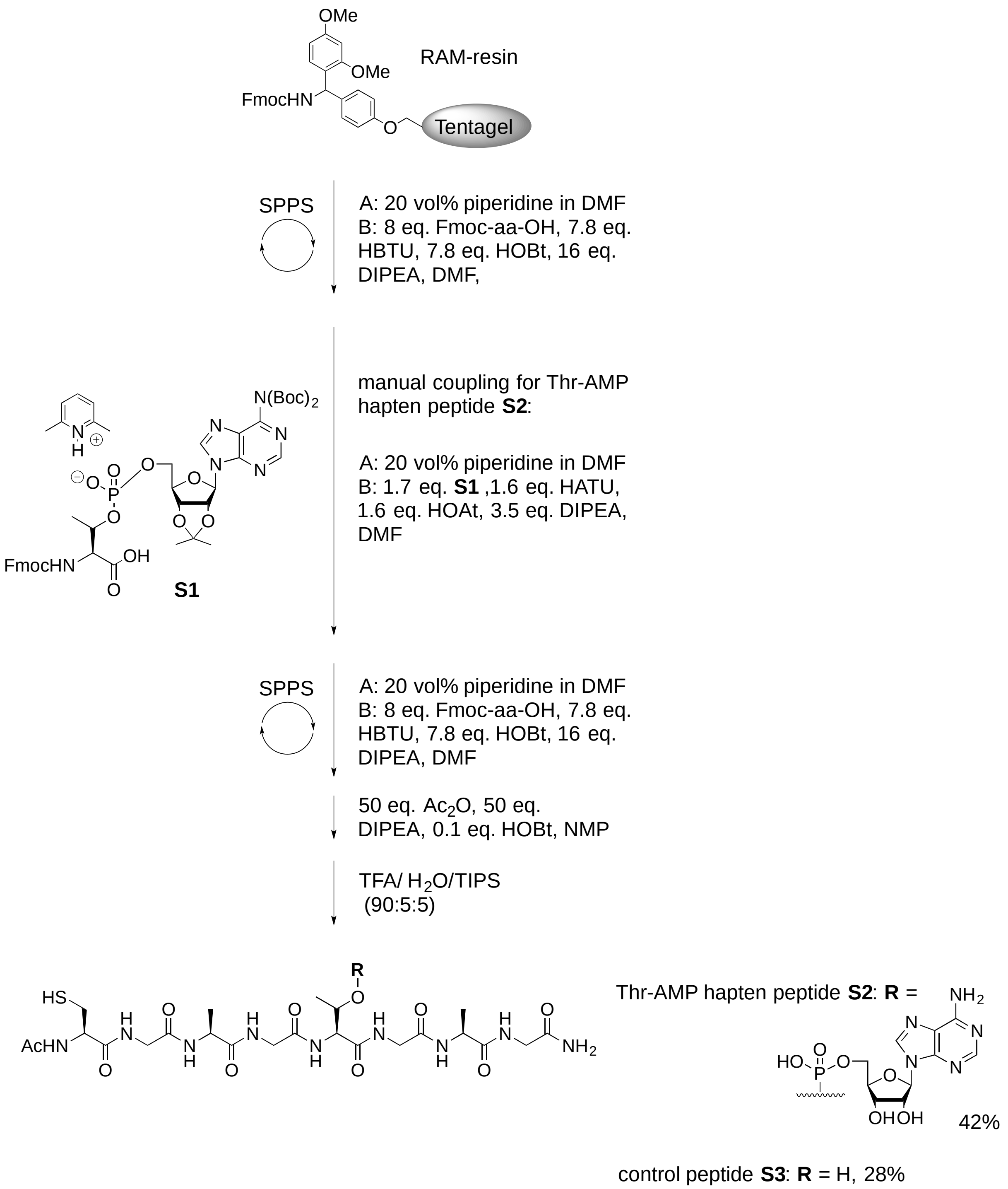


**Figure S1: Solid phase peptide synthesis scheme of peptides S2 and S3.** Related to Figure 1D.

**S2**

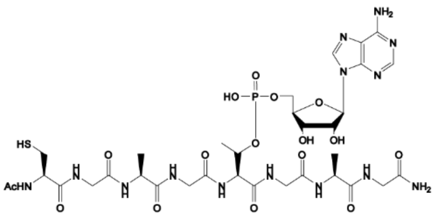

**S3**

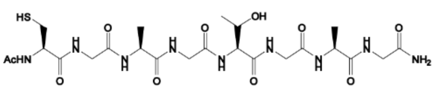

**S2** $^{1}$H-NMR

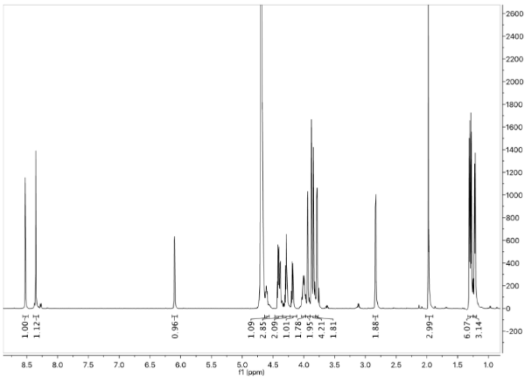

**S3** $^{1}$H-NMR

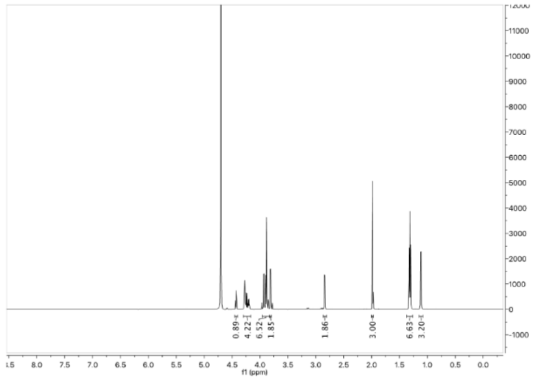

**S2** $^{13}$C-NMR

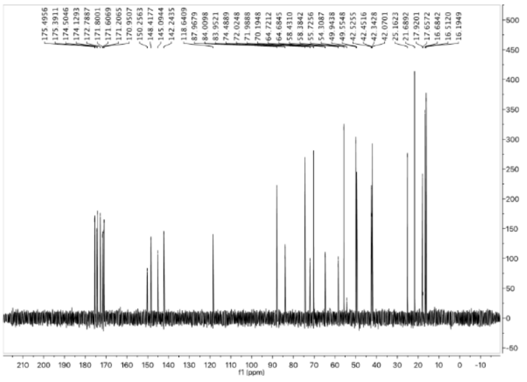

**S3** $^{13}$C-NMR

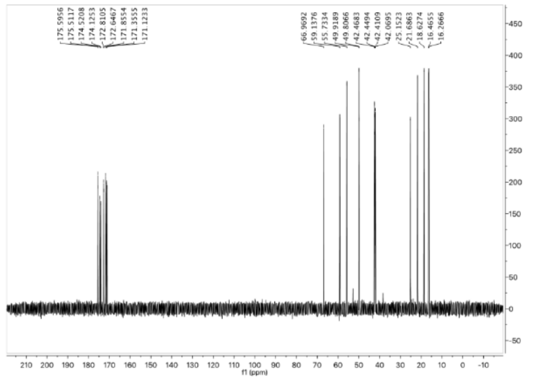

**S2** $^{31}$P-NMR

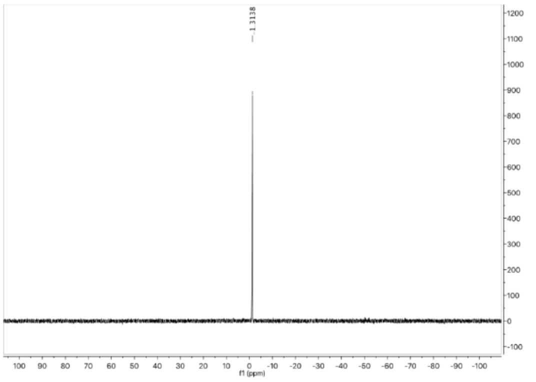

**S3** HPLC (UV 214 nm)

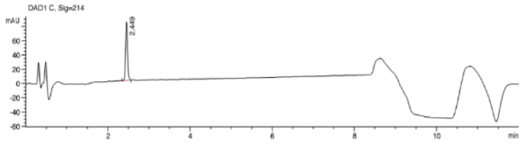


**S3** HR-ESI-MS

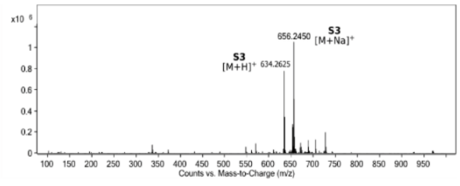


**S2** HPLC (UV 214 nm)

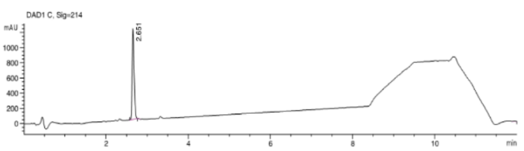


**S2** HR-ESI-MS

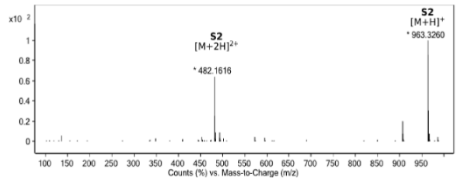


**Figure S2: Quality control of synthesized peptides S2 and S3.** Related to Figure 1D. Thr-AMP hapten peptide Ac-CGAGT(AMP)GAG-NH2 (**S2**) was synthesized on 100 mg (25 µmol) of Tentagel-S-RAM resin. Yield: 42% (10.0 mg, 10.4 µmol). Analytical **HPLC** $R_t$ = 2.651 min (A/B, (98 : 2) → (75 : 25), 500 µL/min, 7 min); Preparative HPLC $R_t$ = 8.945 min (A/B, (97.5 : 2.5) → (50 : 50), 20 mL/min,

10 min). **HR-ESI-MS**, m/z: 482.1616 ($[M+2H]^{2+}$, calc. 482.1606), 963.3260 ($[M+H]^+$, calc. 963.3139). **$^1$H NMR** (600 MHz, $D_2O$) δ: 8.52 (s, 1H), 8.35 (s, 1H), 4.62-4.59 (m, 1H), 4.43-4.39 (m, 3H), 4.31-4.27 (m, 2H), 4.18 (q, J = 7.3 Hz, 1H), 4.04-3.97 (m, 2H), 3.94 ($s_{br}$, 2H), 3.87 (s, 2H), 3.84 (s, 2H), 3.79 (d, J = 5.2 Hz, 2H), 2.83 (d, J = 6.0 Hz, 2H), 1.98 (s, 3H), 1.31 (d, J = 7.3 Hz, 3H), 1.28 (d, J = 7.3 Hz, 3H), 1.22 (d, J = 6.4 Hz, 3H). **$^{31}$P NMR** (242.9 MHz, $D_2O$) δ: -1.31 (s). **$^{13}$C NMR** (125.9 MHz, $D_2O$) δ: 175.50, 175.39, 174.50, 174.13, 172. 79, 171.80, 171.61, 171.21, 170. 95, 150.25, 148.09, 142.24, 118.64, 118.64, 87.97, 84.01, 83.95, 74.49, 72.02, 71.99, 70.19, 64.72, 64.68, 58.43, 58.38, 55.73, 49.94, 49.55, 42.52, 42.45, 42.34, 42.07, 25.16, 21.69, 17.92, 16.68, 16.19.

The threonine peptide Ac-CGAGTGAG-NH2 (**S3**) was synthesized on 100 mg (25 µmol) of Tentagel-S-RAM resin. Yield: 28% (4.4 mg, 6.9 µmol). Analytical **HPLC** $R_t$ = 2.449 min (A/B, (98 : 2) → (75 : 25), 500 µL/min, 7 min); Preparative HPLC $R_t$ = 7.148 min (A/B, (97.5 : 2.5) → (50 : 50), 20 mL/min, 10 min). **HR-ESI-MS**, m/z: 634.2625 ($[M+H]^+$, calc. 634.2613), 656.2450 ($[M+Na]^+$, calc. 656.2433). **$^1$H NMR** (600 MHz, $D_2O$) δ: 4.43 (t, J = 6.1 Hz, 1H), 4.29-4.18 (m, 4H), 3.94-3.88 (m, 6H), 3.81 (d, J = 5.7 Hz, 2H), 2.85 (d, J = 6.2 Hz, 2H), 1.98 (s, 3H), 1.33-1.30 (m, 6H), 1.12 (d, J = 6.4 Hz, 3H). **$^{13}$C NMR** (125.9 MHz, $D_2O$) δ: 175.60, 175.51, 174.52, 174.13, 172.81, 172.65, 171.86, 171.36, 171.12, 66.97, 59.14, 58.38, 55.73, 49.92, 49.80, 42.47, 42.45, 42.41, 42.07, 25.15, 21.69, 18.63, 16.47, 16.27.

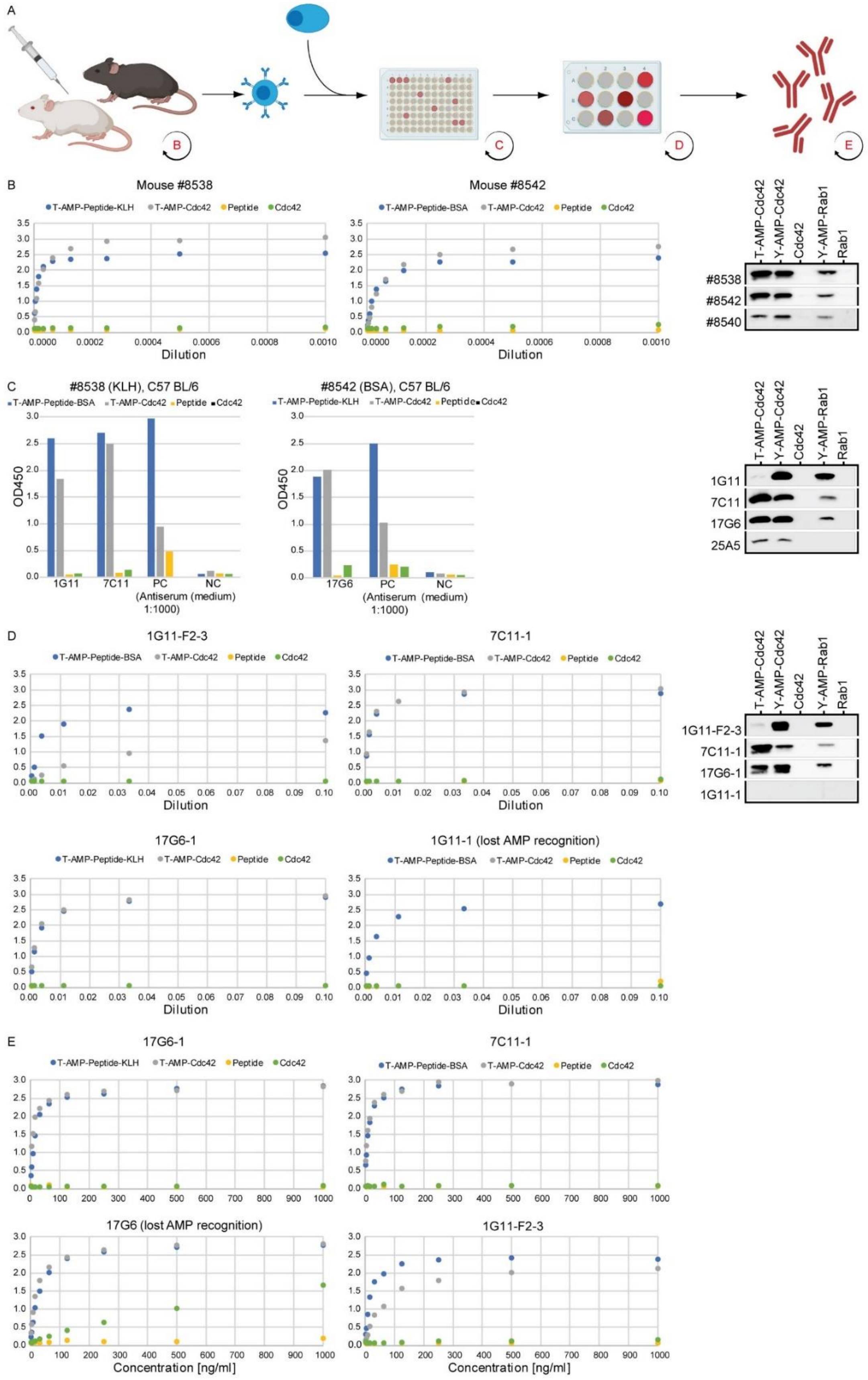


**Figure S3: Antibody generation and selection of antibodies binding to native epitopes.** Related to Figure 1E. **(A)** Immunization protocol of mice. Evaluation time points are indicated by circular arrows. **B-D** Evaluation regarding recognition of AMPylation in WB and ELISA of **(B)** mice sera **(C)** parental hybridoma clones **(D)** subclones and **(E)** produced and purified antibodies.

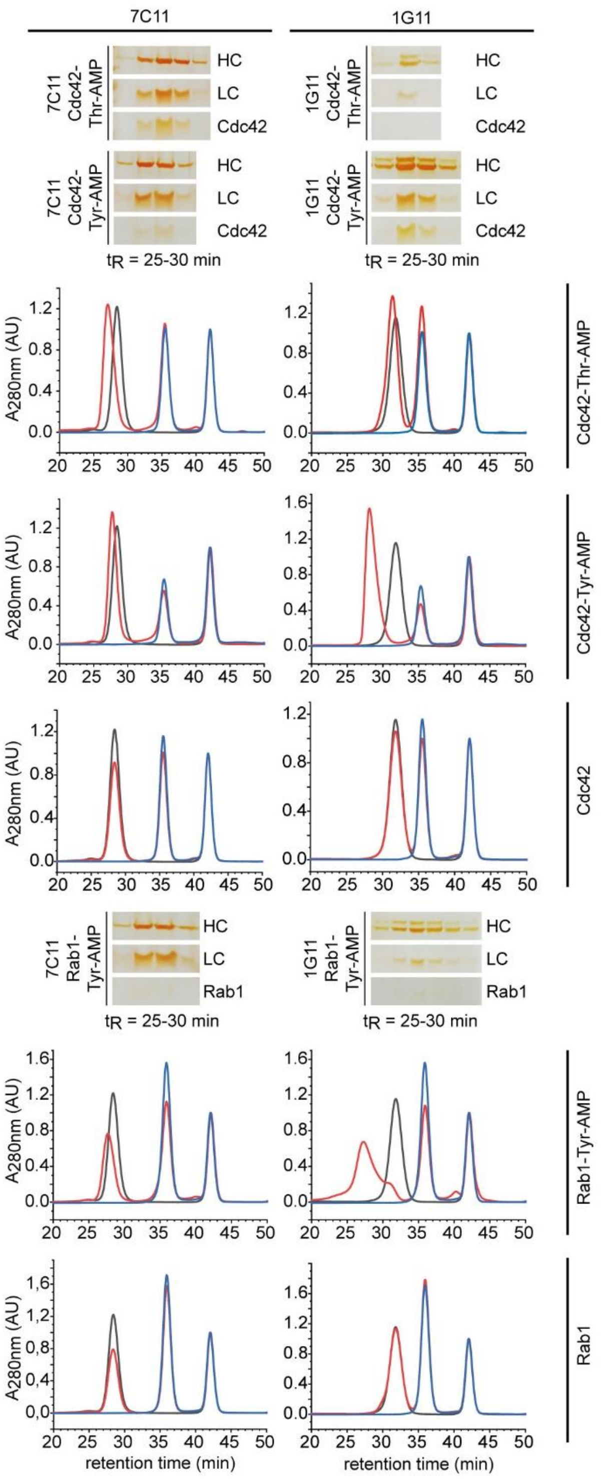


**Figure S4: Native binding of AMPylated antigens by monoclonal antibodies 7C11 and 1G11 as determined by analytical size exclusion chromatography.** Related to Figure 2C. In black antibody alone, in blue antigen alone as indicated, in red co-incubation of antibody and antigen as indicated. Shifted antibody peaks (red) upon co-incubation with AMPylated antigens were fractionated and analyzed for co-elution of antibody (light chain, LC; heavy chain, HC) and antigen by silver stained SDS PAGE (cropped rows represent identical gels).

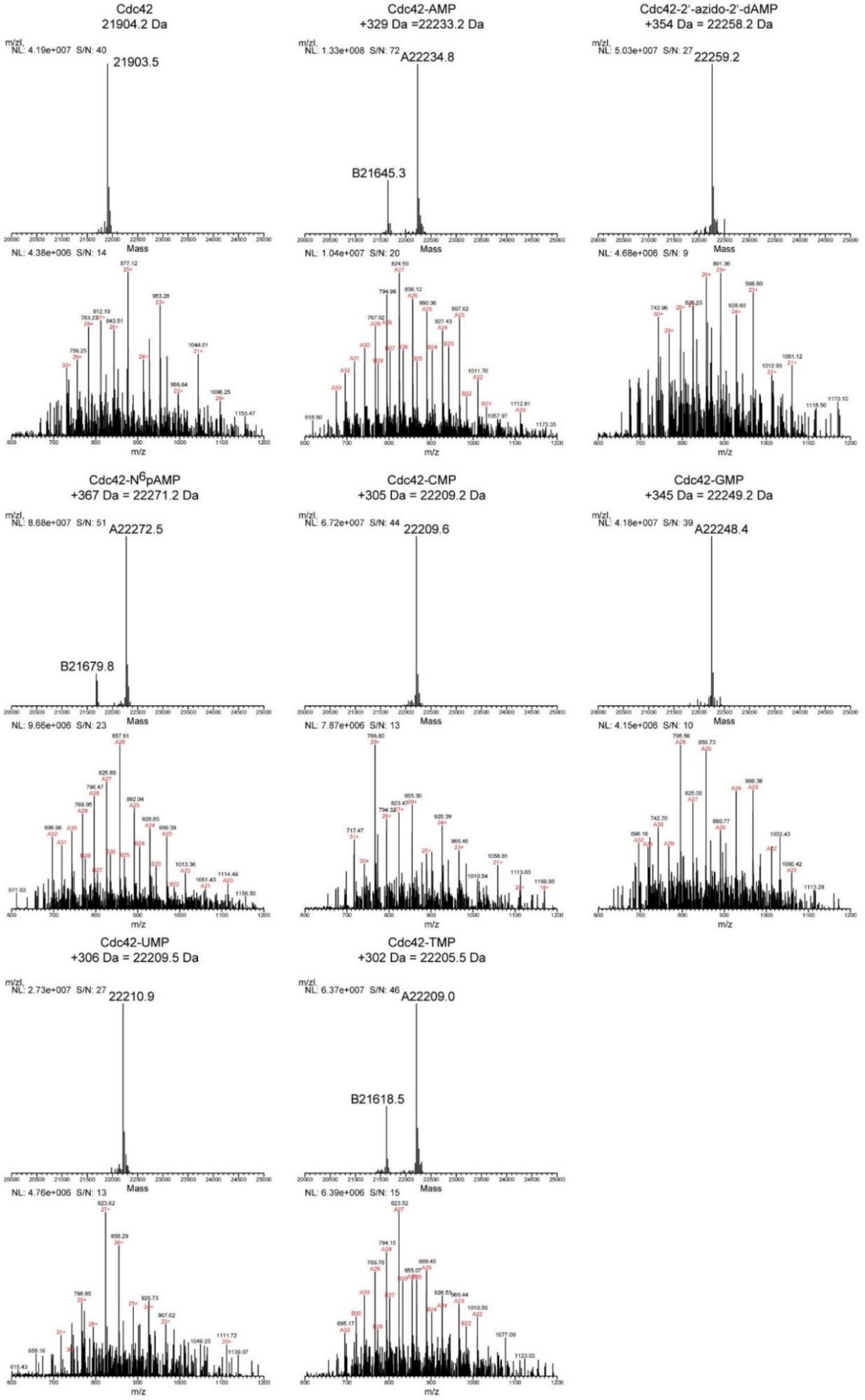


**Figure S5: Mass spectrometry confirmation of NMP incorporation by IbpA into Cdc42.** Related to Figure 2D. Expected molecular weights are indicated above spectra.

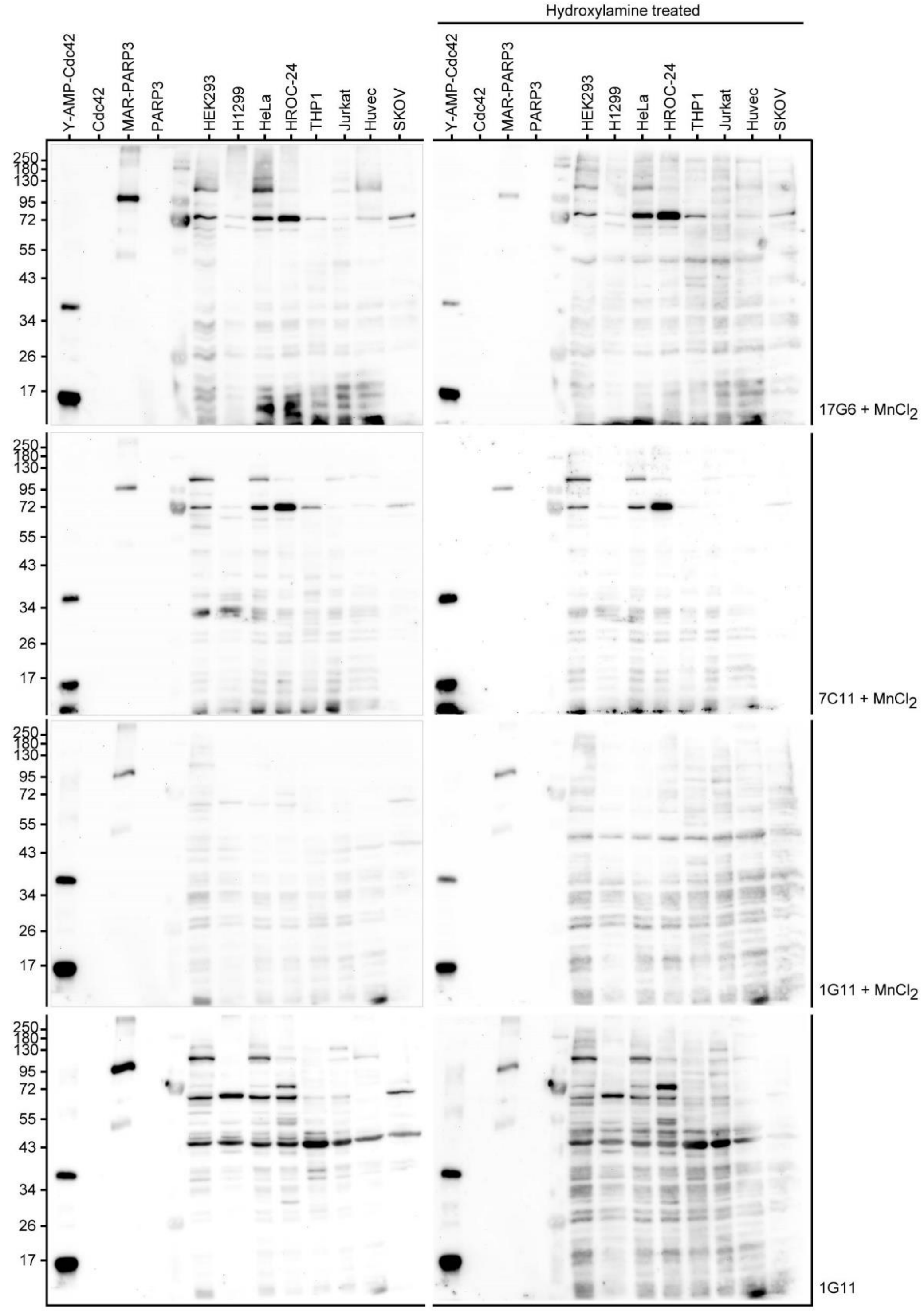


**Figure S6: Analysis of AMPylation patterns in various immortalized and cancer cell lines in WB.** Related to Figure 4F. 20 µg total protein cell lysate per lane of cell lines as indicated were blotted and probed with monoclonal anti-AMP antibodies as indicated using 1 mM $MnCl_2$ as additive during primary antibody incubation. Afterwards the identical blot was treated with 1 M hydroxylamine to cleave ADP-ribosylation at aspartate and glutamate residues and reprobed with monoclonal anti-AMP antibodies in the presence of 1 mM $MnCl_2$. In case of antibody 1G11, the identical blot was reprobed with 1G11 in the absence of $MnCl_2$ before and after hydroxylamine treatment. 10 ng recombinant Cdc42-Tyr-AMP serve as positive ctrl for AMPylation, 10 ng recombinant MAR-PARP3 as positive ctrl for ADP-ribosylation and successful hydroxylamine treatment. 10 ng unmodified counterparts are included as negative ctrl.

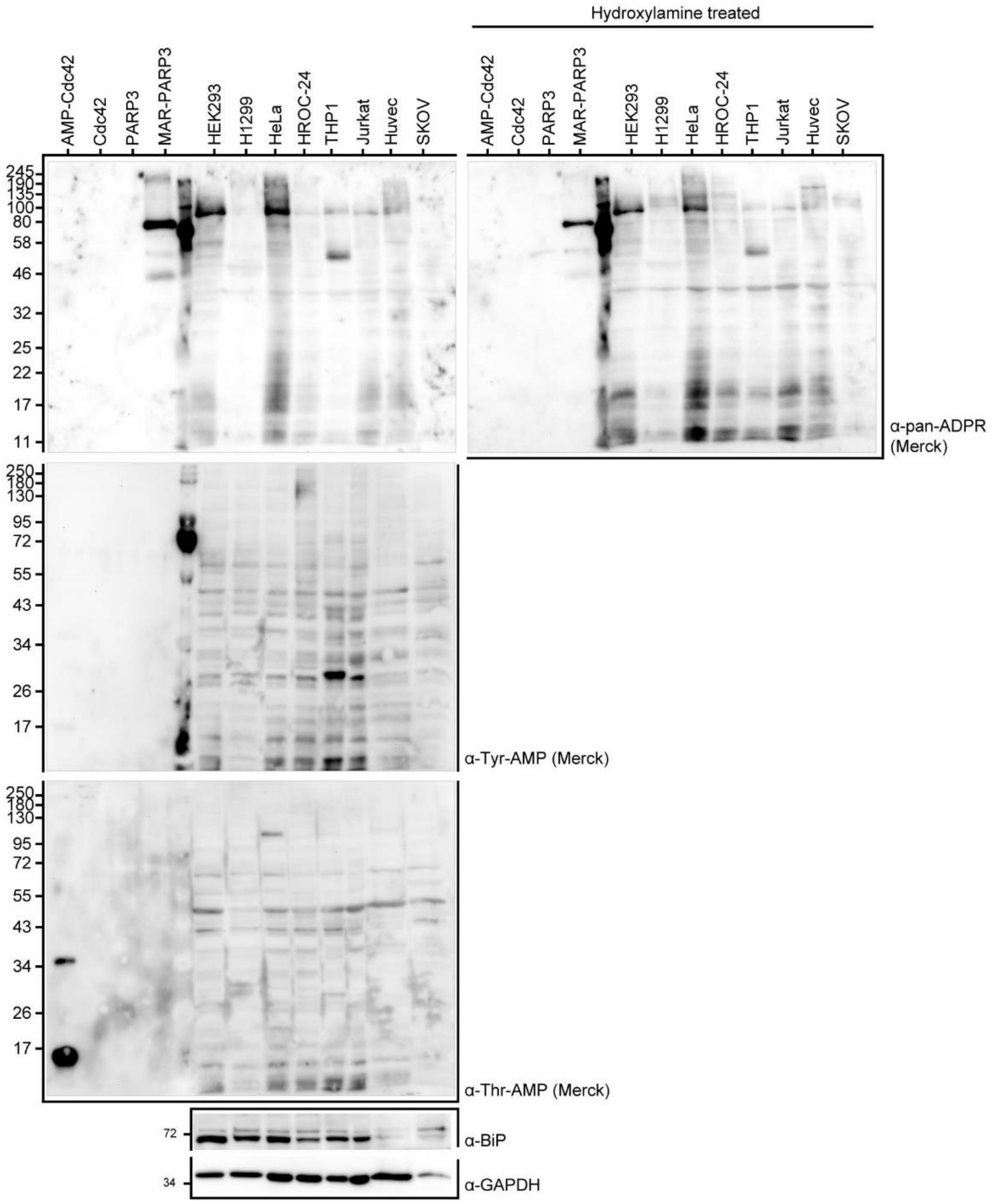


**Figure S7: Analysis of AMPylation patterns in various immortalized and cancer cell lines in WB.** Related to Figure 4F. 20 µg total protein RIPA cell lysate per lane of cell lines as indicated were blotted and probed with commercial anti-AMP antibodies as indicated as well as commercial anti-ADPR probe. For the anti-ADPR probe, the identical blot was afterwards treated with 1 M hydroxylamine to cleave ADP-ribosylation at aspartate and glutamate residues and reprobed with the anti-ADPR probe. The blot previously probed with commercial anti-Thr-AMP was stripped and reprobed with antibodies against BiP and GAPDH as loading control. 10 ng recombinant Cdc42-Tyr-AMP, or 10 ng recombinant Cdc42-Thr-AMP in case of anti-Thr-AMP antibody, serve as positive ctrl for AMPylation, 10 ng recombinant MAR-PARP3 as positive ctrl for ADP-ribosylation and successful hydroxylamine treatment. 10 ng unmodified counterparts are included as negative ctrl.

# Transparent Methods

**Table S1: Strategy for monoclonal anti-AMP antibody generation in mice.** Related to Figures 1 and S3. Experiments were conducted as indicated as a service by GenScript, Piscataway Township, New Jersey.

| Stage | Description | Shipment |
|---|---|---|
| Immunogen preparation | Peptide conjugation with KLH and BSA. | - |
| Phase I: Immunization | Group A: 3 BALB/c +2 C57 mice, immunization with KLH conjugates, Group B: 3 BALB/c +2 C57 mice, immunization with BSA conjugates, Immunization with conventional protocol, indirect ELISA primary screening with target peptide (AcNH-CGAGT(AMP)GAG-NH2), and confirmatory screening by indirect ELISA by target protein (Cdc42-AMP) | 15µl sera of 10 animals |
| Phase II: Cell fusion | 2 cell fusions, indirect ELISA primary screening with target peptide (AcNH-CGAGT(AMP)GAG-NH2), and confirmatory screening by indirect ELISA by target protein (Cdc42-AMP) | Supernatants of 10 clones,<br>2ml per clone |
| Phase III: Subcloning, Screening and Expansion | 3 cell lines subcloning, indirect ELISA primary screening with target peptide (AcNH-CGAGT(AMP)GAG-NH2), and confirmatory screening by indirect ELISA by target protein (Cdc42-AMP) | 2 vials and 5ml supernatant per subclone |
| Antibody production | 1 L roller bottle cell culture and protein A purification, indirect ELISA primary screening with target peptide (AcNH-CGAGT(AMP)GAG-NH2), and confirmatory screening by indirect ELISA by target protein (Cdc42-AMP) | >15mg purified antibody per subclone |

***Solid phase peptide synthesis (SPPS)***

The peptides S2 and S3 (Supplemental Figure S1 and S2) were synthesized on a MultiSyntech Syro I automated peptide synthesizer, using Tentagel Rink-amide resin as solid phase employing 8 equivalents of amino acid and 7.8 equivalents HBTU, 7.8 equivalents HOBt and 16 equivalents DIPEA in DMF. Threonine-AMP building block S1 (Smit et al., 2011) was manually coupled according to below. The resin was pre-swollen by treatment with DCM (15 min). Removal of Fmoc protecting group was performed by treatment with 2 times 3 minutes followed by 1 time 9 minutes 20 vol% piperidine in DMF. The resin was washed with DMF three times. The building block S1 (1.7 equivalents) was coupled using 1.6 equivalents HATU, 1.6 equivalents HOAt and 3.5 equivalents DIPEA in DMF. The coupling reaction was allowed to proceed for 4 h at room temperature. N-terminal acetyl-capping was achieved by adding 50 equivalents acetic anhydride and 50 equivalents DIPEA in NMP to the resin (1 h), followed by washing with DMF. Before cleavage, the resin was washed thoroughly with DCM (5 times), isopropanol (5 times) and diethyl ether (5 times). The peptides were cleaved with 5% TIPS and 5% water in TFA (1 x 2 h + 2 x 10 min). An additional 10% water was added to the combined TFA-fractions and allowed to age for one hour, in order to ensure complete hydrolysis of the acetonide moiety. The cleavage mixture was evaporated to dryness *in vacuo*. The resulting solid was dissolved in a minimum TFA and precipitated by the addition of 10 ml ice cold diethyl ether. The precipitate was dissolved in water/acetonitrile and subsequently lyophilized overnight. Pure peptides were obtained after purification by preparative HPLC. Preparative HPLC purifications of the peptides were performed with an Agilent 1260 Infinity series instrument equipped with a Phenomenex Luna (5 µm, C18(2) 100 Å, 250 x 21.2 mm) column. Used mobile phases were water with 0.1% TFA (eluent A) and acetonitrile with 0.1% TFA (eluent B). Analytical HRMS-HPLC was performed on an Agilent 1290 Infinity II series instrument equipped with an Agilent Extend (1.8 µm, C18, 100 Å, 50 x 2.1 mm) column and connected to an Agilent 6230 TOF LC/MS instrument. Used mobile phases were water with 0.1% FA (eluent A) and acetonitrile with 0.1% FA (eluent B). For complete description see supplemental data.

***Immunogen preparation***

Immunogen peptide S2 (ACNH-CGAGT(AMP)GAG-NH2) was conjugated with KLH and BSA as immunogen via its N-terminal cysteine (GenScript).

***Immunization***
3 BALB/c and 2 C57 BL/6 mice were immunized with either S2 conjugated to KLH (group A, BALB/c mice #8534 - #8536 and C57 BL/6 mice #8537 - #8538) or BSA (group B, BALB/c mice #8539 - #8541 and C57 BL/6 mice #8542 - #8543), respectively, according to the conventional protocol of GenScript (Table 2), resulting in 10 immunized mice in total.

**Table S2: Immunization schedule.** Related to Figures 1 and S3.

| Procedure | Schedule | Dosage and route | Adjuvant |
|---|---|---|---|
| Pre-Immune Bleed | T= - 4 days | | |
| Primary Immunization | T= 0 days | 50 µg /animal, i.p. | CFA |
| Boost 1 | T= 14 days | 25 µg /animal, i.p. | IFA |
| Test Bleed 1 | T= 21 days | | |
| Boost 2 | T= 28 days | 25 µg /animal, i.p. | IFA |
| Test Bleed 2 | T= 35 days | | |
| Final Boost | T= 50±7 days | 25 µg /animal, i.p. | IFA |
| Cell Fusion | 4 days after final boost | | |

Test bleed: 7 days after each boost immunization, immunized animal sera were tested by indirect ELISA and competitive ELISA for immune response by GenScript. Western blot evaluation of pre-sera and sera after 3rd immunization against 200 ng of purified protein/lane using a 1:1000 dilution was performed in-house as described. Animals #8538 (group A) and #8542 (group B), both C57 BL/6 mice, were selected for phase II.

After three boosts, the sera of immunized mice were evaluated for their ability to recognize the peptide hapten conjugate and native Cdc42-Thr-AMP in ELISA. Only positive hits were evaluated for their WB performance on various AMPylated proteins to check for backbone independent recognition and potential side chain bias. Of ten immunized mice, five of these were able to recognize native Cdc42-Thr-AMP in ELISA as good as the AMPylated hapten without showing binding of Cdc42 alone. All of them were positive against all tested proteins in WB, although no preference for AMPylation at threonine side chains was observable and all sera reacted with tyrosine as well as threonine modifications. After the final bleed, the two positive animals #8538 and #8542, both C57 BL/6 mice, with superior recognition of native and denatured targets in WB and ELISA and no discernible background against unmodified proteins and peptides were selected to perform cell fusion to hybridoma cells.

***Phase II: cell fusion and screening***
For cell fusion and clone plating, two fusions were performed by electro-fusion. The average fusion efficiency at GenScript is around 1 hybridoma/2,000 splenocytes, thus the anticipated hybridoma clones would be ~ $2x10^4$. All fused cells from each cell fusion were plated into 96-well plates. Up to 15 plates were used for each fusion. For the primary binder screening, the conditioned medium was screened by ELISA with the target peptide. In the confirmatory screening, the conditioned medium of primary binder screening positive clones were screened against the positive screening material (Cdc42-Thr-AMP) and counter screening material (Cdc42). The expected clones should be positive against target peptides, positive screening material while negative against the negative peptide and counter screening material. For clone expansion and freezing, 10 positive clones were expanded into 24-well plates. 2 ml of supernatant (conditioned media) were collected for each clone and cells were frozen down to avoid clone loss (GenScript). The conditioned media of all 10 positive clones were analyzed in-house by WB against 200 ng of purified protein/lane using a 1:10 dilution as described. Clones 17G6 (#8542), 1G11 and 7C11 (#8538) were selected for phase III.

Evaluation using ELISA resulted in 10 clones that were able to recognize native Cdc42-Thr-AMP and were subsequently chosen for WB testing as described above. The variation of performance among the clones proved to be a lot higher than between mice sera samples, with many clones showing a lack of universal recognition of all targets, high background or strong differences in strength of recognition depending on the AMPylated protein. Two promising clones, 7C11 and 17G6, with similar very good recognition of all AMPylated proteins in WB independent of their modified side chain, native recognition of Cdc42-Thr-AMP in ELISA and low background were selected for subcloning and

subsequent production and purification. One further clone, 1G11, was selected for its unexpected development of a tyrosine-specific recognition, despite immunization with a threonine-modified peptide.

***Phase III: subcloning, screening, expansion.***
For subcloning, 3 positive primary clones were sub-cloned by limiting dilution to ensure that the sub-clones were derived from a single parental cell. The clones were carried for a maximum of 3 generations. Subcloning was screened by ELISA as before. For monoclone cryopreservation, two stable sub-clonal cell lines of each parental clone were chosen for cryopreservation based on the result of ELISA (GenScript). Positive cell supernatants were evaluated by WB against 200 ng of purified protein/lane using a 1:10 dilution in-house as described. The stable sub-clonal cell lines 17G6-1 (isotype IgG2b, k), 1G11-F3-3 (isotype IgG2b, k) and 7C11-1 (isotype IgG2a, k) were chosen for production and isotyped, and the cell lines stored with 2 vials at GenScript and 2 vials in-house. They were negatively tested for mycoplasma, detected by the PCR Mycoplasma Detection Set (TAKARA BIO INC, Kusatsu, Japan).

In our experience, subcloning and upscaling for antibody production poses the risk of losing binding abilities. One clone, 1G11-1, lost its AMPylation recognition abilities during subcloning and had to be recloned (1G11-F2-3) (Switch in isotypes from IgG1 to IgG2b). Another clone, 17G6-1, lost its performance during upscaling for antibody production and had to be redone. Therefore, rigorous retesting after each step is crucial. Interestingly, clone 1G11 lost its clear preference for tyrosine AMPylation after upscaling for production (Figure 2A). However, tyrosine-specific recognition of antibody 1G11 could be sharply enhanced in the presence of 1 mM $MnCl_2$ (Figure 3B).

For all three final hybridoma cell lines 17G6-1, 7C11-1 and 1G11-F2-3, antibody variable domain sequencing was done by GenScript as a service.

***Antibody variable domain sequencing of hybridoma cell lines***
Total RNA was isolated from frozen hybridoma cell lysates following the technical manual of TRIzol® Reagent (Ambion, Thermo Fisher Scientific Inc.). Total RNA was then reverse transcribed into cDNA using isotype-specific anti-sense primers or universal primers following the technical manual of PrimeScript™ 1st Strand cDNA Synthesis Kit (Takara, Kusatsu, Japan). The antibody fragments of $V_H$ and $V_L$ were amplified according to the standard operating procedure (SOP) of rapid amplification of cDNA ends (RACE) of GenScript. Amplified antibody fragments were cloned into a standard cloning vector separately. Colony PCR was performed to screen for clones with inserts of correct sizes. No less than five colonies with inserts of correct sizes were sequenced for each fragment. The sequences of different clones were aligned and the consensus sequences of these clones were provided (GenScript).

***17G6-1 antibody variable domain sequencing***
**Heavy chain: DNA sequence (411 bp)**

Leader sequence-FR1-CDR1-FR2-CDR2-FR3-CDR3-FR4

ATGGGATGGAGCTGGATCTTTCTCTTTCTCCTGTCAGGAACTGCAGGTGTCCTCTCTGAGGTCCA
GCTGCAACAATCTGGACCTGAGCTGGTGAAGCCTGGGGCTTCAGTGAAGATATCCTGTAAGGCT
TCTGGATACACGTTCACTGACTACTATCTGAACTGGGTGAAGCAGAGCCATGGAAAGAGCCTTGA
GTGGATTGGAGATATTCATCCTAACAAAGGTATTAATAAGTACAACCAGAAGTTCAAGGGCAAGG
CCACATTGACTGTAGACAAGTCCTCCAGTACAGCCTACATGGAGCTCCGCAGCCTGACATCTGAG
GACTCTGCAGTCTATTACTGTGCAAGATACTACGGTAAGAGGTACTTCGATGTCTGGGGCACAGG
GACCATGGTCACCGTCTCCTCA

**Heavy chain: Amino acid sequence (137 aa)**

Leader sequence-FR1-CDR1-FR2-CDR2-FR3-CDR3-FR4

MGWSWIFLFLLSGTAGVLSEVQLQQSGPELVKPGASVKISCKASGYTFTDYYLNWVKQSHGKSLEWI
GDIHPNKGINKYNQKFKGKATLTVDKSSSTAYMELRSLTSEDSAVYYCARYYGKRYFDVWGTGTMVT
VSS

**Light chain: DNA sequence (396 bp)**

Leader sequence-FR1-CDR1-FR2-CDR2-FR3-CDR3-FR4

ATGATGAGTCCTGCCCAGTTCCTGTTTCTGTTAGTGCTCTGGATTCGGGAAACCAACGGTGATGT
TGTGATGACCCAGACTCCACTCACTTTGTCGGTTACCCTTGGACAACCAGCCTCCATCTCTTGCA
AGTCACGTCAGAGCCTCTTAGATAGTGATGGAAAGACATATTTGAATTGGTTGTTACAGAGGCCA
GGCCAGTCTCCAAAGCGCCTAATCTATCTGGTGTCTAAATTGGACTCTGGAGTCCCTGACAGGTT

CACTGGCAGTGGATCAGGGACAGATTTCACACTGAAAATCAGCAGAGTGGAGGCTGAGGATTTG
GGAGTTTACTATTGCTGGCAAGGTACACATTTTCCGTACACGTTCGGAGGGGGGACCAAGCTGG
AAATAAAA

**Light chain: Amino acid sequence (132 aa)**

Leader sequence-FR1-CDR1-FR2-CDR2-FR3-CDR3-FR4

MMSPAQFLFLLVLWIRETNGDVVMTQTPLTLSVTLGQPASISCKSRQSLLDSDGKTYLNWLLQRPGQ
SPKRLIYLVSKLDSGVPDRFTGSGSGTDFTLKISRVEAEDLGVYYCWQGTHFPYTFGGGTKLEIK

***7C11-1 antibody variable domain sequencing***
**Heavy chain: DNA sequence (417 bp)**

Leader sequence-FR1-CDR1-FR2-CDR2-FR3-CDR3-FR4

ATGGGATGGAGCTGGATCTTTCTCTTTCTCCTGTCAGGAACTGCACGTGTCCTCTCTGAGGTCCA
GCTGCAACAATCTGGACCTGAGCTGGTGAAGCCTGGGGCTTCAGTGAAGATATCCTGTAAGGCT
TCTGGATACACGTTCACTGACTACTACATTAACTGGGTGAAGCAGAGCCATGGAAAGAGCCTTGA
GTGGATTGGAGATATTAATCCTAACCATGGTAGTGCTGGCTCCAACCAGAATTTCCAGGGCAAGG
CCACATTGACTGTAGACAAGTCCTCCAGCACAGCCTACATGGAGCTCCGCAGCCTGACATCTGA
GGACTCTGCAGTCTATTACTGTACAAAGTACTACGGTACTGGCTACAGGTACTTCGATGTCTGGG
GCACAGGGACCACGGTCACCGTCTCCTCA

**Heavy chain: Amino acid sequence (139 aa)**

Leader sequence-FR1-CDR1-FR2-CDR2-FR3-CDR3-FR4

MGWSWIFLFLLSGTARVLSEVQLQQSGPELVKPGASVKISCKASGYTFTDYYINWVKQSHGKSLEWI
GDINPNHGSAGSNQNFQGKATLTVDKSSSTAYMELRSLTSEDSAVYYCTKYYGTGYRYFDVWGTGT
TVTVSS

**Light chain: DNA sequence (396 bp)**

Leader sequence-FR1-CDR1-FR2-CDR2-FR3-CDR3-FR4

ATGATGAGTCCTGCCCAGTTCCTGTTTCTGTTAGTGCTCTGGATTCGGGAAACCAACGGTGATGT
TGTGATGACCCAGACTCCACTCACTTTGTCGGTTACCATTGGACAACCAGCCTCCATCTCTTGCA
AGTCAAGGCTGAGCCTCTTAGAAAGTGATGGAAAGACATATTTGAATTGGTTGTTACAGAGACCA
GGCCAGTCTCCAAAGCGCCTAATCTATCAGGTGTCTAAGCTGGACTCTGGAGTCCCTGACAGGTT
CACTGGCAGTGGATCAGGGACAGATTTCACACTGAAAATCAGCAGAGTGGAGGCTGAGGATTTG
GGAGTTTATTATTGCTGGCAAGGTACACATTTTCCGTACACGTTCGGAGGGGGGACCAAGCTGG
AAATAAAA

**Light chain: Amino acid sequence (132 aa)**

Leader sequence-FR1-CDR1-FR2-CDR2-FR3-CDR3-FR4

MMSPAQFLFLLVLWIRETNGDVVMTQTPLTLSVTIGQPASISCKSRLSLLESDGKTYLNWLLQRPGQS
PKRLIYQVSKLDSGVPDRFTGSGSGTDFTLKISRVEAEDLGVYYCWQGTHFPYTFGGGTKLEIK

***1G11-F2-3 antibody variable domain sequencing***
**Heavy chain: DNA sequence (423 bp)**

Signal sequence-FR1-CDR1-FR2-CDR2-FR3-CDR3-FR4

ATGGGTTGGCTGTGGACCTTGCTATTCCTGATGACAGCTGCCCAAAGTGCCCAAGCACAGATCC
AGTTGGTACAGTCTGGACCTGAACTGAAGAAGCCTGGAGAGACAGTCAAGATCTCCTGCAAGGC
TGCTGGGTATACCTTCACAACCTATGGAATGAGCTGGGTGAAACAGGCTCCAGGAAAGGGTTTAA
AGTGGCTGGGCTGGATAAACACCTACTCTGGAGTGCCAACATATGCTGATGACTTCAAGGGACG
GTTTGCCTTCTCTTTGGATATCTCTGCCAGCACTGCCTATTTGCAGATCAACAACCTCATAAATGA
GGACACGGCTGCATATTTCTGTACAAGGAGGGACCGGTACTATCCTAACTACGACTACTTTGACT
ACTGGGGCCAAGGCACCACTCTCACAGTCTCCTCA

**Heavy chain: Amino acid sequence (141 aa)**

Signal peptide-FR1-CDR1-FR2-CDR2-FR3-CDR3-FR4

MGWLWTLLFLMTAAQSAQAQIQLVQSGPELKKPGETVKISCKAAGYTFTTYGMSWVKQAPGKGLKW
LGWINTYSGVPTYADDFKGRFAFSLDISASTAYLQINNLINEDTAAYFCTRRDRYYPNYDYFDYWGQG
TTLTVSS

**Light chain: DNA sequence (393 bp)**

Signal sequence-FR1-CDR1-FR2-CDR2-FR3-CDR3-FR4

ATGAAGTTGCCTGTTAGGCTGTTGGTGCTGATGTTCTGGATTCCTGCTTCCACCAGTGATGTTTTG
TTGACCCAAACTCCACTCTCCCTGCCTGTCAGTCTTGGAGATCAAGCCTCCATCTCTTGCAGATC
TAGTCAGAGCCTTGTACATAGTACTGGAAACACCTATTTAGAATGGTACCTGCAGAAACCAGGCC
AGTCTCCAAAACTCCTGATCTACAAAGTTTCCACCCGATTTTCTGGGGTCCCAGACAGGTTCAGT
GGTAGTGGATCAGGGACAGATTTCATACTCAAGATCAGCAGAGTGGAGGCTGAGGATCTGGGAG
TTTATTACTGCTTTCAAGGTTCACATGGACCGCTCACGTTCGGTGCTGGGACCAAGCTGGAGCTG
AAA

**Light chain: Amino acid sequence (131 aa)**

Signal peptide-FR1-CDR1-FR2-CDR2-FR3-CDR3-FR4

MKLPVRLLVLMFWIPASTSDVLLTQTPLSLPVSLGDQASISCRSSQSLVHSTGNTYLEWYLQKPGQSP
KLLIYKVSTRFSGVPDRFSGSGSGTDFILKISRVEAEDLGVYYCFQGSHGPLTFGAGTKLELK

***Antibody production***
Three stable sub-clonal cell lines were each cultured in 1 l roller bottle cell culture using SFM + 2% low IgG FBS culture medium. Monoclonal antibody was Protein A purified from the supernatant and stored in phosphate buffered saline (PBS, pH 7.4) with 0.02% sodium azide as preservative. Purity was measured by SDS-PAGE and concentration by NanoDrop Spectrophotometer A280nm (Thermo Fisher Scientific Inc., Waltham, Massachusetts) (GenScript). This way 36.38 mg of 7C11 with 95% purity, 17.10 mg 17G6 with 91% purity and 30.99 mg 1G11 with 92% purity were produced.

***Molecular biology***
Unless otherwise indicated, all genes were codon optimized for expression in *E. coli* by omitting rare amino acid codons, and all cloning was done by sequence and ligation independent cloning (SLIC) using T4 DNA polymerase (New England Biolabs, Ipswich, Massachusetts).

The human Cdc42 1-179aa Q61L (referred to as Cdc42)-encoding DNA was cloned into a modified pGEX-4T-1 vector (GE Healthcare, Chicago, Illinois) as previously described (Barthelmes et al., 2020), resulting in a construct with a N-terminal glutathione S-transferase (GST) tag followed by the Tobacco Etch Virus (TEV) protease cleavage site. As previously described (Schoebel et al., 2009), the human Rab1b 3-174aa (referred to as Rab1b)-encoding DNA was cloned into a modified pMAL vector (New England Biolabs), resulting in a construct with a N-terminal hexahistidine (6xHis) tag, followed by maltose-binding protein (MBP) and the TEV protease cleavage site. The human BiP 19-654aa (referred to as BiP)-encoding DNA was cloned into a modified pProEx™-HTb vector (Thermo Fisher Scientific Inc.), resulting in a construct with a N-terminal 6xHis tag, followed by the TEV protease cleavage site. The human FICD 102-458aa E234G (referred to as FICD)-encoding DNA was cloned into a modified pMAL vector (New England Biolabs), resulting in a construct with a N-terminal 6xHis tag, followed by the HaloTag®, the TEV protease cleavage site and a Strep-tag® II. The *Vibrio parahaemolyticus* VopS 31-387aa (referred to as VopS)-encoding DNA was cloned into a modified pMAL vector (New England Biolabs) as previously described (Barthelmes et al., 2020), resulting in a construct with a N-terminal 6xHis tag, followed by MBP and the TEV protease cleavage site. The *Histophilus somni* IbpA 3483-3797aa I3455C (referred to as IbpA)-encoding DNA was cloned into a modified pSF vector (Oxford Genetics Ltd, Oxford, UK), resulting in a construct with a N-terminal decahistidine (10xHis) tag, followed by MBP, the TEV protease cleavage site and a 3xFLAG® tag. The *Legionella pneumophila* DrrA 8-533aa (referred to as DrrA)-encoding DNA was cloned into a modified pET19 vector (Merck Millipore, Burlington, Massachusetts) as previously described (Müller et al., 2010), resulting in a construct with a N-terminal 6xHis tag and the TEV protease cleavage site. The *Legionella pneumophila* AnkX 1-800aa (referred to as AnkX)-encoding DNA, which previously had been amplified from *Legionella pneumophila* genomic DNA (Goody et al., 2012), was cloned into a modified pSF vector (Oxford Genetics) as previously described (Ernst et al., 2020), resulting in a construct with a N-terminal 10xHis tag, followed by enhanced green fluorescent protein (eGFP) and the TEV protease cleavage site. Human Rab8a 6-176aa (referred to as Rab8a)-encoding DNA was cloned into a pet51b(+) vector (Merck Millipore), resulting in a construct with a N-terminal Strep® II tag and enterokinase cleavage site and a C-terminal 10xHis tag. All site-specific mutagenesis was performed with the Q5 Site-Directed Mutagenesis Kit (New England Biolabs).

***Recombinant expression and purification of proteins***
Recombinant human histone H3.1 was purchased from New England Biolabs (M2503S), and active human PARP3 from Sigma-Aldrich, St. Louis, Missouri (SRP0194-10UG, Lot #8050330111). Human pSer111 Rab1b was a kind gift of Dr. Sophie Vieweg and was produced as published before (Vieweg et al., 2020).

Cdc42, VopS, Rab1b, DrrA and AnkX were expressed and purified as previously described (Barthelmes et al., 2020; Ernst et al., 2020; Müller et al., 2010; Schoebel et al., 2009).
In brief, plasmids were transformed into chemically competent BL21 (DE3) cells (Cdc42, Rab1b, IbpA) or Lemo21 cells (VopS) or BL21-CodonPlus (DE3)-RIL cells (DrrA, Rab8a, AnkX) or Rosetta 2 cells (BiP, FICD) and protein was expressed in LB medium after induction with 0.5 mM isopropyl-β-dithiogalactopyranoside (IPTG) for 20 h at 25 °C (Cdc42, Rab1b) or 20 °C (VopS, DrrA, Rab8a, AnkX, IbpA) or 23 °C (FICD) or 3 h at 37 °C (BiP). Cells were harvested, washed with PBS and lysed in 50 mM 4-(2-hydroxyethyl)-1-piperazineethanesulfonic acid (Hepes) pH 7.5, 500 mM sodium chloride (NaCl), 1 mM $MgCl_2$, 2 mM ß-mercaptoethanol (ßMe), 10 µM guanosine diphosphate (GDP), 1 mM PMSF (Cdc42, Rab1b, Rab8a) or 50 mM Tris pH 8.0, 500 mM NaCl, 5% (v/v) glycerol, 2 mM β-Me, 1 mM PMSF (AnkX) or 50mM Hepes pH 7.5, 500 mM NaCl, 1 mM $MgCl_2$, 2mM ßMe, 1 mM PMSF (VopS, FICD) or 50 mM Hepes pH 8.0, 500 mM lithium chloride (LiCl), 2 mM β-Me, 1 mM PMSF (DrrA, IbpA) or 50 mM Hepes pH 7.4, 400 mM NaCl, 20 mM imidazole, 1 mM PMSF (BiP) after addition of DNase I by French press at 1.8 kbar. The lysates were cleared by centrifugation.
For GST-tagged proteins (Cdc42), the lysate was loaded onto a pre-equilibrated GST-Trap column (GE) and eluted with 3-5 column volumes (CV) of the same buffer supplemented with 10 mM glutathione.
For His-tagged proteins (VopS, Rab1b, DrrA, BiP, FICD, Rab8a, IbpA), the lysate was loaded onto a pre-equilibrated $Ni^{2+}$-charged Bio-Scale Mini Nuvia IMAC Cartridge (Bio-Rad Laboratories, Hercules, California), washed with 30 mM imidazole and eluted with a fractioned gradient from 30 mM – 350 mM imidazole over 20 CV.

The protein containing eluate was digested with 6x-His tagged TEV during dialysis against 50 mM Hepes pH 7.5, 100 mM NaCl, 2 mM ßMe, 10 µM GDP (Cdc42, Rab1b) or 20 mM Hepes pH 8.0, 100 mM NaCl, 2 mM β-Me (DrrA, IpbA) or 20 mM Tris pH 8.0, 300 mM NaCl, 5% (v/v) glycerol, 2 mM β-Me (AnkX) 20 mM Hepes pH 7.4, 100 mM NaCl (BiP) or 20 mM Hepes pH 7.4, 100 mM NaCl, 1 mM $MgCl_2$, 1 mM ßMe (FICD) with a cut off of MW 6000-8000 (Serva) for 16h at 4°C.
Digested, formerly His-tagged proteins (Rab1b, DrrA, BiP, FICD, AnkX, IbpA) were reapplied to the $Ni^{2+}$-charged column pre-equilibrated with dialysis buffer in order to remove the His-tag, uncleaved protein and TEV protease. The flow through was collected, concentrated and applied to a HiLoad™ 16/600 Superdex™ 75pg column (GE Healthcare) in 20 mM Hepes pH 7.5, 50 mM NaCl, 1 mM $MgCl_2$, 1 mM dithioerythritol (DTE), 10 µM GDP (Rab1b) or 20 mM Hepes pH 8.0, 100 mM NaCl, 2 mM DTE (DrrA) or 20 mM Tris pH 8.0, 300 mM NaCl, 5% (v/v) glycerol, 1 mM β-Me (AnkX) or 20 mM Hepes pH 7.4, 150 mM potassium chloride (KCl), 1 mM $MgCl_2$, 1 mM tris(2-carboxyethyl)phosphine (TCEP), 5% glycerol (FICD) or 20 mM Hepes pH 8.0, 100 mM NaCl, 1 mM $MgCl_2$, 2 mM DTE (IbpA) or applied to a HiLoad™ 16/600 Superdex™ 200pg column (GE Healthcare) in 20 mM Hepes pH 7.4, 150 mM KCl, 10 mM $MgCl_2$ (BiP). For VopS and Rab8a, protein containing eluate was concentrated and applied to a HiLoad™ 16/600 Superdex™ 75pg column (GE Healthcare) in 20 mM Hepes pH 7.5, 100 mM NaCl, 1 mM $MgCl_2$, 1 mM DTT (VopS) or 20 mM Hepes pH 7.5, 50 mM NaCl, 1 mM $MgCl_2$, 1 mM DTE, 10 µM GDP (Rab8a) without TEV digestion. For digested, formerly GST-tagged proteins (Cdc42), protein digestion was concentrated and applied to a HiLoad™ 16/600 Superdex™ 75pg column (GE Healthcare) in 20 mM Hepes pH 7.5, 50 mM NaCl, 1 mM $MgCl_2$, 1 mM DTE, 10 µM GDP (Cdc42). During all steps of protein purification, fractions were collected and analyzed by Coomassie blue stained sodium dodecyl sulfate (SDS) polyacrylamide gel electrophoresis (PAGE). Fractions containing pure protein of interest were pooled, concentrated to around 10 mg/ml, snap-frozen in liquid nitrogen and stored in multiple aliquots at -80 °C.

***In vitro* AMPylation of recombinant proteins**

For Cdc42-Thr-AMP, 200 µM Cdc42 were incubated with 10 µM VopS in the presence of 800 µM ATP in 20 mM Hepes pH 7.5, 100 mM NaCl, 1 mM $MgCl_2$, 1 mM DTE at 20 °C overnight. For Cdc42-Tyr-AMP, 10 µM Cdc42 were incubated with 0.1 µM IbpA in the presence of 1 mM ATP in 20 mM HEPES pH 7.4, 100 mM NaCl, 1 mM MgCl2, 1 mM TCEP, 10 µM GDP at 20 °C overnight. For Rab1-Tyr-AMP, 10 µM Rab1b were incubated in the presence of 50 µM ATP and 0.1 µM DrrA at 25°C as previously described (Müller et al., 2010). AMPylated Cdc42 and Rab1b were purified by size exclusion chromatography on a HiLoad™ 16/600 Superdex™ 75pg column (GE Healthcare) in 20 mM Hepes pH 7.5, 50 mM NaCl, 1 mM $MgCl_2$, 1 mM DTE, 10 µM GDP and full AMPylation was confirmed by MS. For H3-Thr-AMP and auto-AMPylated FICD, 30 µM H3.1 were incubated with 25 µM FICD in the presence of 10 mM ATP in 20 mM HEPES pH 7.5, 150 mM NaCl, 1 mM MgCl2, 2 mM DTT at 20 °C overnight. For BiP-Thr-AMP, 50 µM BiP were incubated with 2.5 µM FICD in the presence of 1.5 mM ATP in 25 mM Hepes pH 7.4, 100 mM KCl, 4 mM $MgCl_2$, 1 mM calcium chloride ($CaCl_2$) for 2 h at 30 °C. BiP-AMP was purified with Protino™ Ni-NTA Agarose (Macherey-Nagel, Düren, Germany) in previously listed buffers according to the manufacturer's instructions.

***In vitro* NMPylation of recombinant Cdc42 by IbpA**

10 µM Cdc42 were incubated with 0.1 µM IbpA in the presence of 0.5 mM of either CTP, UTP, TTP, GTP, N6pATP (Jena Bioscience, Jena, Germany) and 2'-Azido-2'-dATP (TriLink BioTechnologies, San Diego, California) in 20 mM Tris-HCl pH 7.4, 100 mM NaCl, 1 mM $MgCl_2$, 1 mM TCEP, 10 µM GDP at 20 °C overnight. Successful NMPylation was confirmed by MS.

***In vitro* auto-mono-ADP-ribosylation of PARP3**

500 ng PARP-3 (Sigma-Aldrich) were incubated at 25°C in a 100 µL reaction volume in 20 mM HEPES pH 8.0, 5 mM MgCl2, 5 mM $CaCl_2$, 0.01% NP-40, 25 mM KCl, 1 mM DTT, 0.1 mg/mL salmon sperm DNA (Thermo Fisher Scientific), 0.1 mg/mL BSA (New England Biolabs) in the presence of 250 µM NAD+ for 30 minutes as published before (Gibson et al., 2017). The reaction was stopped by the addition of 5x SDS-PAGE Loading Buffer, followed by heating to 95°C for 5 min.

***In vitro* phosphocholination or Rab1b by AnkX**

10 µM Rab1b was incubated with 0.1 µM AnkX in the presence of 1 mM CDP-choline (Enzo Life Sciences, Farmingdale, New York) for 2 h at 23 °C as published before (Goody et al., 2012).

***In vitro* biotinylation of Rab8a**

200 mM EZ-Link® Maleimide-PEG2-Biotin (Thermo Fisher Scientific) stock solution in DMSO was diluted 1:10 in 1x PBS to a final concentration of 20 mM Maleimide-PEG2-Biotin label. 200 µM Maleimide-PEG2-Biotin label were added to 100 µM of Rab8a in PBS for 2 h on ice, before Rab8a was washed 3 times with PBS in an Amicon filter (Merck Millipore, 10 kDa NMWL). Incorporation of label was confirmed by MS.

### *Analytical size exclusion chromatography (aSEC)*

In 100 µl, 40 µg antigen were mixed with 60 µg antibody, including 50 µM Vitamin B12 as internal standard. 90 µl sample were injected onto a Superdeep 10/300 200pg column (GE Healthcare) coupled to a Prominence HPLC system (Shimadzu, Kyōto, Japan) and run at 0.5 ml/min for 60 min in 20 mM HEPES pH 7.5, 150 mM NaCl. Protein retention times were detected at 280 nm (A280nm), and intensities were normed to Vitamin B12. Peaks containing antigen:antibody complexes were collected in 500 µl fractions. Fractions were supplemented with 6x Laemmli and concentrated in a SpeedVac alpha RVC (Martin Christ Gefriertrocknungsanlagen GmbH, Osterode am Harz, Germany) without heat to 200 µl. 10 µl concentrated fractions were analyzed by 15% SDS PAGE and silver stained.

### *Mass spectrometry*

To verify the degree of modification, samples containing 100 ng recombinant protein were run over a 5 µm Jupiter C4 300Å LC column (Phenomenex, Torrance, California) using the 1260 Infinity LC system (Agilent Technologies, Santa Clara, California) and then subjected to mass spectrometry with the 6100 Quadrupole LC/MS System (Agilent Technologies). The resulting ion spectra were deconvoluted using the Magic Transformer (MagTran) software (Zhang and Marshall, 1998).

### *Cell culture*

CHO-K1 FlpIn cells (Thermo Fisher Scientific) were cultured in RPMI-1640 medium (Sigma-Aldrich) supplemented with 10% FBS (Thermo Fisher Scientific). HEK293 cells were cultured as described below. 90% confluent cells were stimulated by either 0.5 µM thapsigargin (Biosynth Carbosynth) for 2 h or 100 µg/ml cycloheximide (Sigma-Aldrich) for 4 h. Cells were washed twice with Dulbecco's Phosphate Buffered Saline (DPBS) (Sigma-Aldrich) and lysed in MPER buffer (Thermo Fisher Scientific) supplemented with cOmplete EDTA free protease inhibitor (Roche, Basel, Switzerland). Protein concentration was determined using the Bradford assay (Thermo Fisher Scientific).

For analysis of cell lysates, SKOV3-cells were cultured in McCoy´s 5A Medium supplemented with 10% FCS (Thermo Fisher Scientific). HROC24-cells were cultured in DMEM/Ham´s F12 (Thermo Fisher Scientific) supplemented with 10% FCS and 3 mM glutamine (Thermo Fisher Scientific). Jurkat subclone JMP cells were cultured in RPMI (Thermo Fisher Scientific) supplemented with 10% NCS (Thermo Fisher Scientific). Huvec cells were cultured in Medium 199 without phenol red (Thermo Fisher Scientific) supplemented with 6.6% FCS and 33% EBM™-2 Endothelial Cell Growth Basal Medium-2 (Lonza, Basel, Switzerland). HELA cells (DSMZ ACC-57) and HEK293 cells (DSMZ ACC-305) were cultured in DMEM (Thermo Fisher Scientific) supplemented with 10% FCS. THP-1 cells (ATCC TIB-202) were cultured in RPMI supplemented with 10% FCS. Cells were washed in DPBS (Sigma-Aldrich), before being lysed in M-PER buffer (Thermo Fisher Scientific) supplemented with protease inhibitor cOmplete EDTA-free (Roche) and Phosphatase Stop (Roche). Protein concentration was determined using the Bradford assay (Thermo Fisher Scientific).

### *Immunoprecipitation*

AMPylated proteins were precipitated from purified recombinant BiP and BiP-AMP or CHO-K1 lysates stressed with either thapsigargin or cycloheximide with antibody 17G6 using Pierce ChIP-grade Protein A/G Magnetic Beads (Thermo Fisher Scientific) according to the manufacturer's protocol. In short, in a total volume of 500µl 20 µg recombinant protein or 1 mg total protein lysate were incubated with 10 µg 17G6 antibody in 25 mM Tris pH 7.4, 150 mM NaCl, 1 mM EDTA, 5% glycerol, 1% NP40 overnight at 4°C, before being precipitated with 25 µl equilibrated beads for 1 h at room temperature. Beads were washed twice with the binding buffer, before AMPylated proteins were eluted with 100 µl 1x Laemmli for 15 min at 30 °C. Lysate elutions were concentrated to 20µl in a SpeedVac alpha RVC (Christ) without heat. For recombinant protein samples 7.5 µl each of input and unbound sample supplemented with 5x Laemmli buffer and 2.5 µl elution, for lysate samples 5 µl each of input and unbound sample supplemented with 6x Laemmli buffer and 20 µl of concentrated elution were analyzed by 12% SDS PAGE and WB as described.

For immunoprecipitation after methanol/chloroform precipitation, 250 µg CHO-K1 lysates untreated or stressed with either thapsigargin or cycloheximide were diluted with 3 volumes of methanol and vortexed, before 1 volume of chloroform (Sigma-Aldrich) was added and samples were vortexed. 3 volumes of water were added, samples vortexed, before centrifugation at 15 000 rpm for 2 min at 4°C. The aqueous upper phase was carefully aspirated, 3 volumes of methanol were added, samples vortexed, before centrifugation at 15 000 rpm for 5 min at 4°C. Afterwards the supernatant was removed and protein pellets left to dry. Protein pellets were resuspended in 25 µl 1 M NaOH, before 80 µl binding buffer (25 mM Tris pH 7.4, 150 mM NaCl, 5% glycerol) were added and pH adjusted to 7.5 with 10% HCl. 2.5 µg 17G6 antibody were added and incubated overnight at 4°C, before being precipitated with 25 µl equilibrated beads for 1 h at room temperature. Beads were washed twice with the binding buffer, before AMPylated proteins were eluted with 50 µl 1x Laemmli for 15 min at 30 °C.

5 µl each of input and unbound sample supplemented with 6x Laemmli buffer and 20 µl of elution were analyzed by 12% SDS PAGE and WB as described.

***Western blotting***

200 ng or 50 ng recombinant protein as indicated or 20 µg cell lysate, respectively, were separated by 15% or 12% SDS-PAGE and protein was transferred to MeOH-activated Immobilon®-P membrane (Merck Millipore) using Whatman paper and a transfer buffer of 48 mM Tris, 39 mM glycine, 1.3 mM SDS, 20% methanol. For the blotting procedure, a constant current of 0.7 $mA/cm^2$ was applied to the V20-SDP semi-dry blotter (SCIE-PLAS, Cambourne, UK) for 2 h. After blotting, the PVDF membrane was blocked with Roti®-Block (Carl Roth, Karlsruhe, Germany) in Tris-buffered saline containing 0.1% Tween20 (TBS-T) for 1 h. Subsequently, the primary antibody was added to the blocking solution and incubated overnight at 4°C. Afterwards, the membrane was washed three times with TBS-T for 10 minutes and then incubated with a secondary antibody-peroxidase conjugate in TBS-T for 1 h. Again, the membrane was washed in TBS-T three times for 10 min, before the peroxidase signal was developed with the SuperSignal™ West Dura (Thermo Fisher Scientific) and chemoluminescence was detected using the Intas ECL Chemocam (Intas Science Imaging Instruments, Göttingen, Germany). Antibodies: Mouse pre-immune serum and antiserum after $3^{rd}$ immunization (GenScript) was used 1:1000. Cell supernatant from hybridoma clones and subclones (GenScript) was used 1:10. Purified monoclonal mouse anti-AMP antibodies 17G6, 1G11, 7C11 (GenScript) were used 1:1000 at 0.5 µg/ml. Monoclonal mouse anti-GAPDH sc-47724 (Santa Cruz Biotechnology, Dallas, Texas) was used 1:1000. Polyclonal rabbit anti-histone H3 antibody ab1791 (Abcam, Cambridge, UK) was used 1:5000. Polyclonal rabbit anti-AMPylated Tyrosine Antibody ABS184 (Merck Millipore) was used 1:1000. Polyclonal rabbit anti-pan-AMPylated Threonine Antibody 09-890 (Sigma-Aldrich) was used 1:2000. Recombinant rabbit anti-pan-ADP-ribose binding reagent MABE1016 (Merck Millipore) was used 1:1000. Polyclonal rabbit anti-GRP78/BiP antibody PA5-34941 (Thermo Fisher Scientific) was used 1:5000. Secondary goat anti-mouse lgG (H + L) HRP conjugate (Thermo Fisher Scientific) was used 1:20000. Secondary goat anti-rabbit IgG HRP (Sigma-Aldrich) was used 1:40000. Additives as indicated were added during the primary antibody incubation step at a final concentration of 1 µM for adenosine (Jena Bioscience), AMP (Sigma-Aldrich), ADP (Biosynth Carbosynth, Staad, Switzerland), ATP (Biosynth Carbosynth), ADPR (Sigma-Aldrich), $NAD^+$ (Biosynth Carbosynth) or 1 mM for $MnCl_2$, $MgCl_2$ (VWR International, Radnor, Pennsylvania), respectively. Hydroxylamine treatment was performed as previously described (Gibson et al., 2017). In short, after development of membrane with anti-AMP antibodies, the membrane was incubated with 1 M hydroxylamine (Sigma-Aldrich) in blocking solution for 8 h at room temperature, washed three times in TBS-T, blocked again for 1 h at room temperature, and proceeded with a second round of anti-AMP antibody probing. All western blots on recombinant proteins were performed as technical duplicates. Analysis of cell lysate samples was performed as biological duplicate. Representative blots are shown; unless otherwise indicated, rows represent same exposure times from identical blots. All changes in brightness and contrast were applied equally across the entire image. Cropping, e.g. due to different molecular weights, is indicated by a break in the frame. All blots are depicted in full along with their dynamic range in the supplemental information (Data S1).

**Data S1: Uncropped gel and WB images with dynamic range.** Related to Figure 1, Figure 2, Figure 3, Figure 4, Figure S4, Figure S6 and Figure S7.

***Figure 1A***

α-Thr-AMP (Merck)

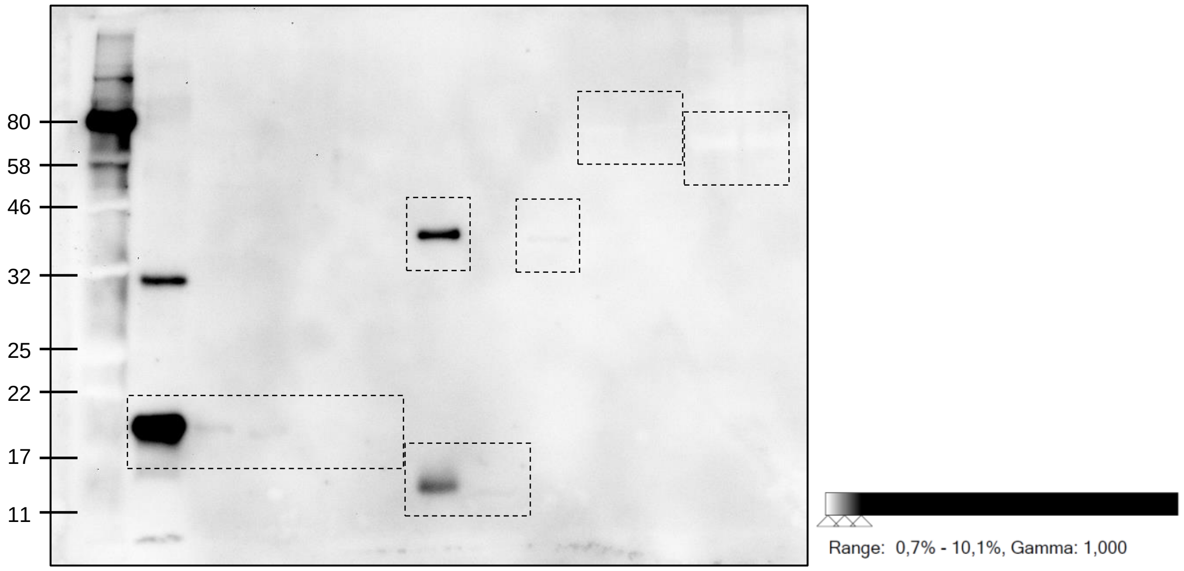


α-Tyr-AMP (Merck)

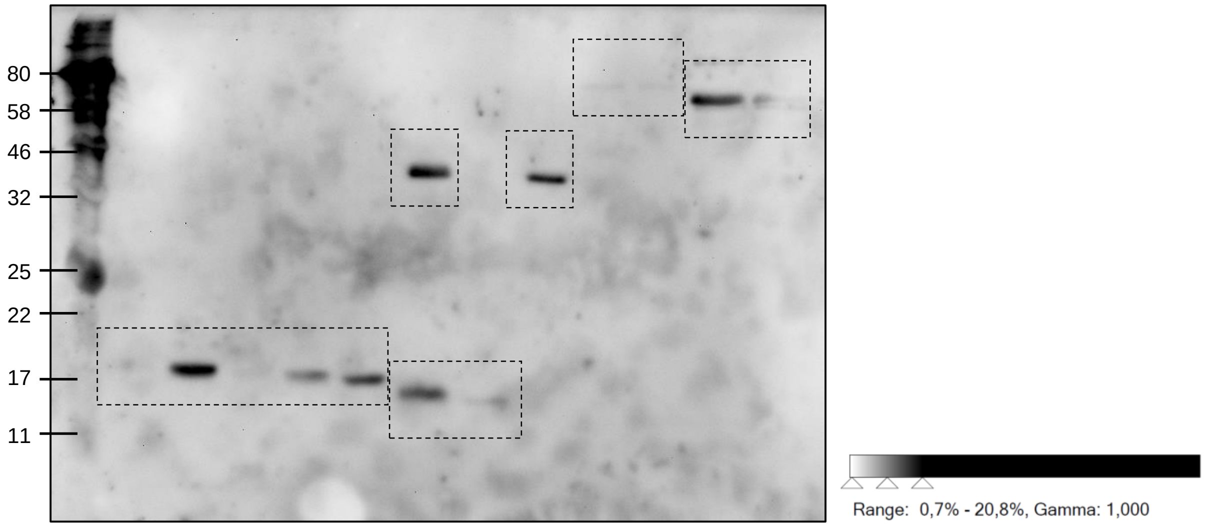


A-pan-ADPR (Merck)

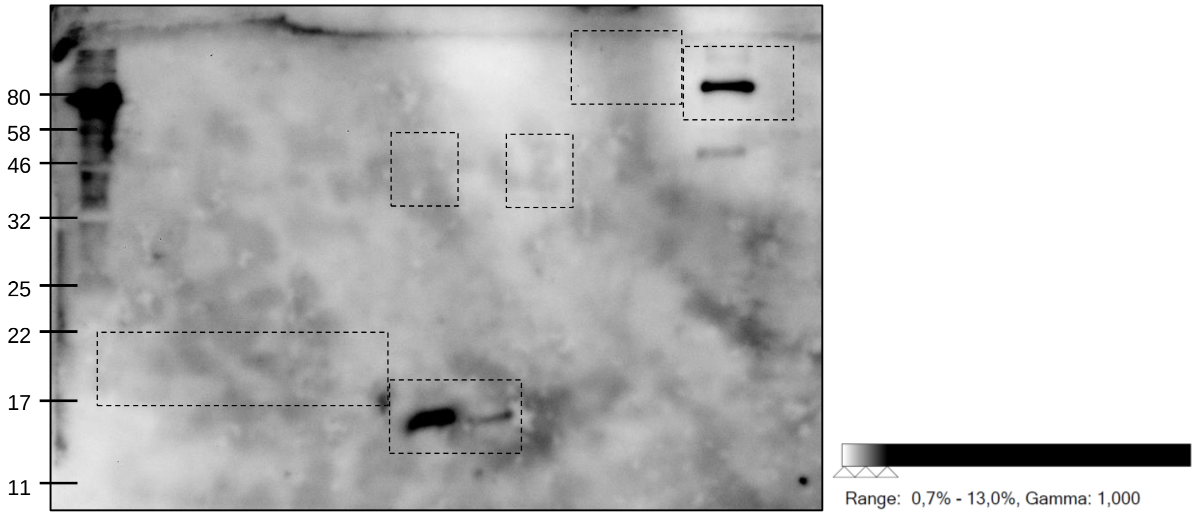

***Figure 2A***

1G11_Cdc42

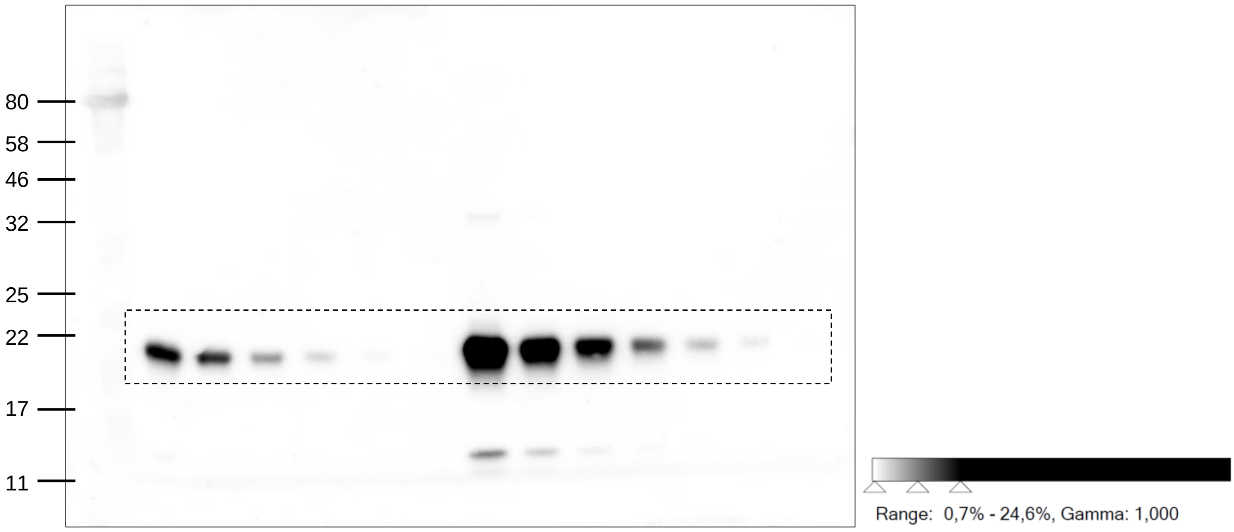


1G11_Rab1

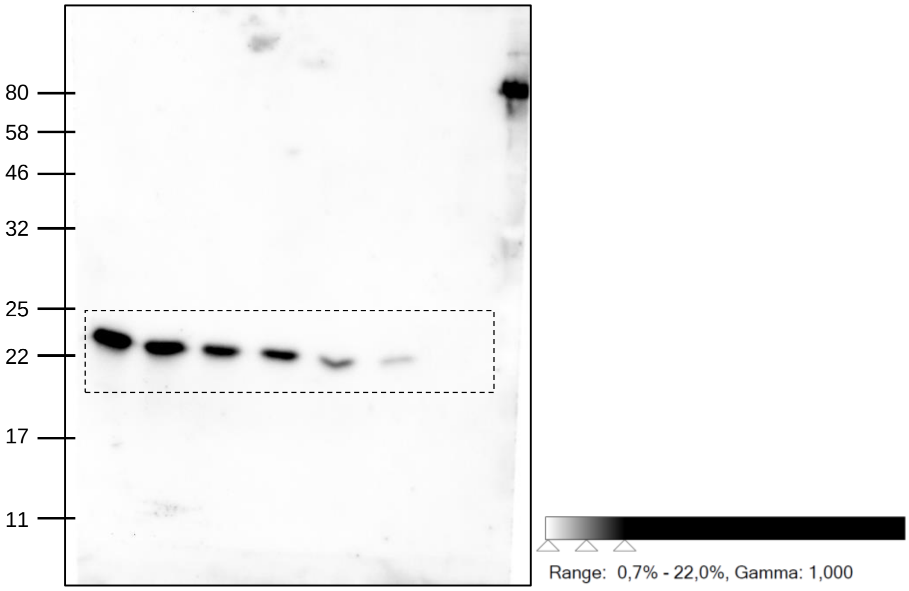


7C11_Cdc42

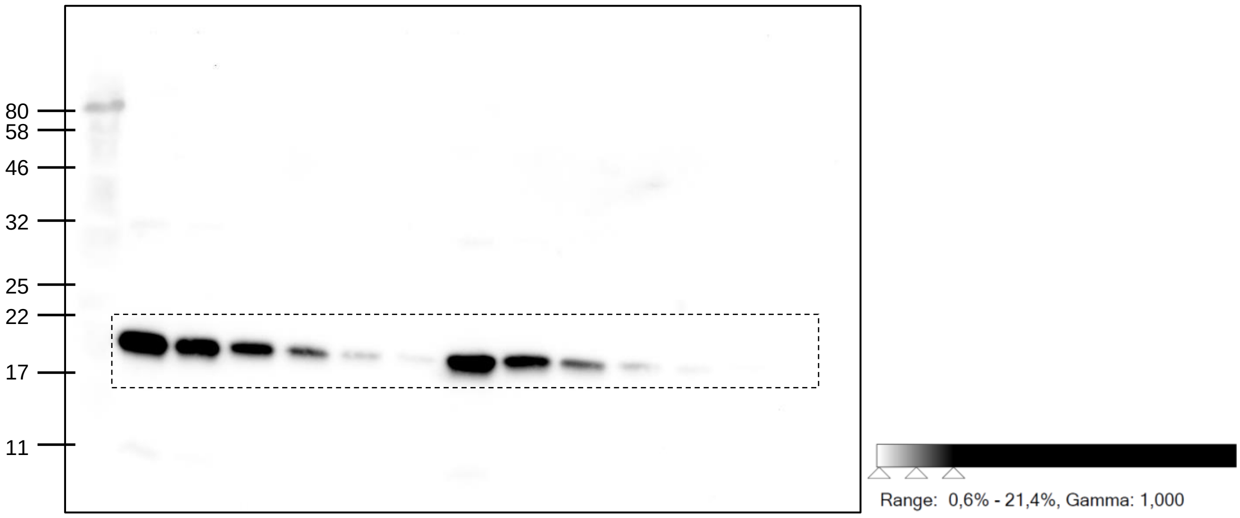


7C11_Rab1

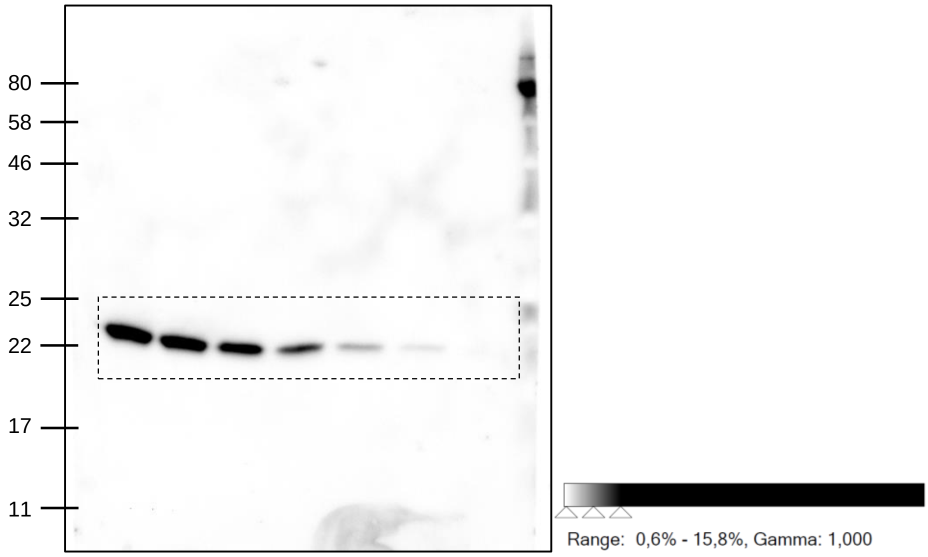


17G6_Cdc42

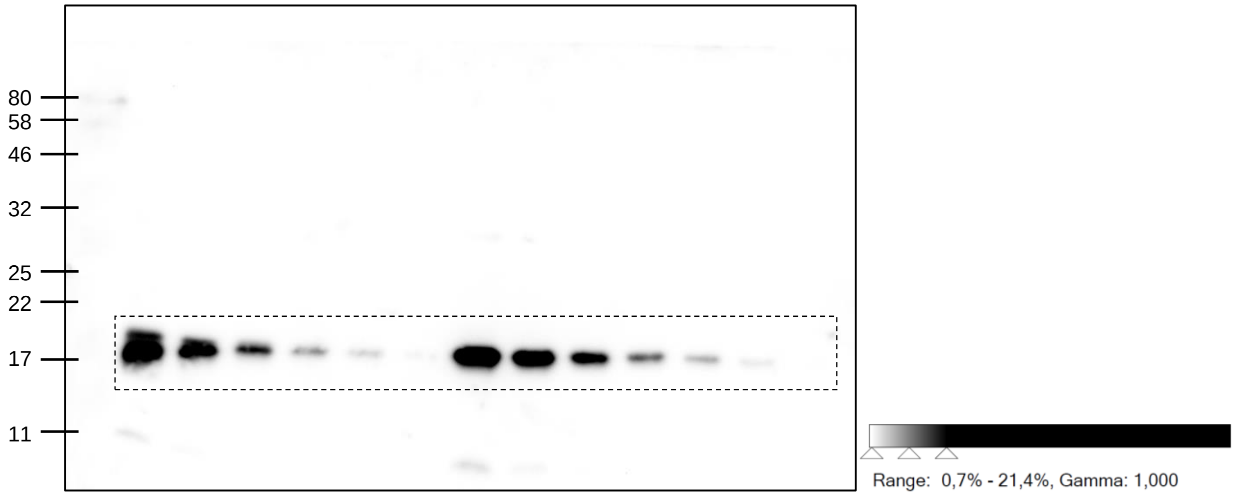


17G6_Rab1

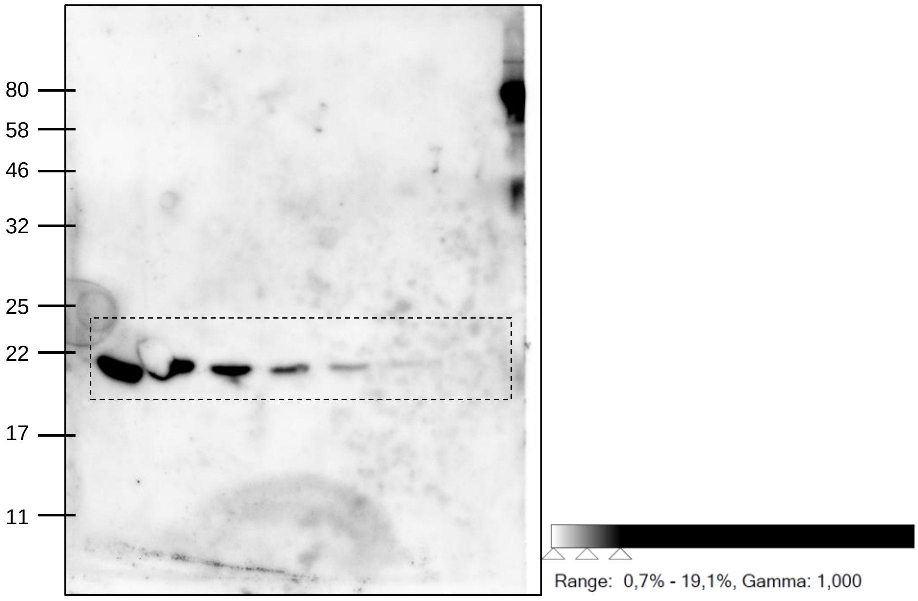

7C11_BiP

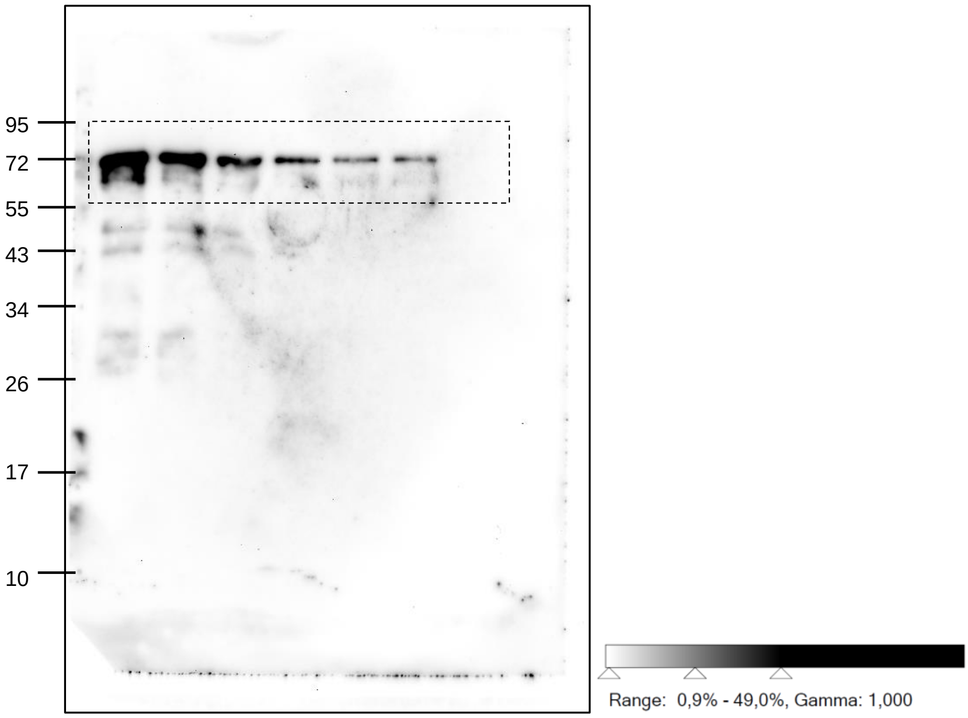


17G6_BiP

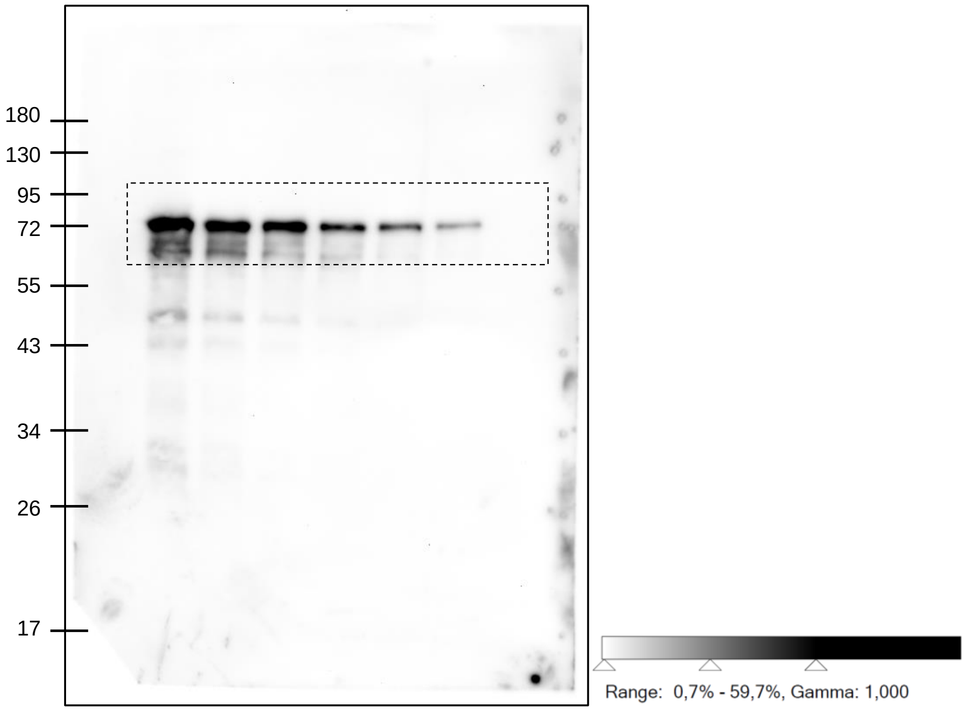

1G11_BiP

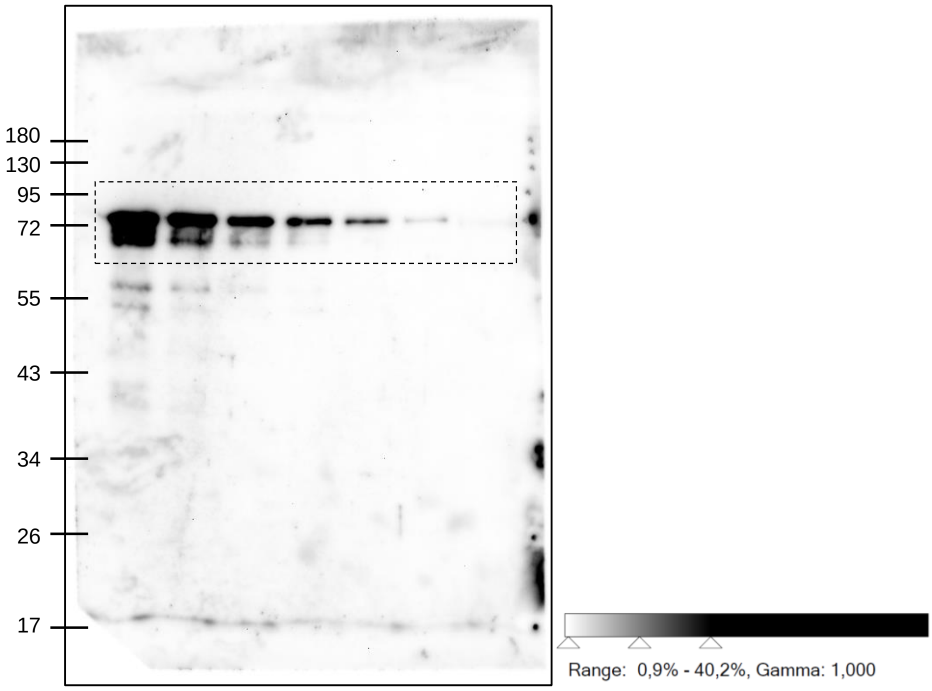

***Figure 2B***

1G11

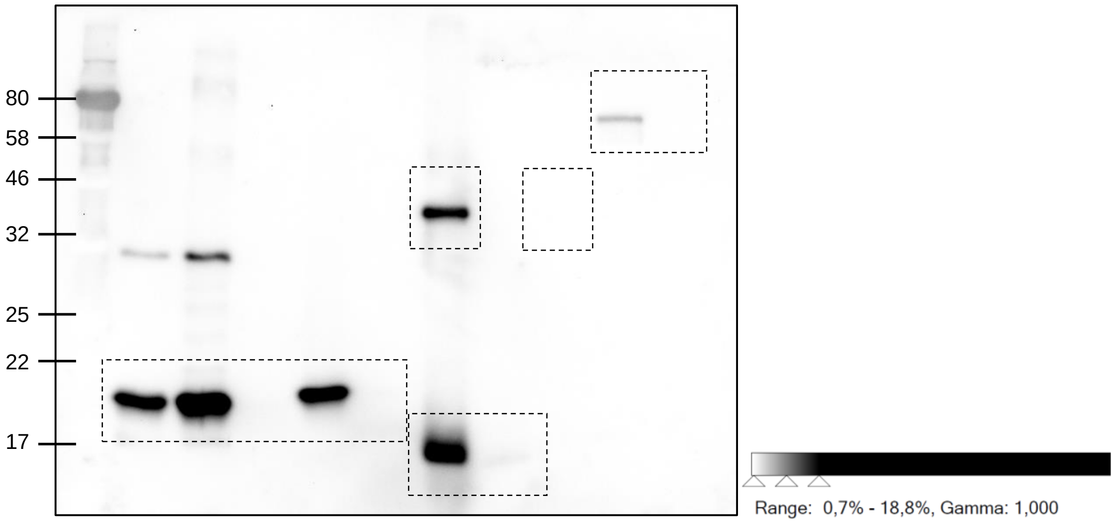


7C11

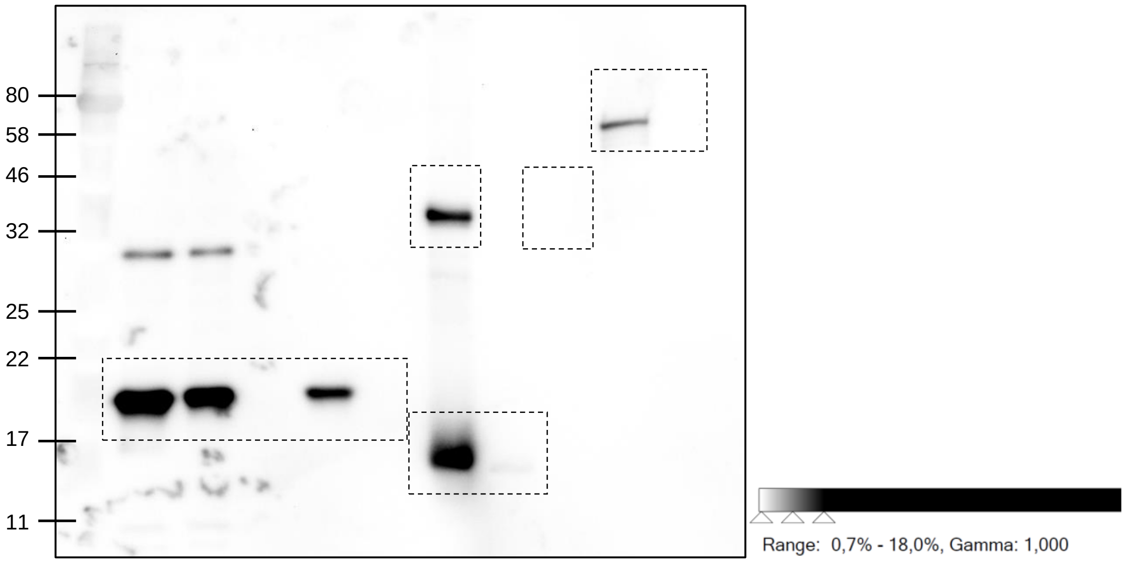


17G6

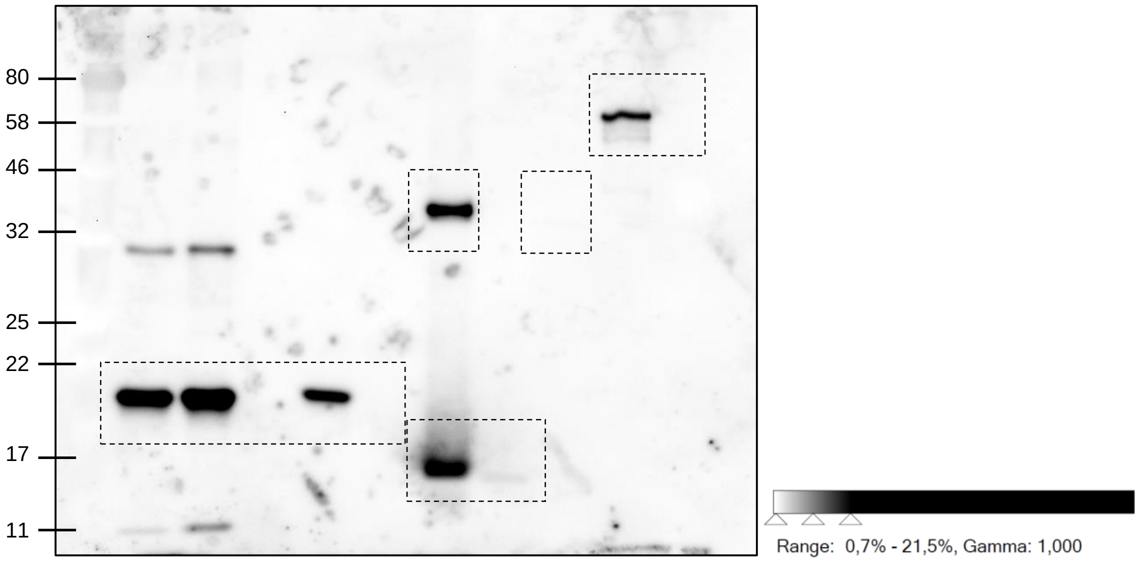

***Figure 2C***

17G6_Cdc42-Tyr-AMP

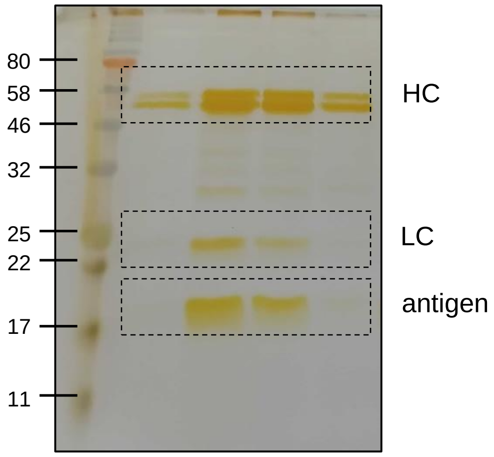


17G6_Cdc42-Thr-AMP (left), _Rab1-AMP (right)

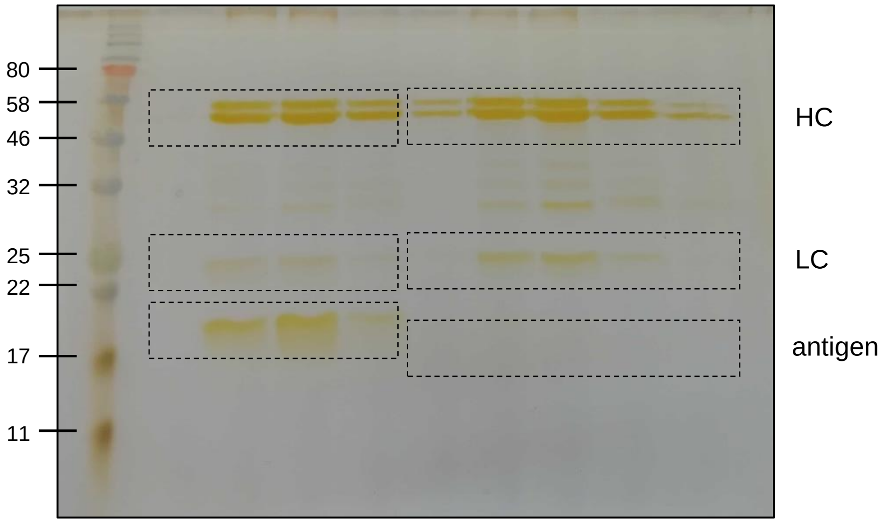


17G6_BiP-AMP

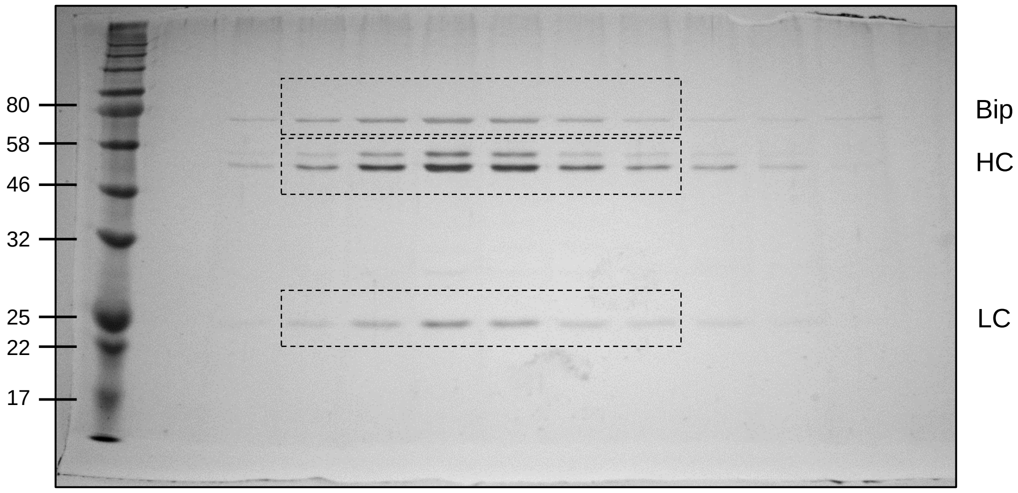

***Figure 2D***

1G11

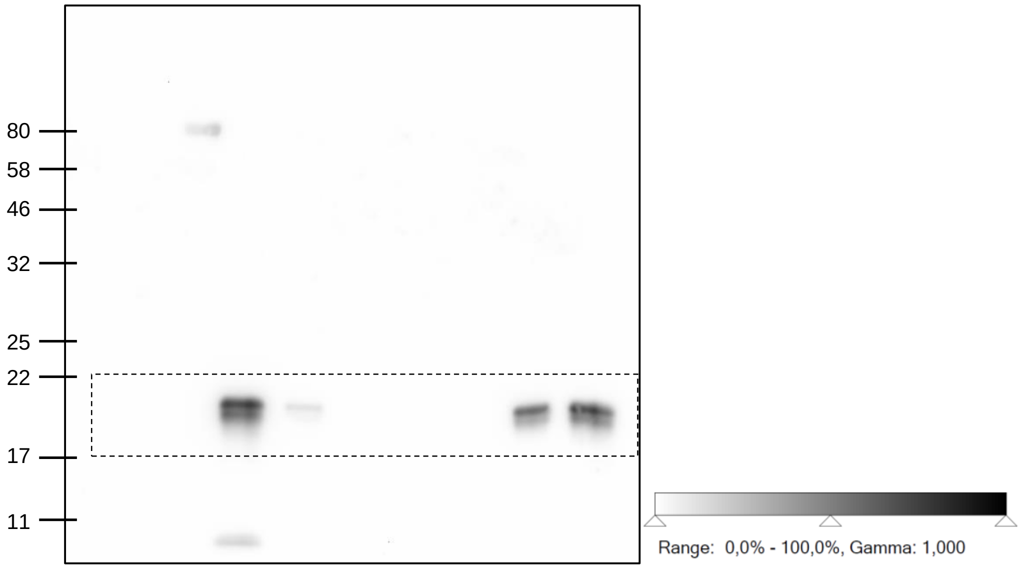


7C11

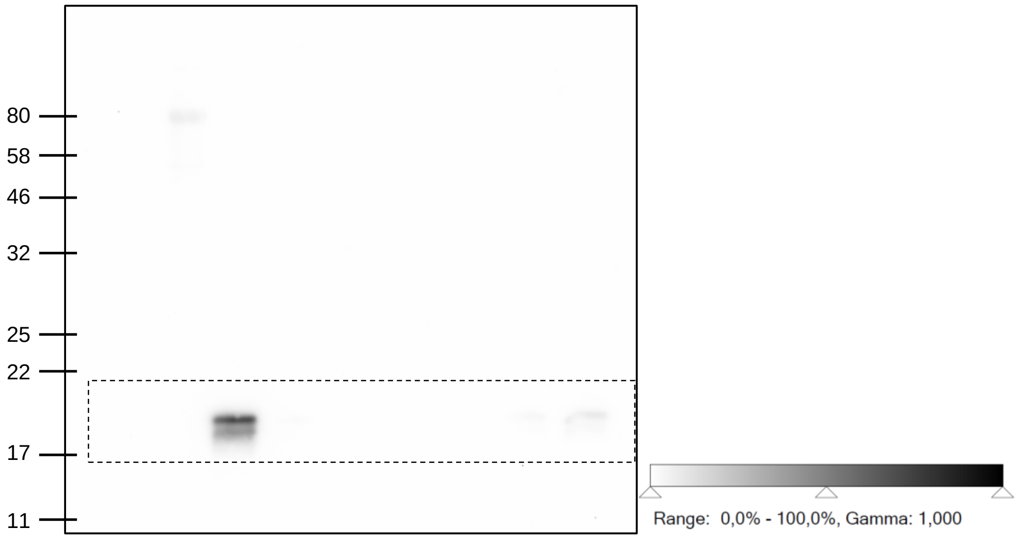


17G6

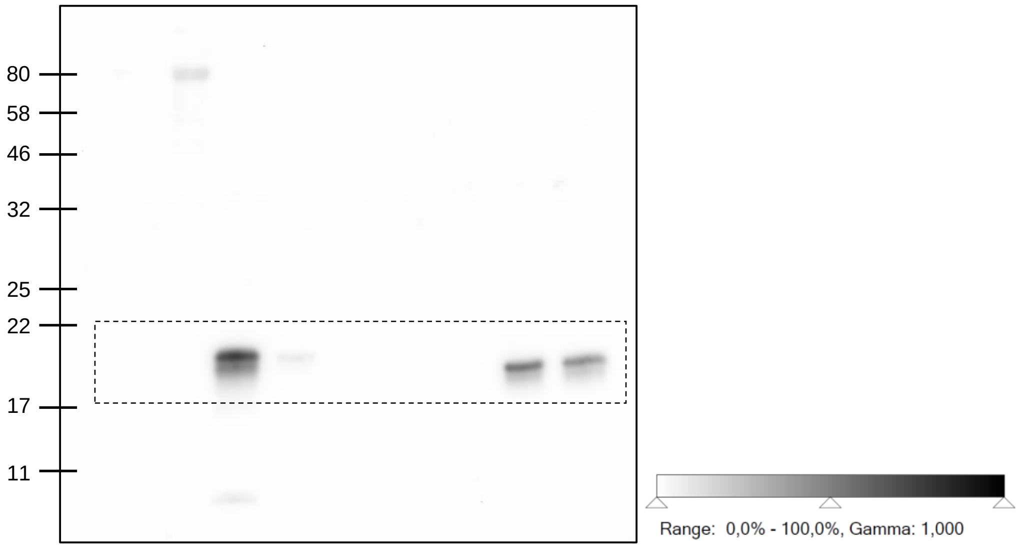

***Figure 2E***

1G11

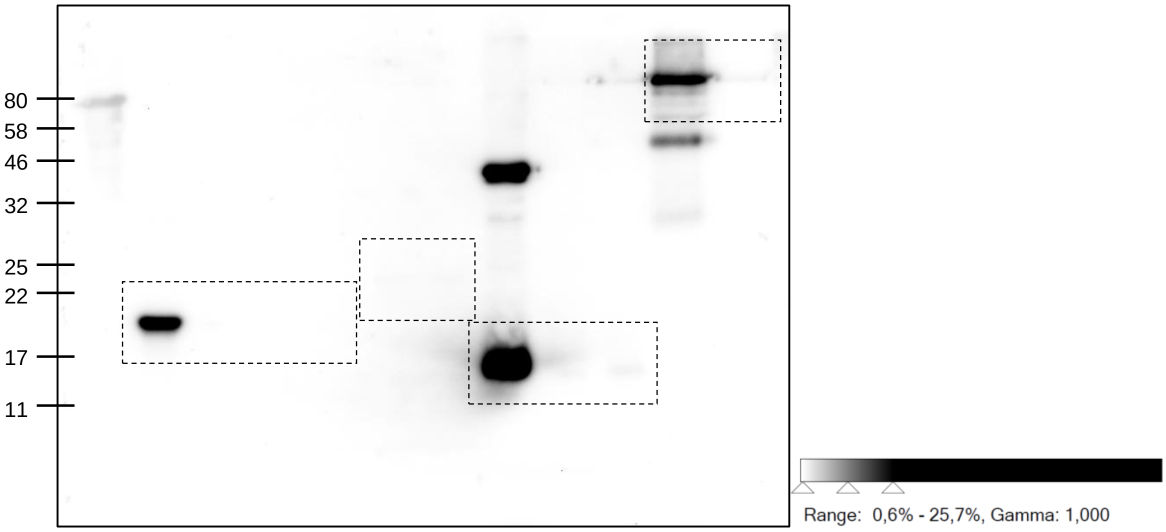


17G6

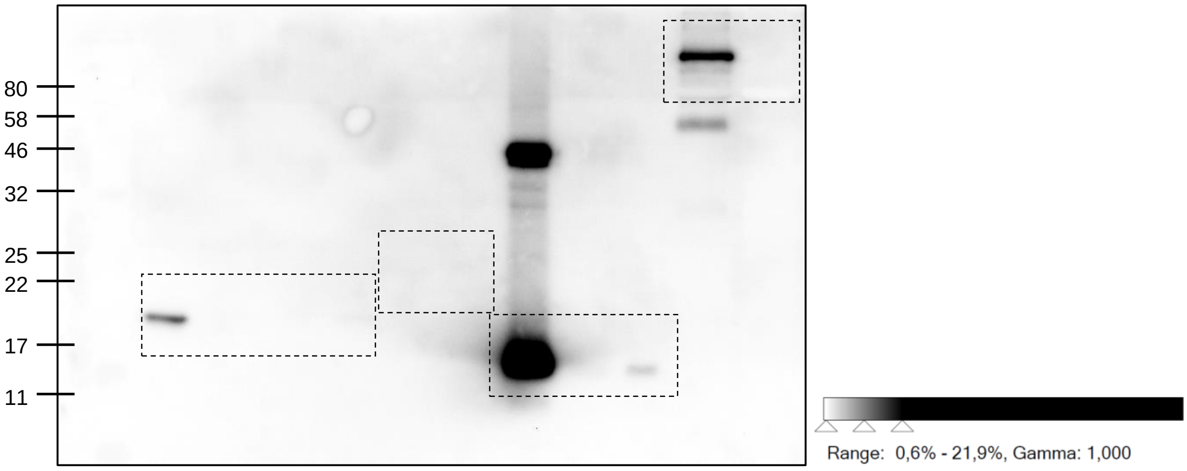


7C11

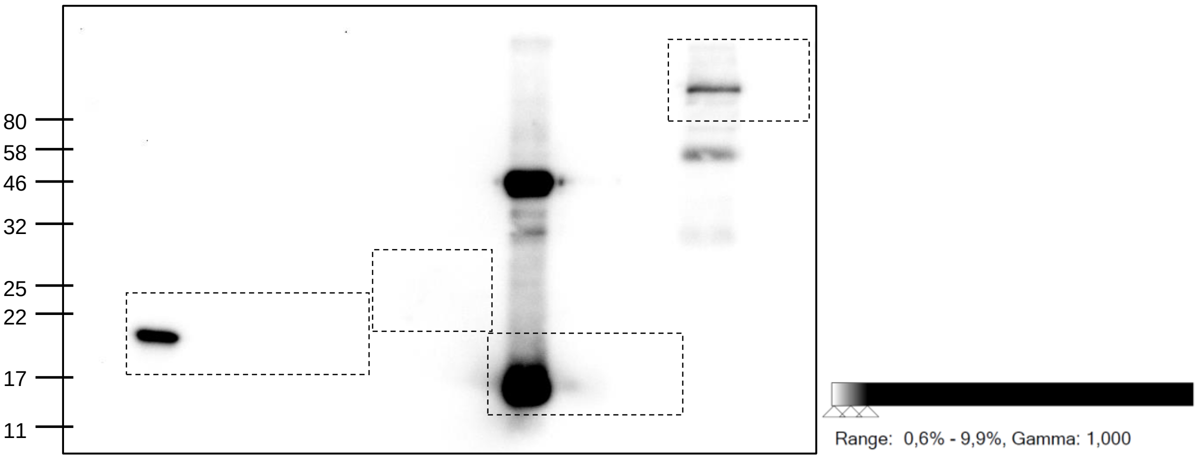

***Figure 3A***

17G6_1 mM $MgCl_2$

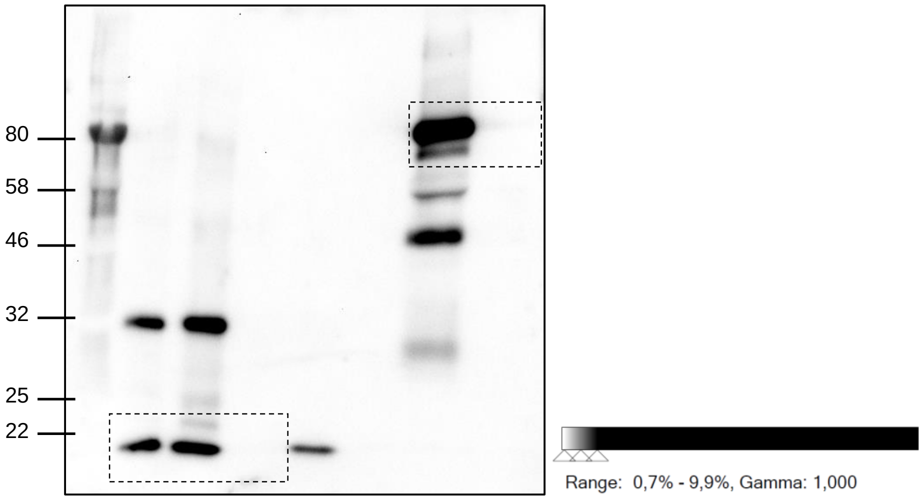


17G6_1 mM $MnCl_2$

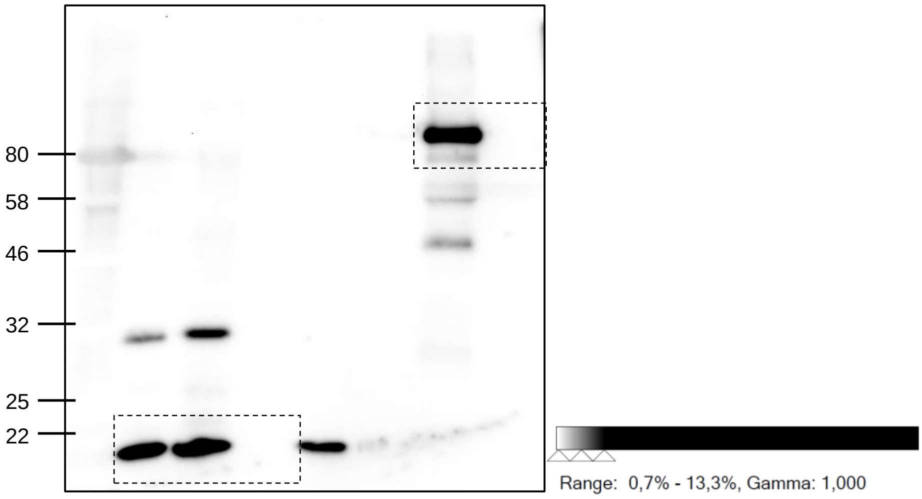


17G6_no additive

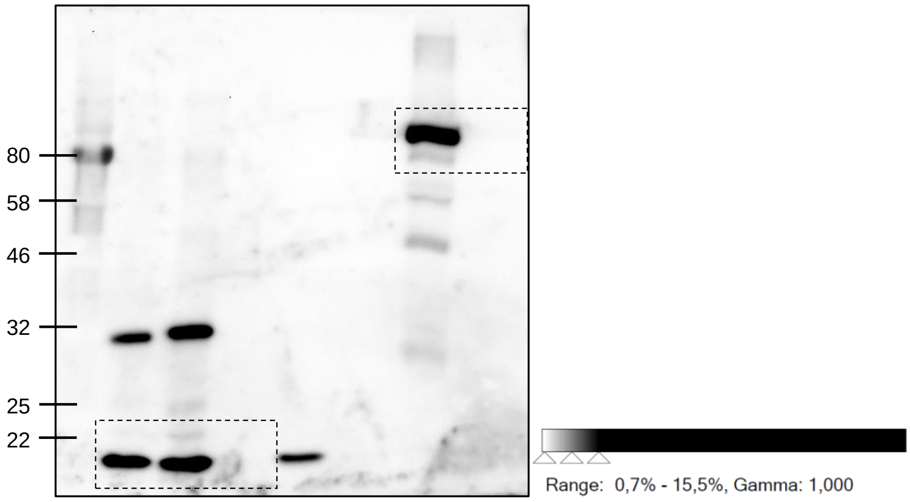

17G6_1 µM adenosine

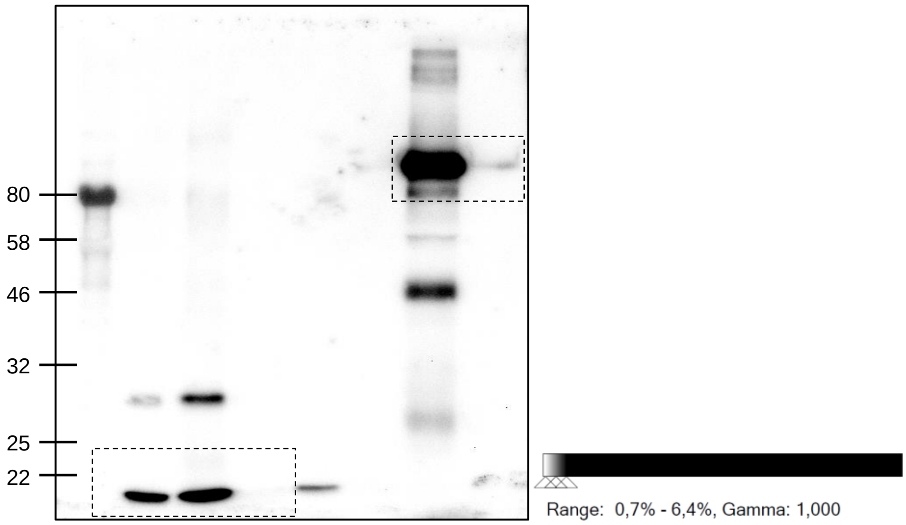


17G6_1 µM ADP

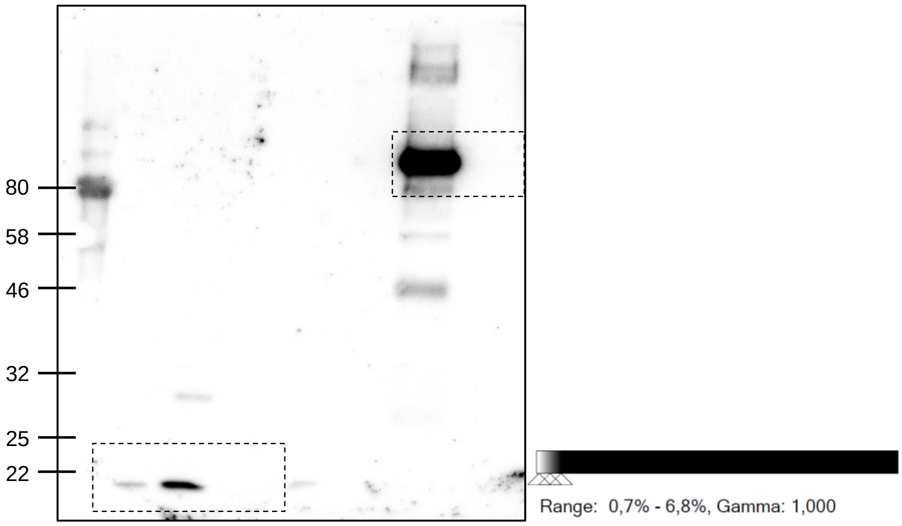


17G6_1 µM AMP

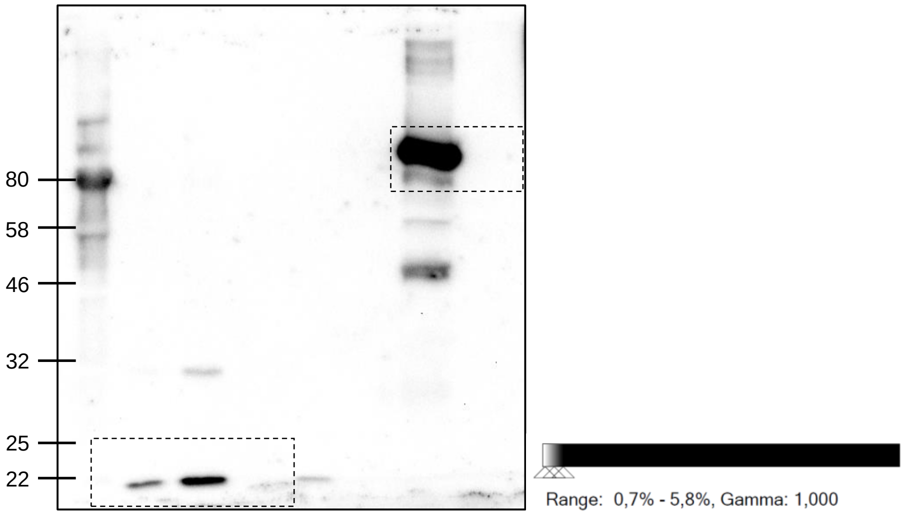

17G6_1 µM ATP

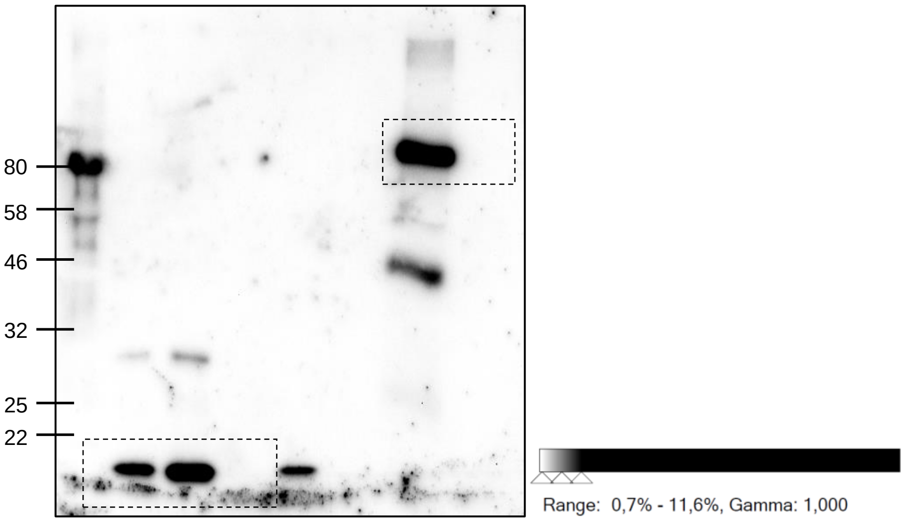


17G6_1 µM NAD+

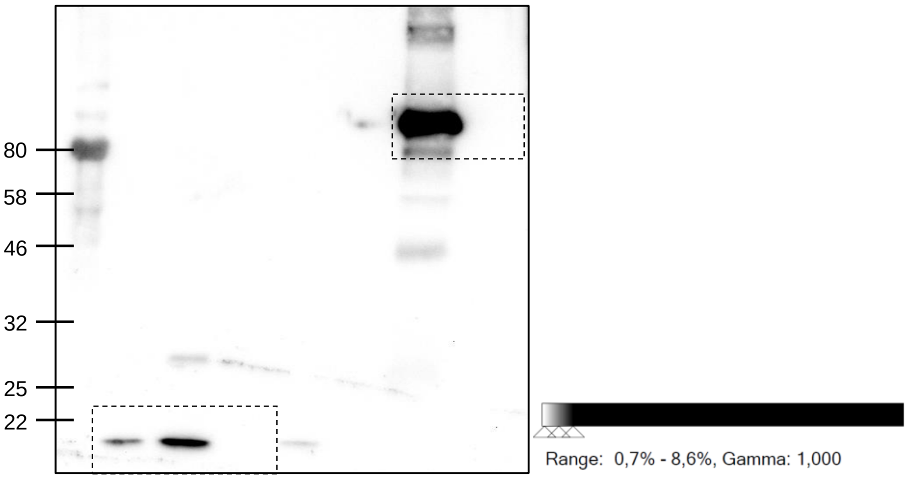


17G6_1 uM ADPR

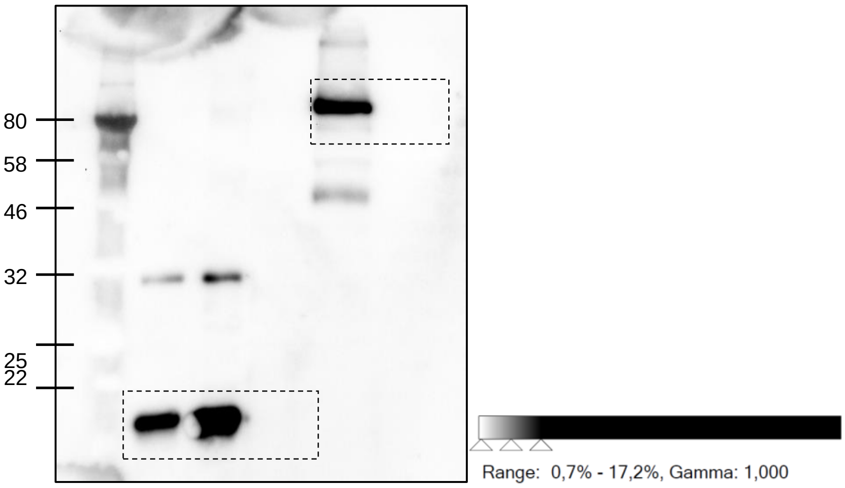

17G6_Hydroxylamine treated (previously 17G6_1 uM ADPR)

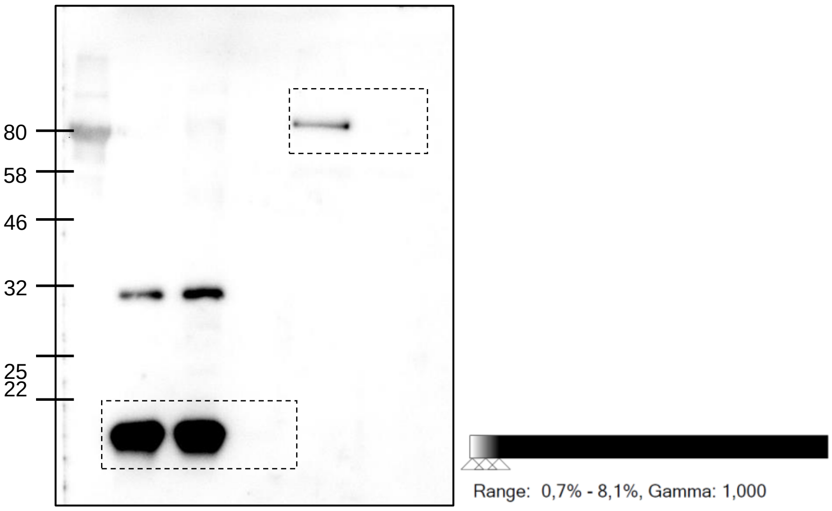

***Figure 3B***

1G11 + $MnCl_2$_Cdc42

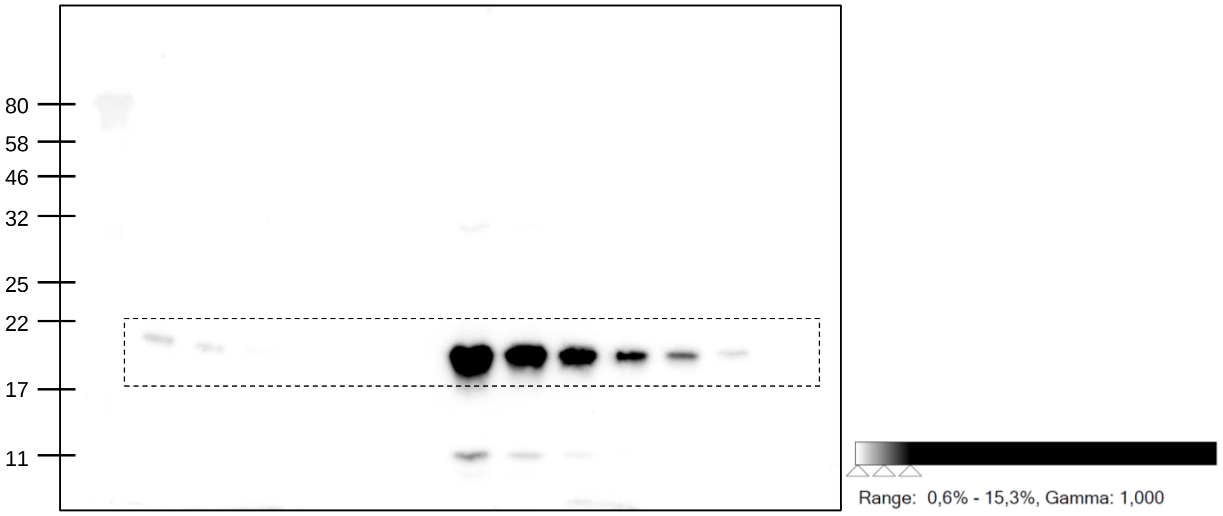


1G11 + $MnCl_2$_Rab1

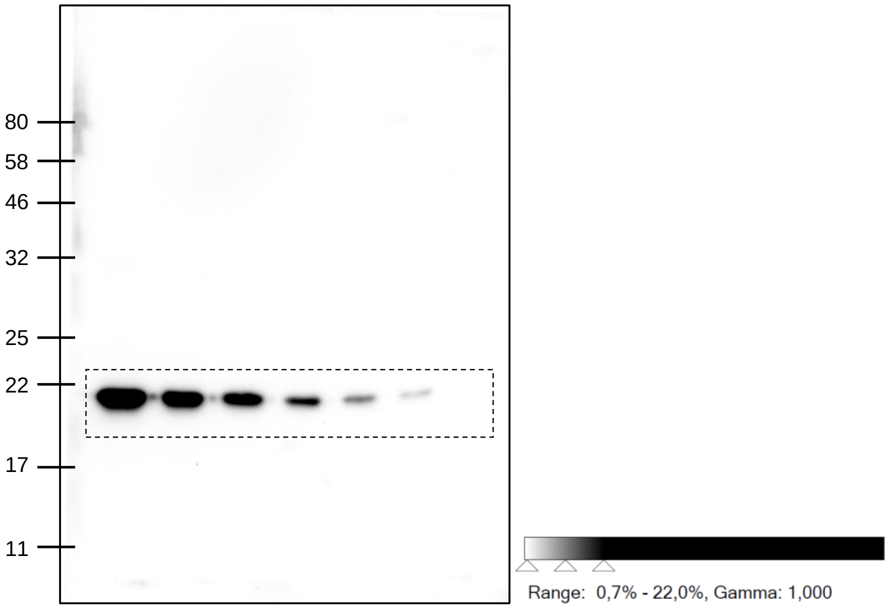


7C11 + $MnCl_2$_Cdc42

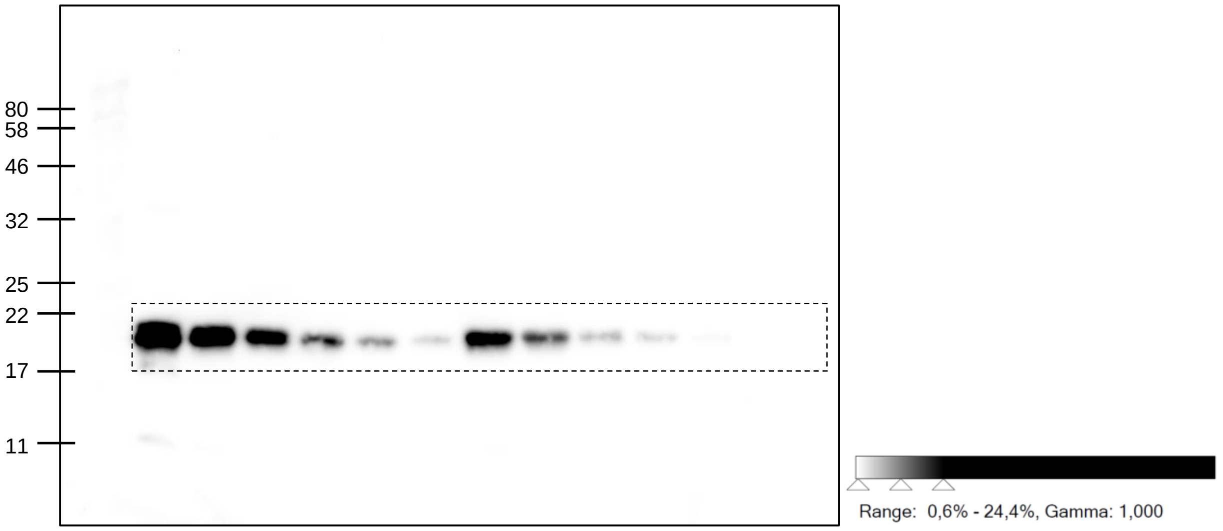

7C11 + $MnCl_2$_Rab1

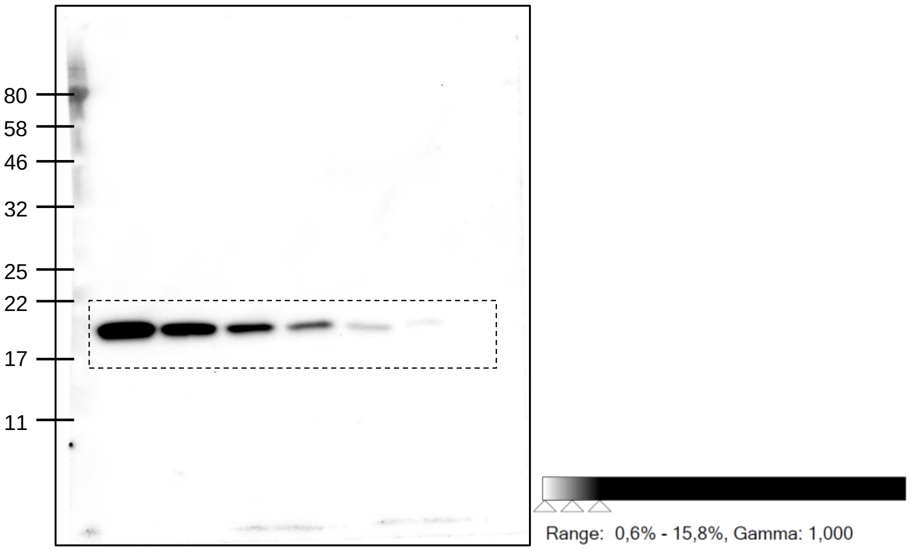


17G6 + $MnCl_2$_Cdc42

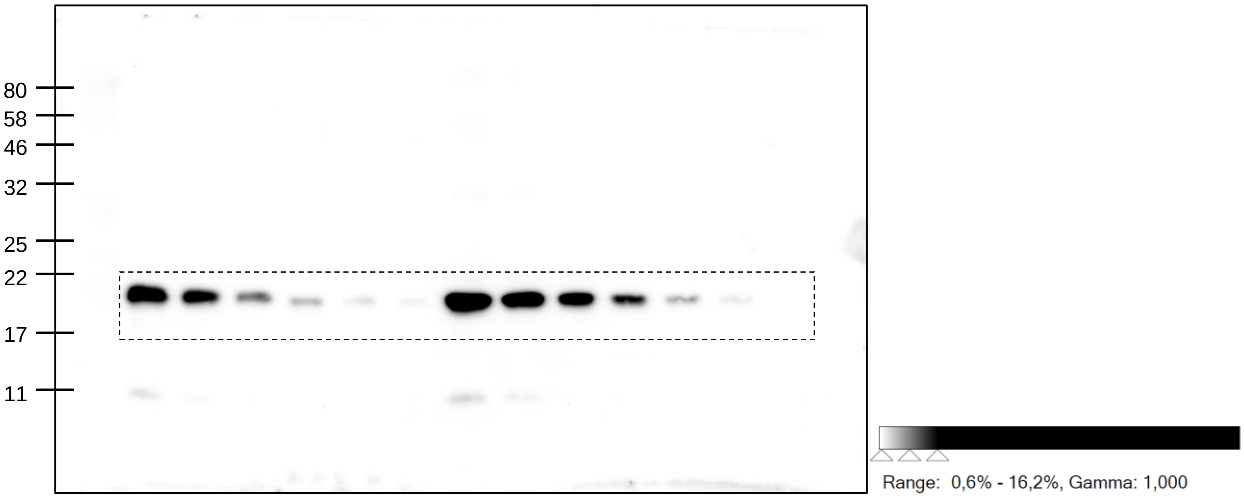


17G6 + $MnCl_2$_Rab1

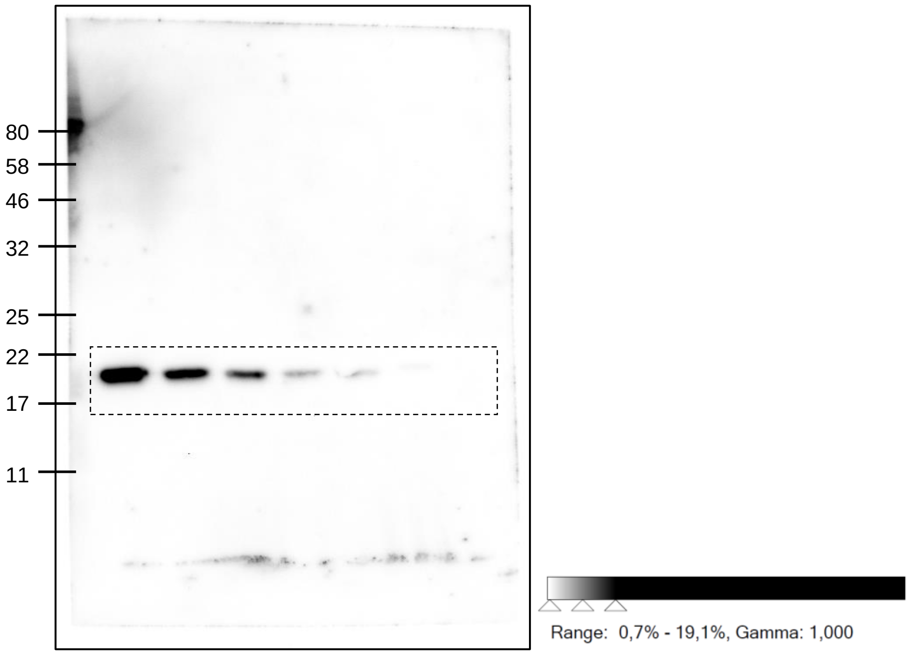

7C11 + $MnCl_2$_BiP

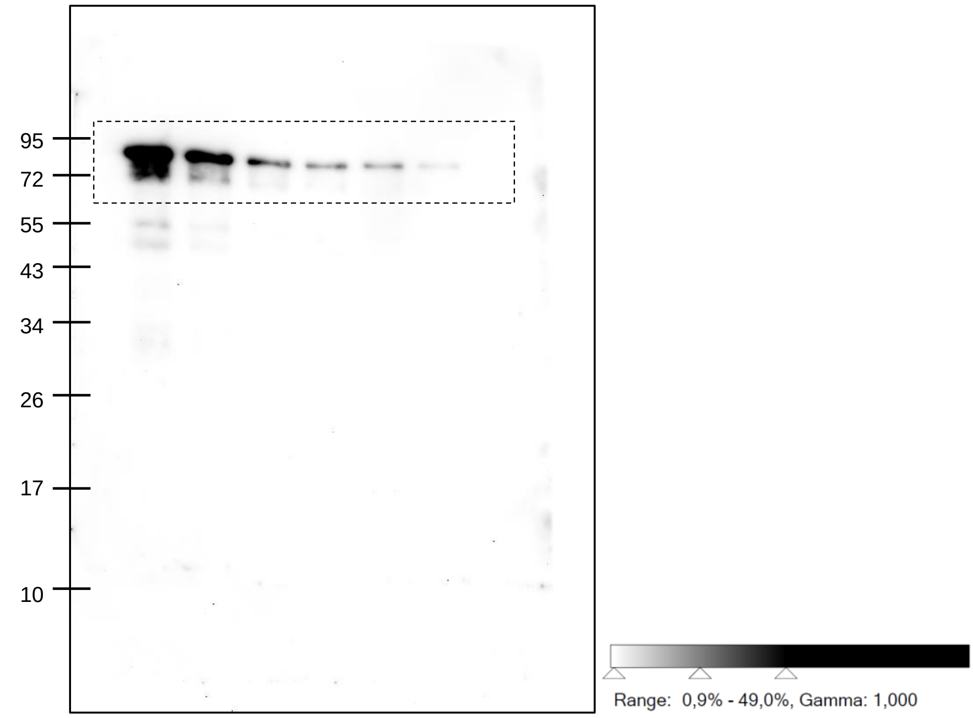


17G6 + $MnCl_2$_BiP

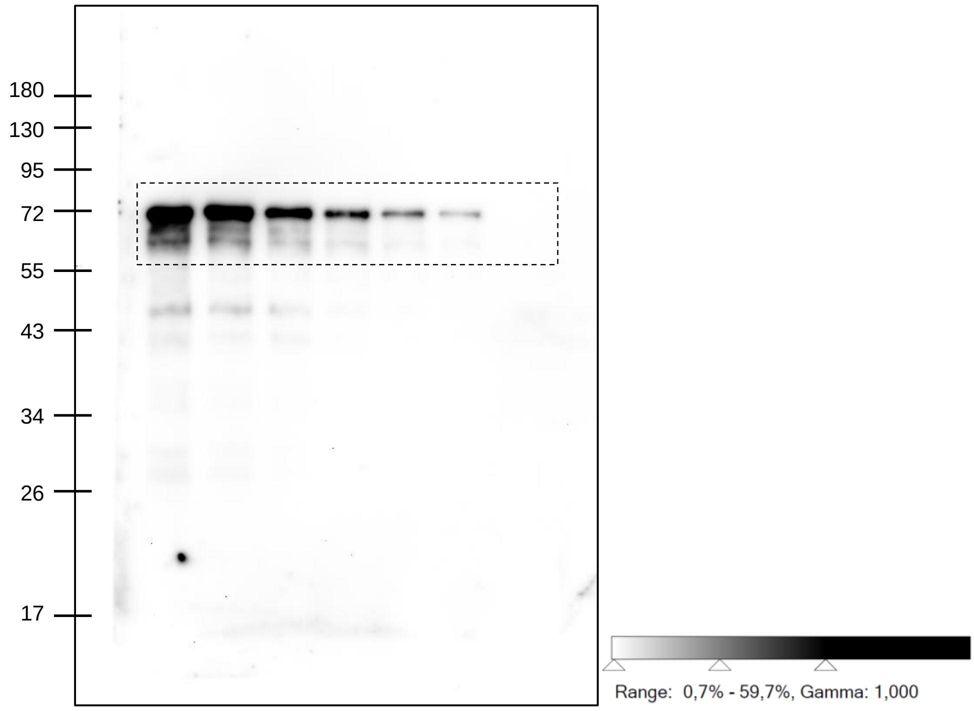

1G11 + $MnCl_2$_BiP

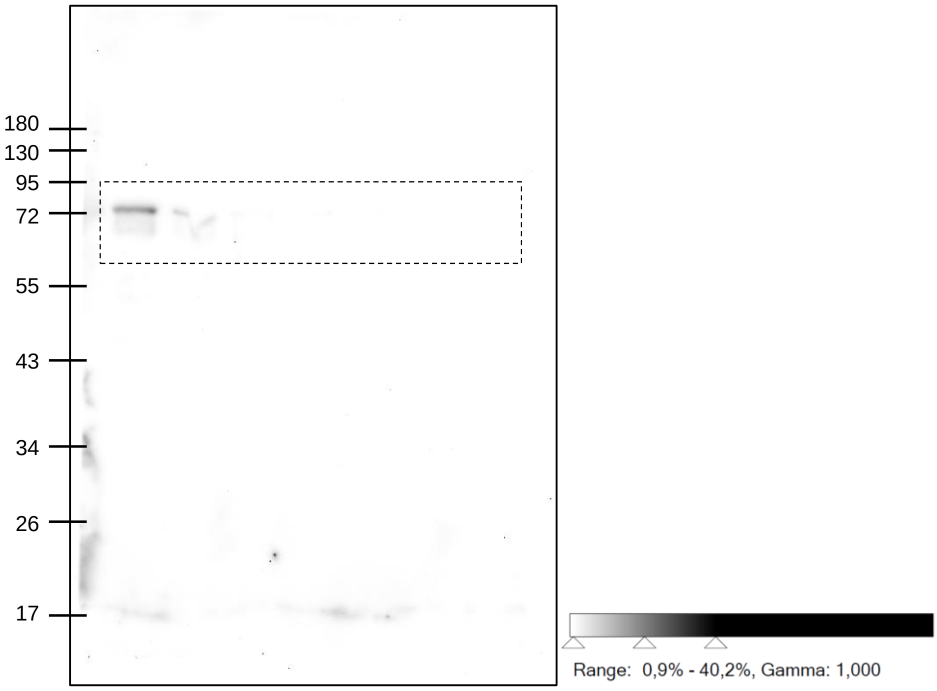

***Figure 3C***

7C11_$MnCl_2$ (left) and _without additive (right)

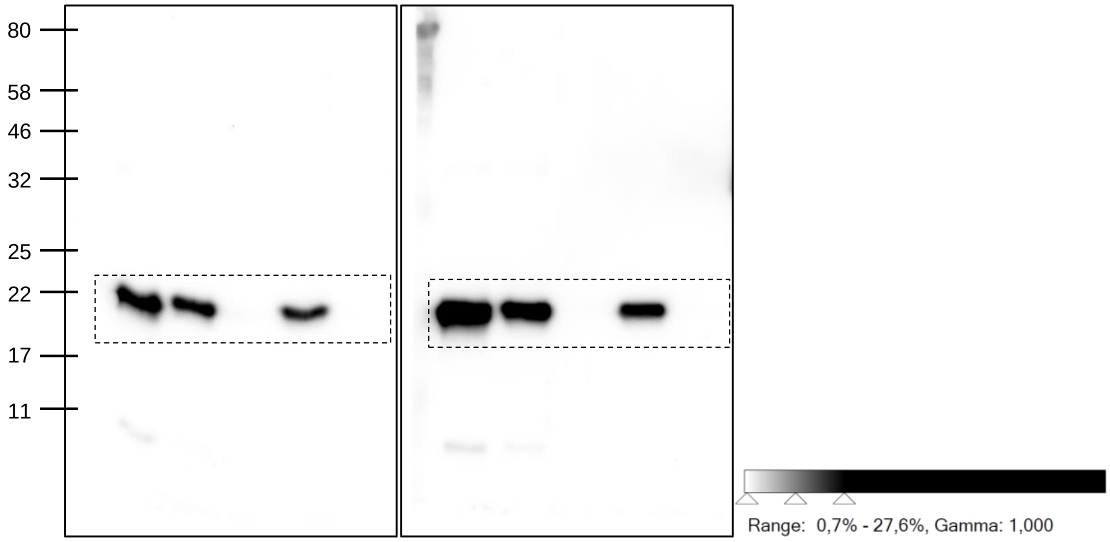


17G6_$MnCl_2$ (left) and _without additive (right)

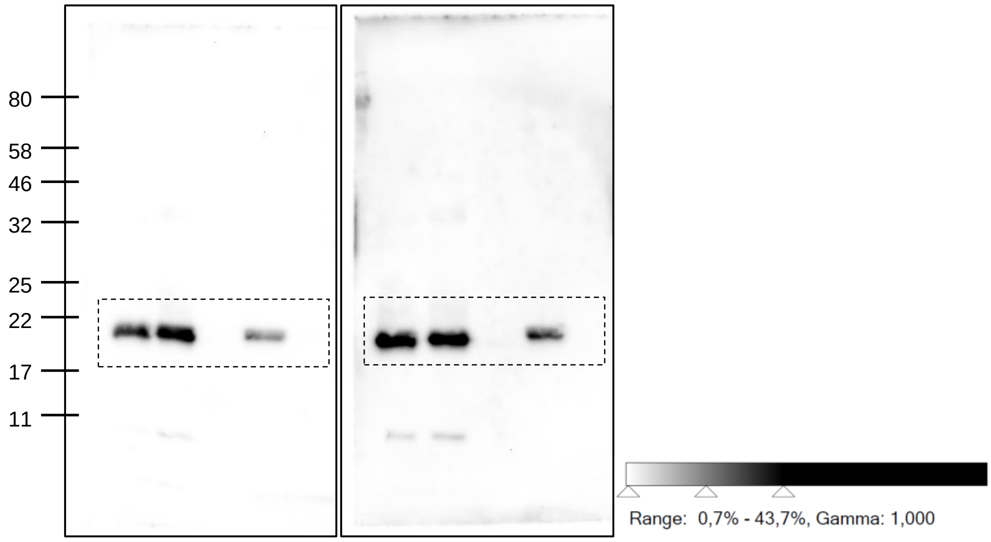


1G11_$MnCl_2$ (left) and _without additive (right)

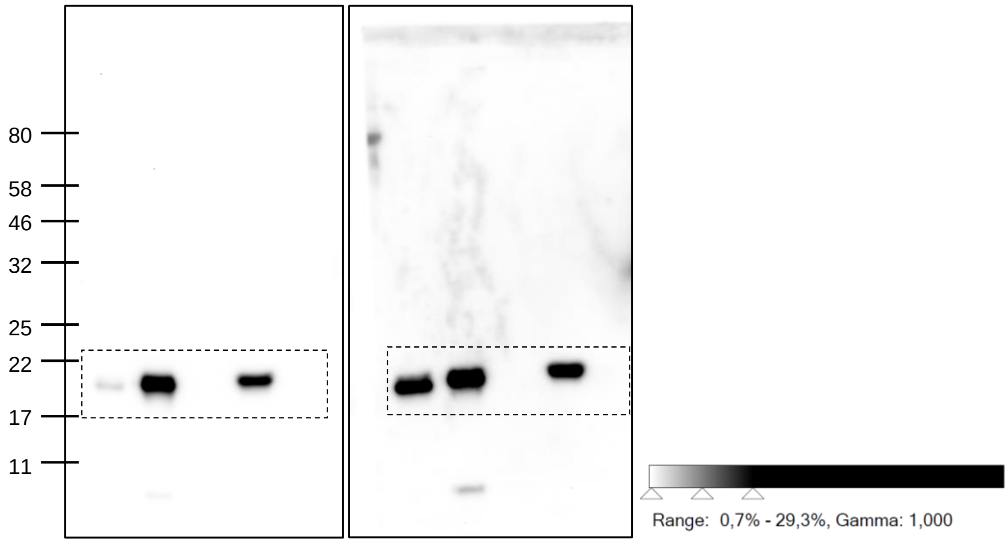

***Figure 4A***

17G6 + $MnCl_2$_ctrl

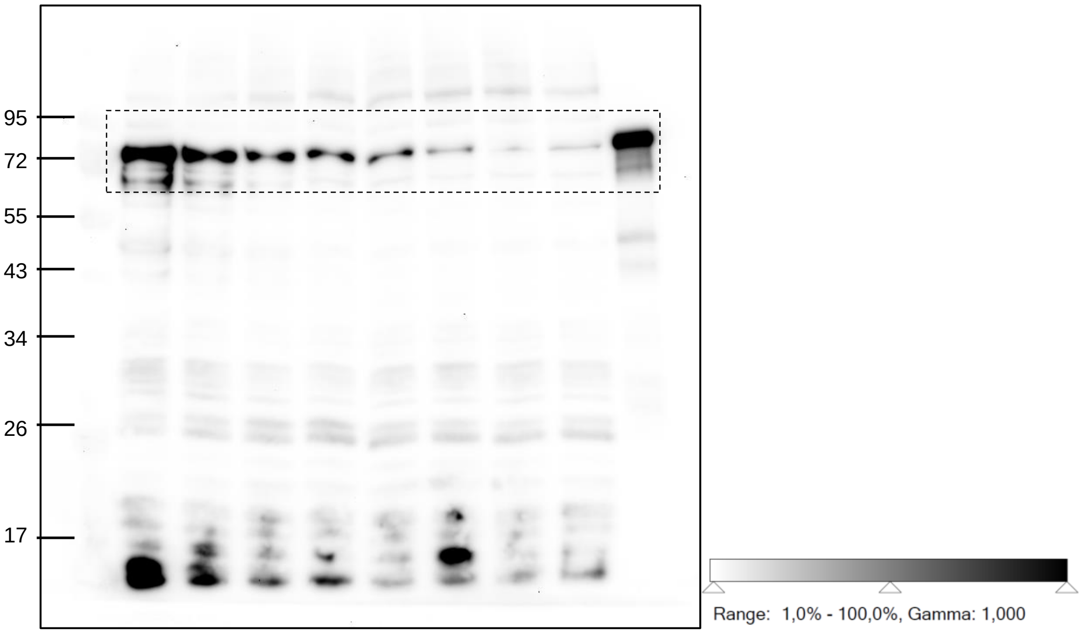


17G6 + $MnCl_2$_2 h TG

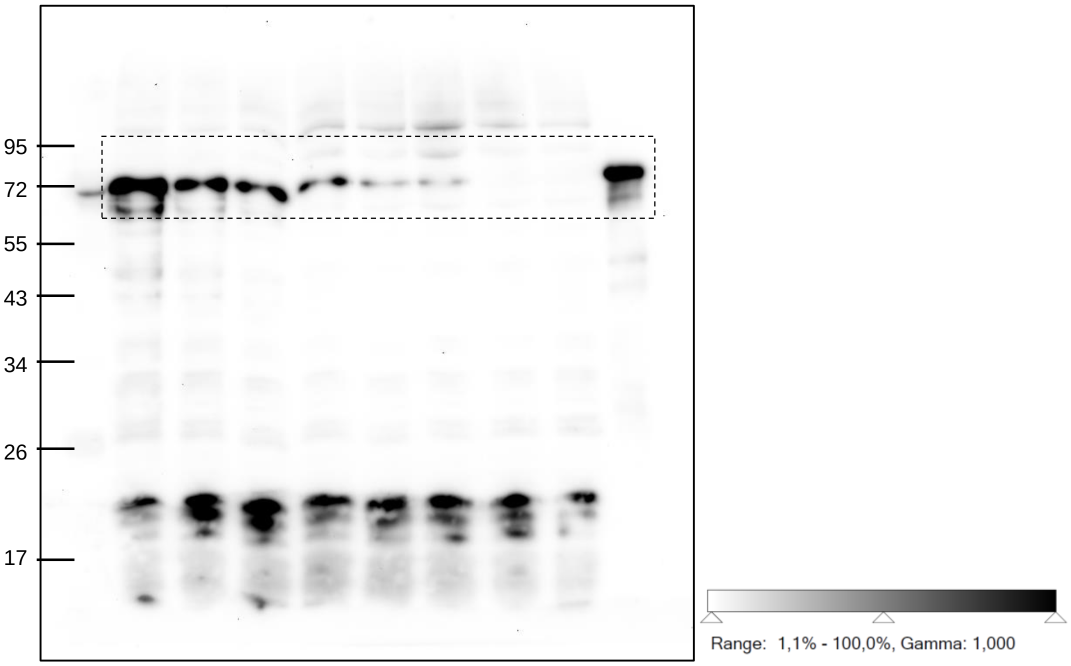

***Figure 4B***

aBiP_IP: beads only_- (left), _BiP (middle) and _BiP-AMP (right)

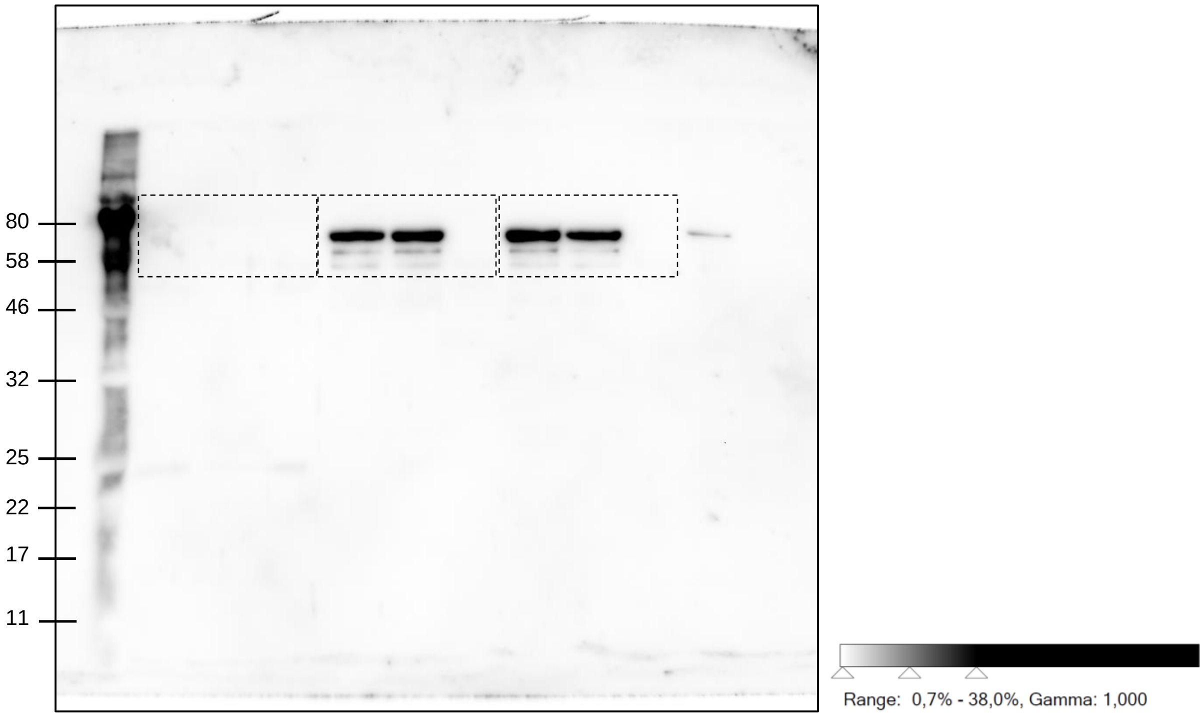


aBiP_IP: 17G6_BiP (left) and _BiP-AMP (right)

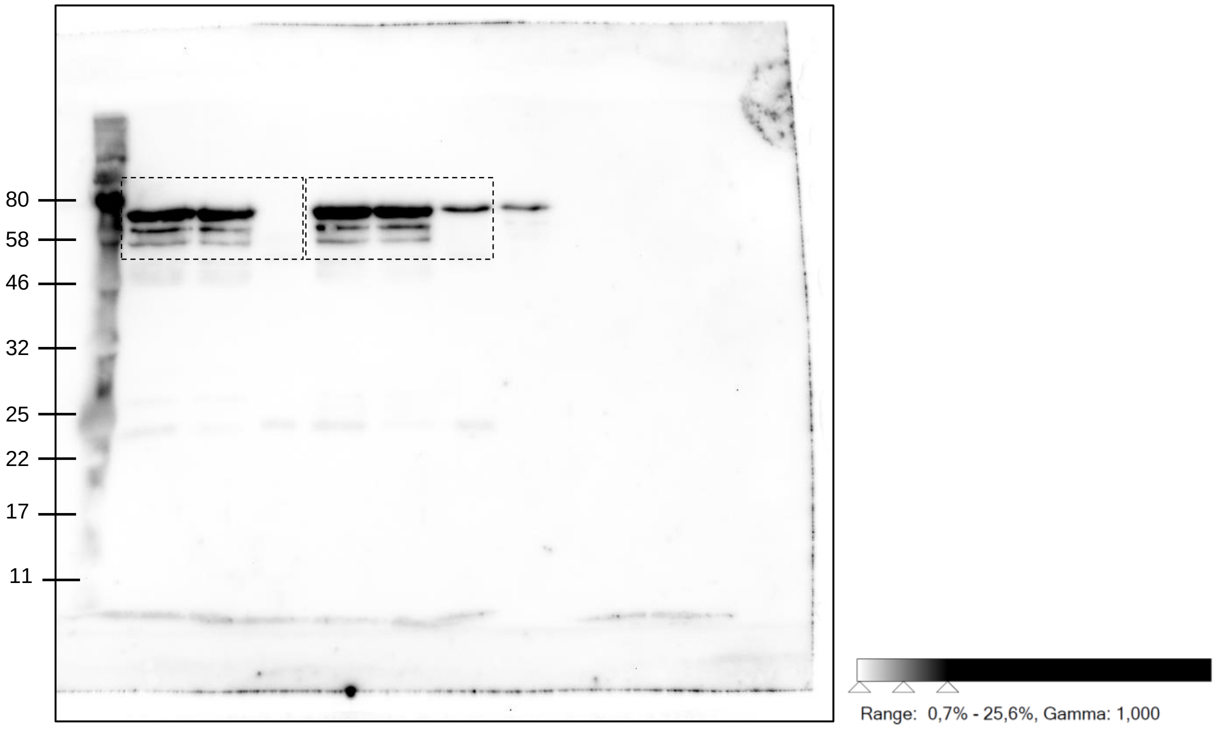

***Figure 4C***

17G6 + $MnCl_2$_HEK293 (left) and _CHO-K1 (right)

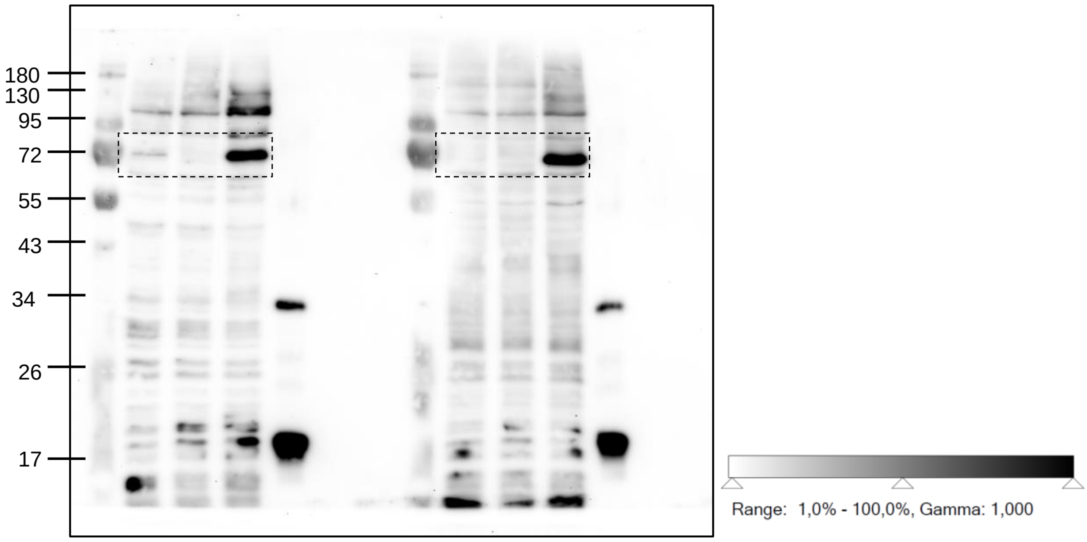


α-BiP_HEK293 (stripped, previously 17G6 + $MnCl_2$)

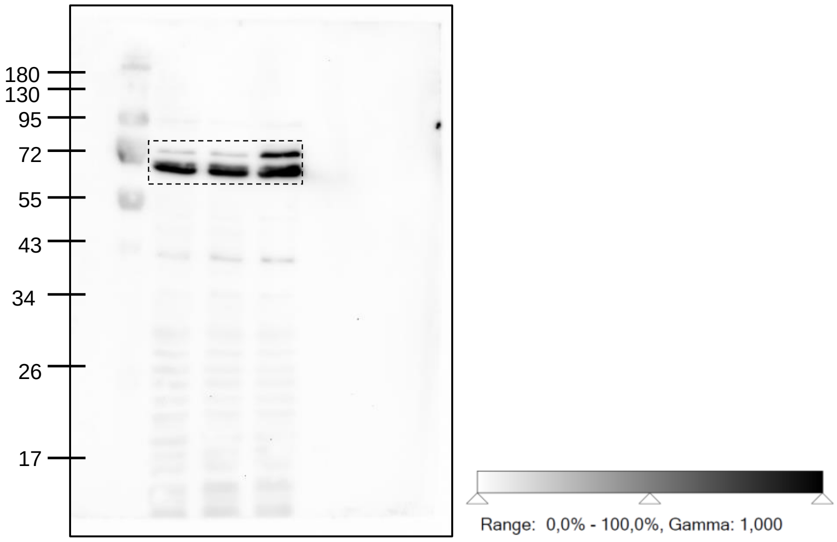


α-BiP_CHO-K1 (stripped, previously 17G6 + $MnCl_2$)

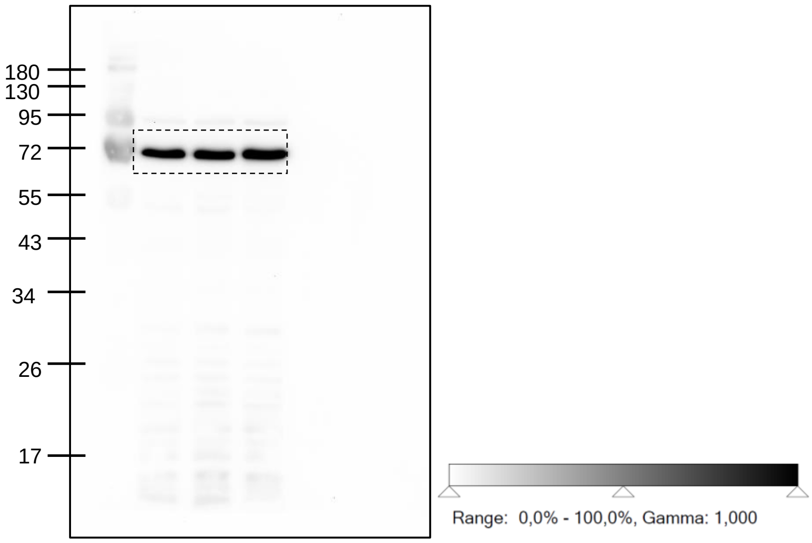

***Figure 4D***

α-BiP_IP:17G6

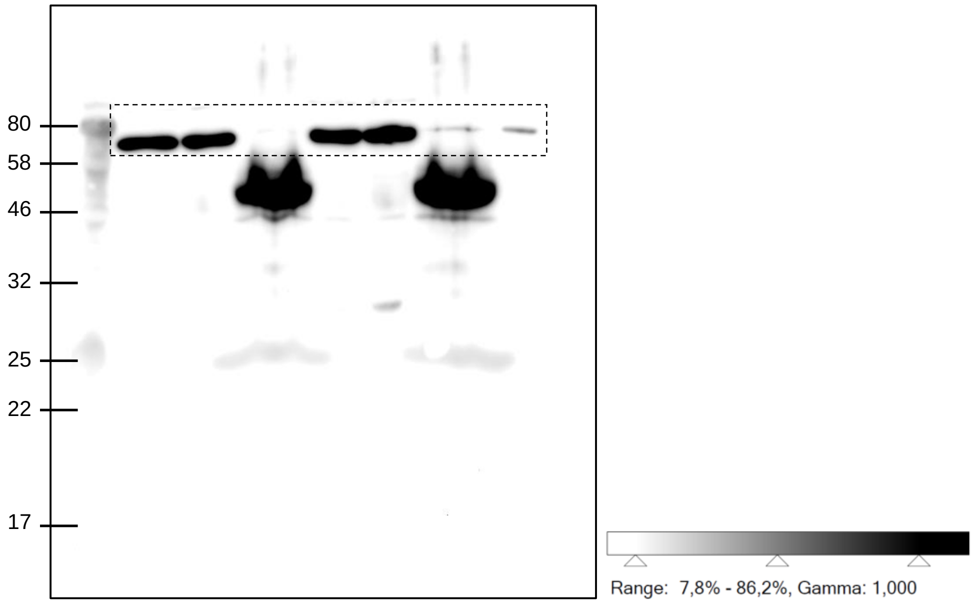


***Figure 4E***

aBiP_IP 17G6_input (left), _unbound (middle), _bound (right)

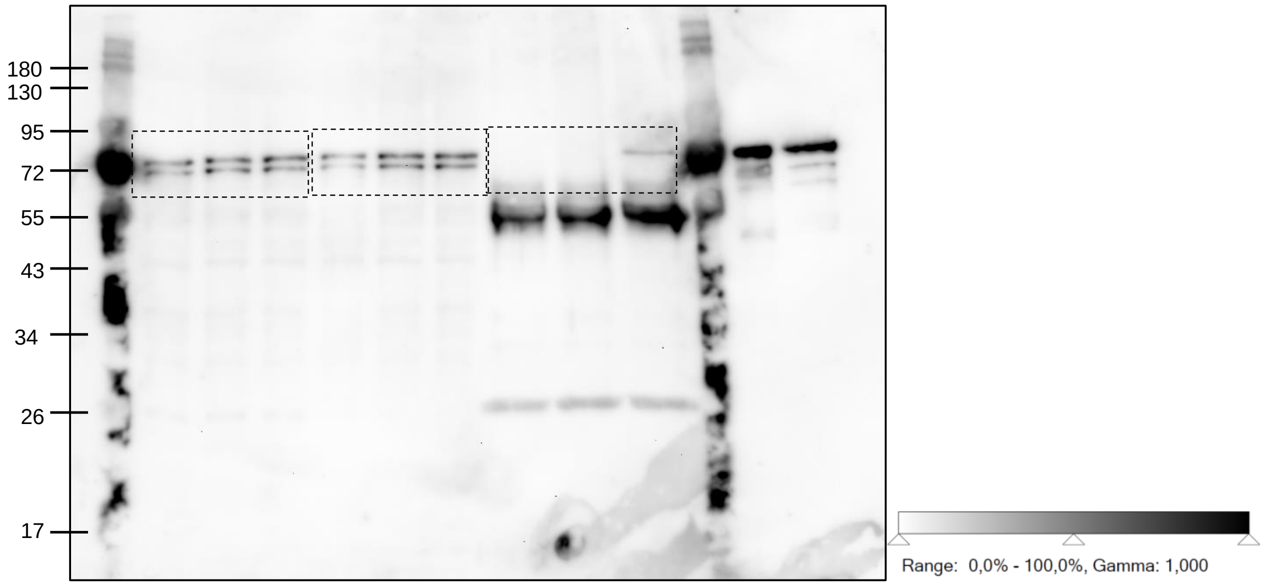

***Figure 4F***

17G6 + $MnCl_2$

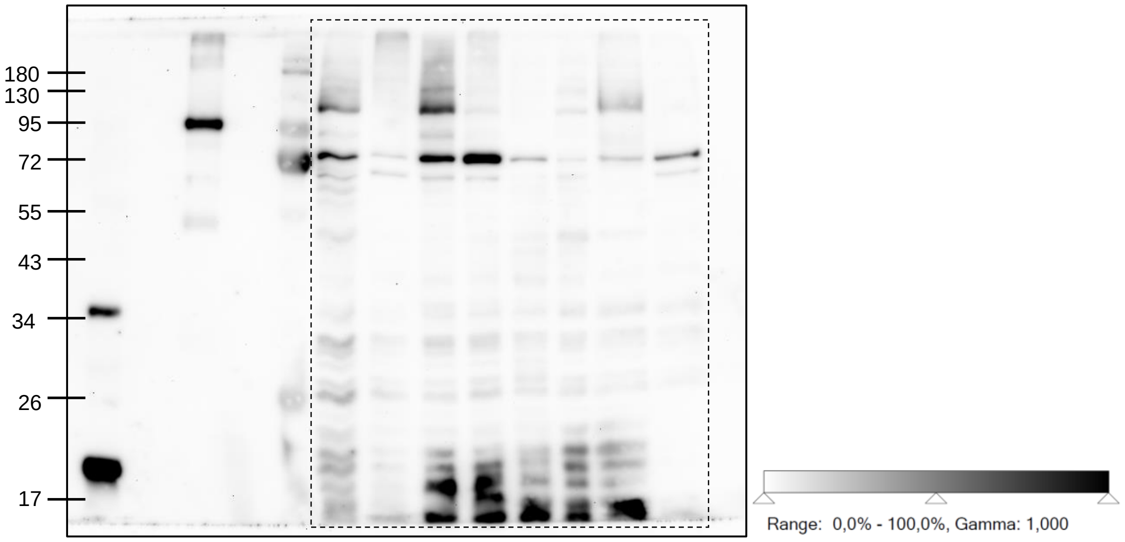


17G6 + $MnCl_2$ (after hydroxylamine treatment)

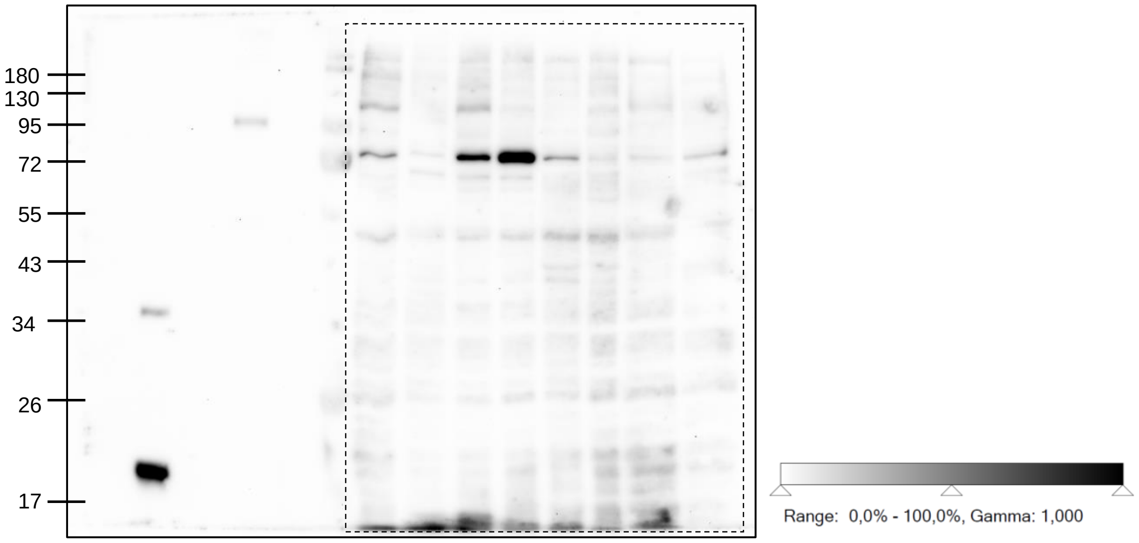


α-BiP (stripped, previously 17G6 + $MnCl_2$)

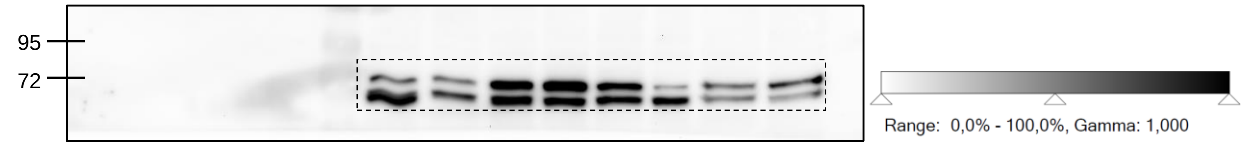


α-GAPDH (stripped, previously 17G6 + $MnCl_2$)

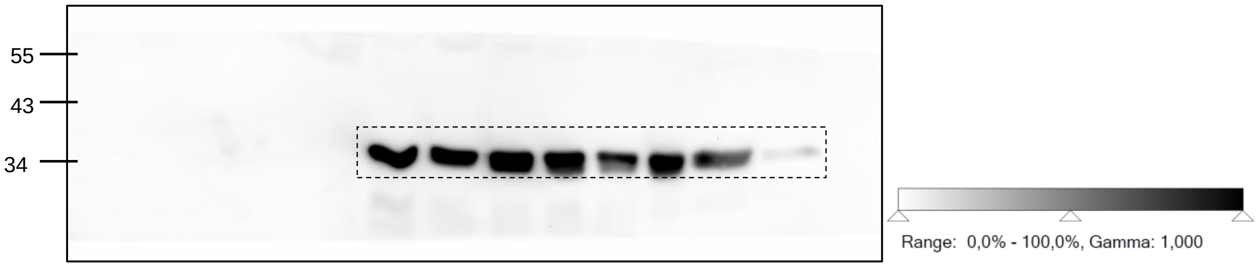

***Figure S4***

1G11 _Cdc42-Thr-AMP (left), _Cdc42-Tyr-AMP (middle), _Rab1-AMP (right)

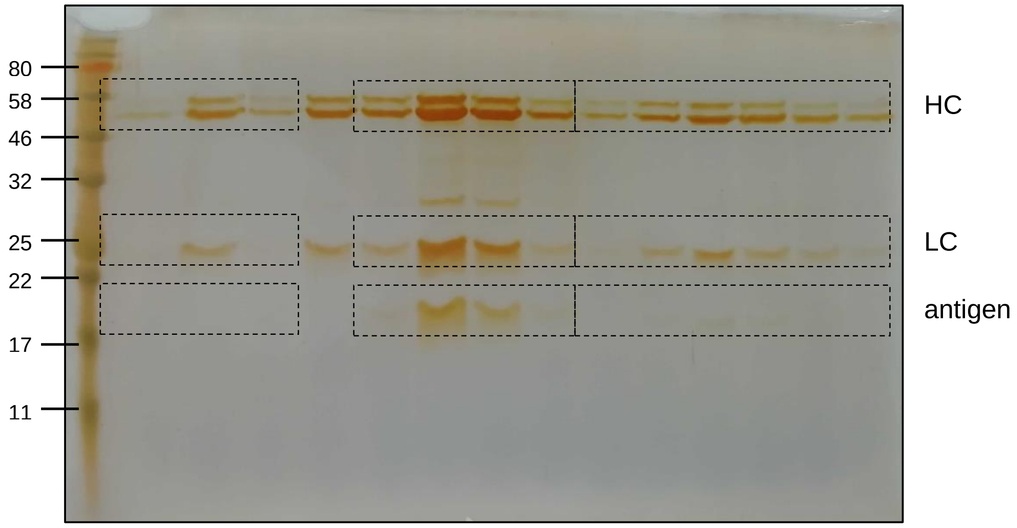


7C11_Cdc42-Thr-AMP (left), _Cdc42-Tyr-AMP (middle), _Rab1-AMP (right)

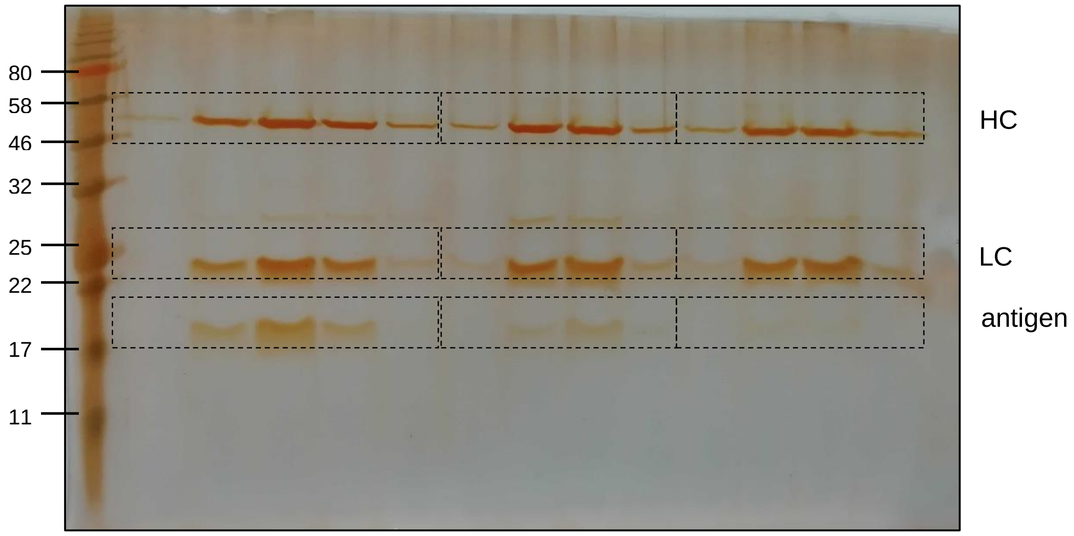

***Figure S6***

17G6 + $MnCl_2$

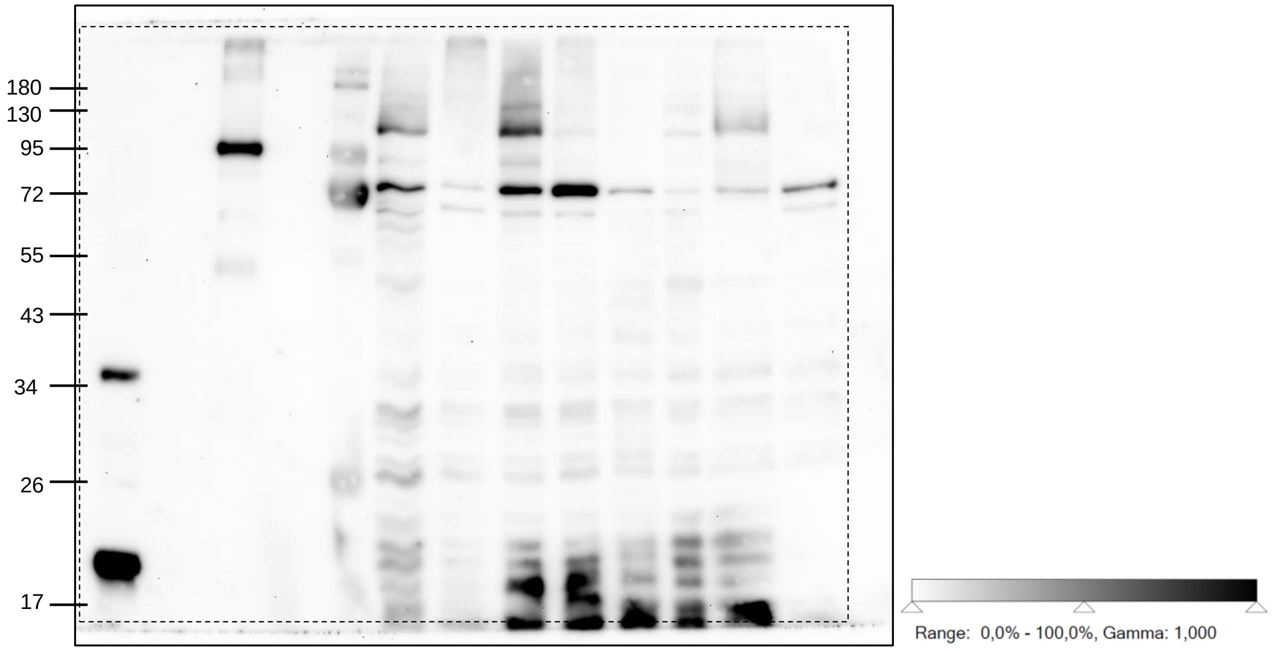


17G6 + $MnCl_2$ (after hydroxylamine treatment)

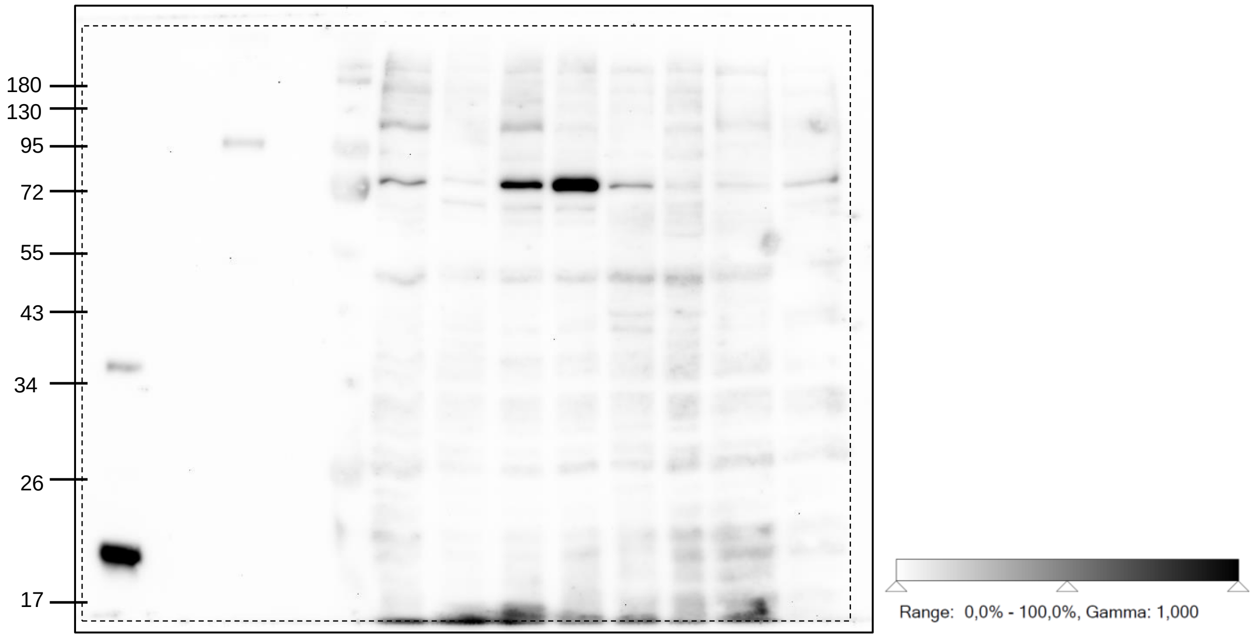


(Same as Figure 4F 17G6 + $MnCl_2$ before and after hydroxylamine treatment)

7C11 + $MnCl_2$

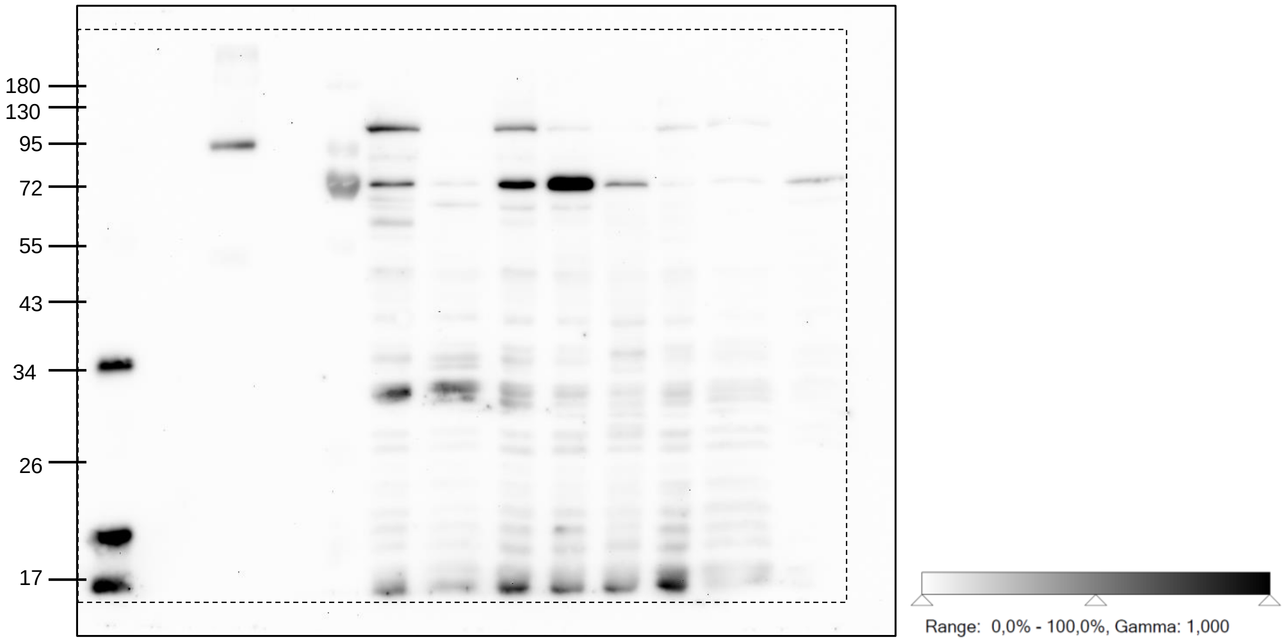


7C11 + $MnCl_2$ (after hydroxylamine treatment)

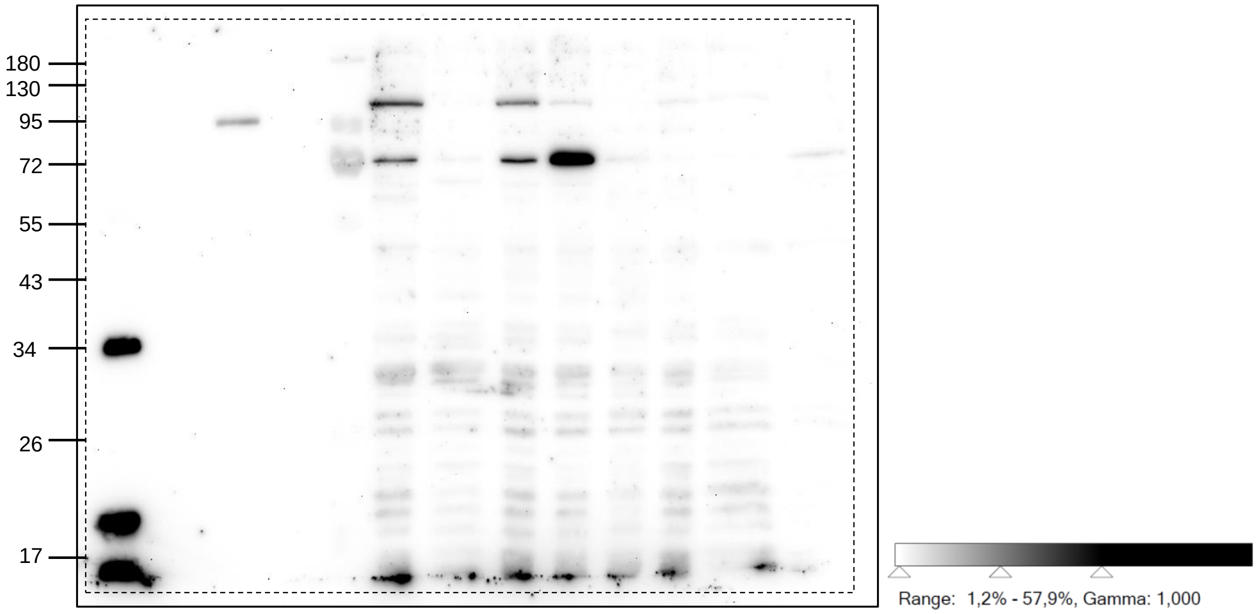

1G11 + $MnCl_2$

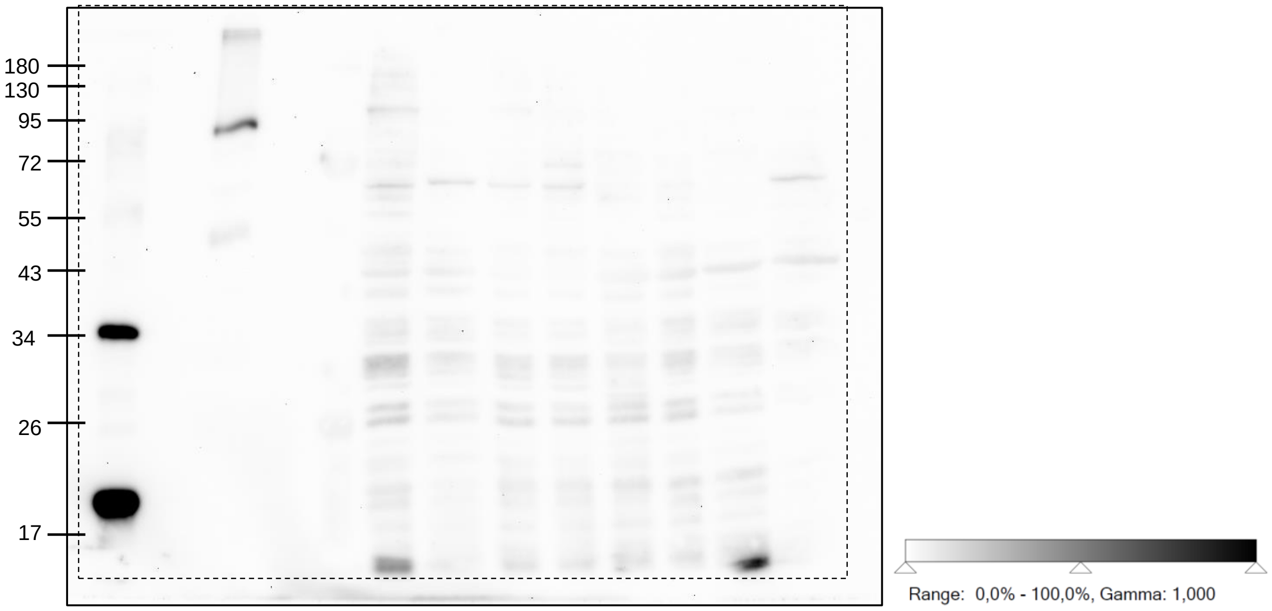


1G11 reprobe without $MnCl_2$

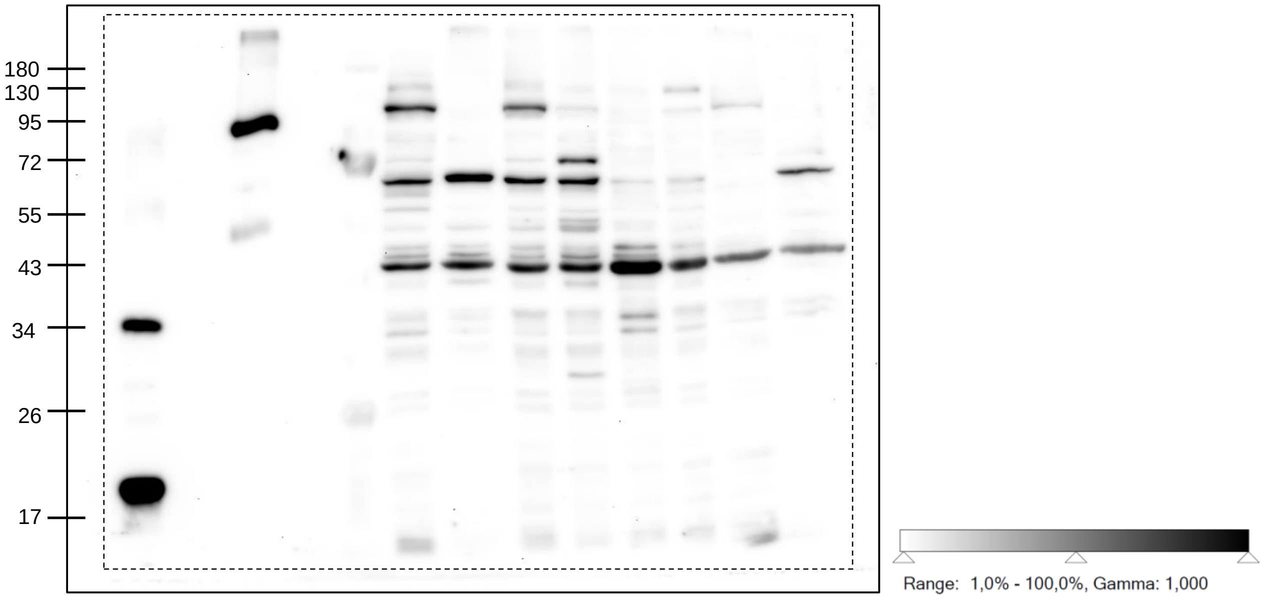

1G11 + $MnCl_2$ (after hydroxylamine treatment)

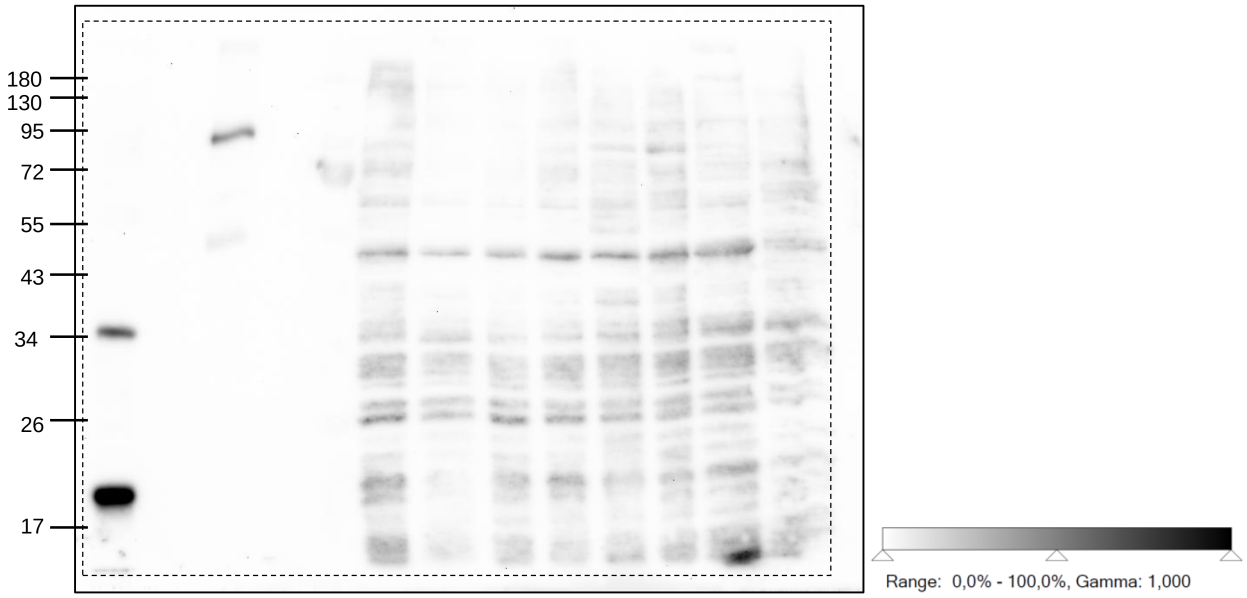


1G11 reprobe without $MnCl_2$ (after hydroxylamine treatment)

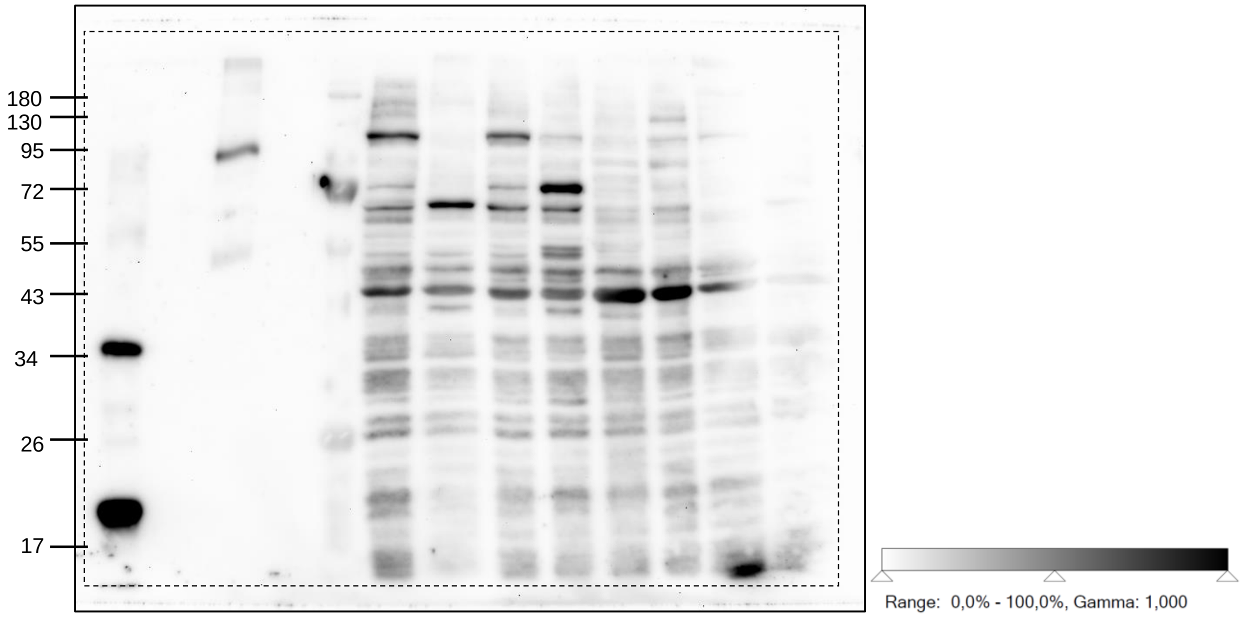

***Figure S7***

a-pan-ADPR (Merck)

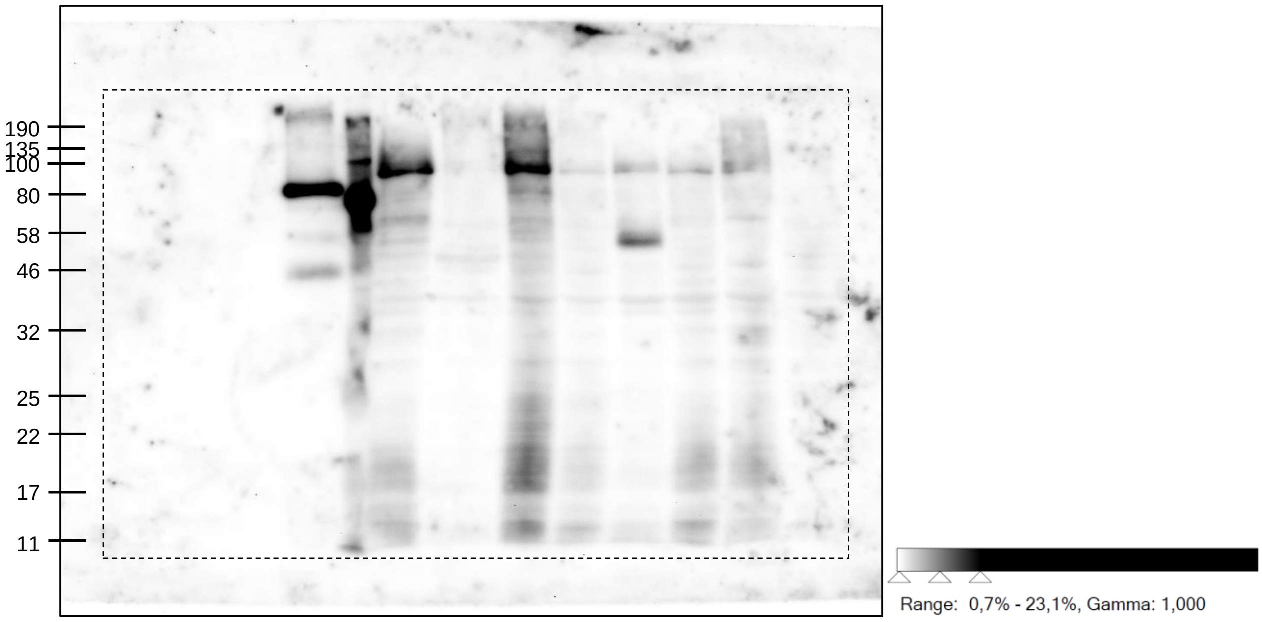


α-pan-ADPR (Merck) (after hydroxylamine treatment)

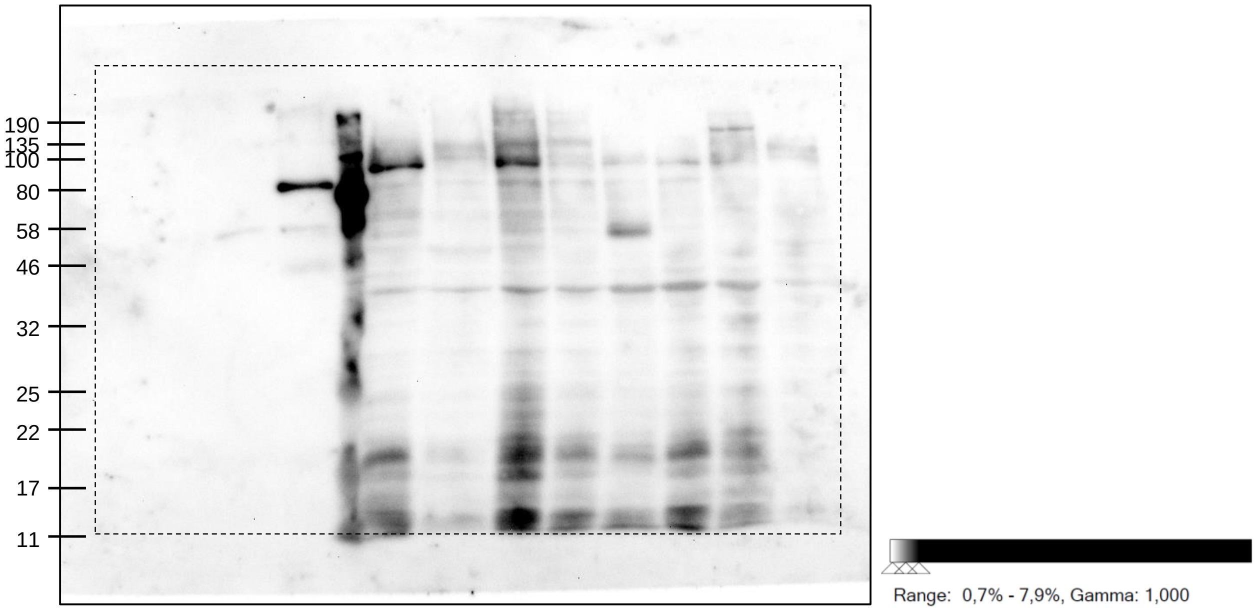

α-Tyr-AMP (Merck)

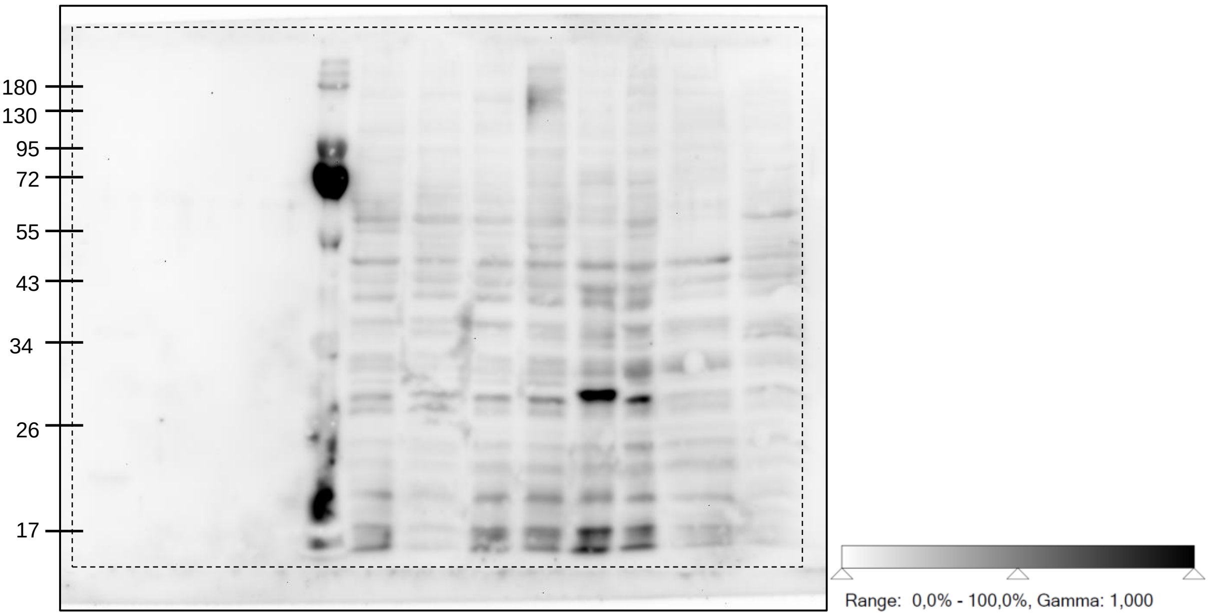


α-Thr-AMP (Merck)

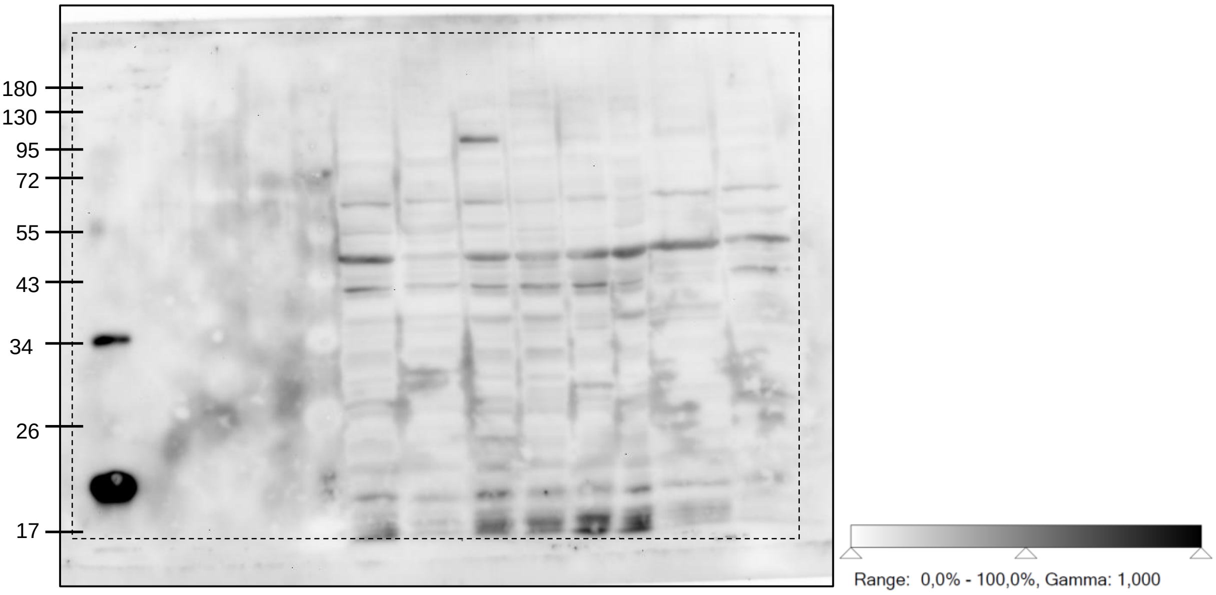


α-BiP (stripped, previously α-Thr-AMP)

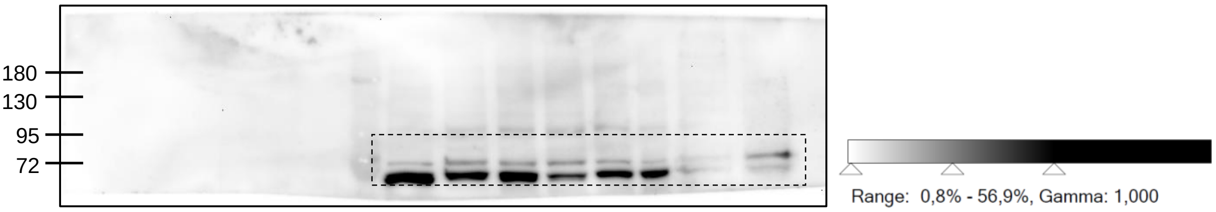


α-GAPDH (stripped, previously α-Thr-AMP)

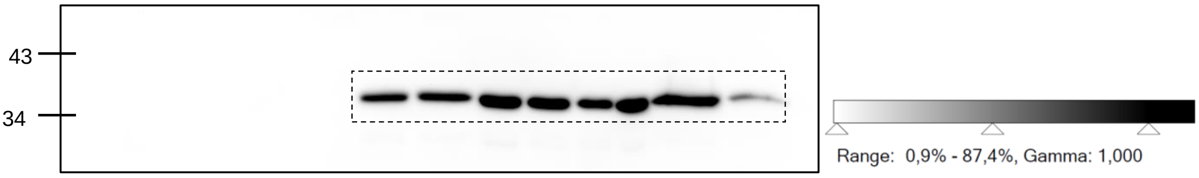

## Supplemental References


Barthelmes, K., Ramcke, E., Kang, H.S., Sattler, M., Itzen, A., 2020. Conformational control of small GTPases by AMPylation. Proc. Natl. Acad. Sci. U. S. A. 117, 5772–5781.

Ernst, S., Ecker, F., Kaspers, M.S., Ochtrop, P., Hedberg, C., Groll, M., Itzen, A., 2020. *Legionella* effector AnkX displaces the switch II region for Rab1b phosphocholination. Sci. Adv. 6, eaaz8041.

Gibson, B.A., Conrad, L.B., Huang, D., Kraus, W.L., 2017. Generation and Characterization of Recombinant Antibody-like ADP-Ribose Binding Proteins. Biochemistry 56, 6305–6316.

Goody, P.R., Heller, K., Oesterlin, L.K., Müller, M.P., Itzen, A., Goody, R.S., 2012. Reversible phosphocholination of Rab proteins by Legionella pneumophila effector proteins. EMBO J. 31, 1774–1784.

Schoebel, S., Oesterlin, L.K., Blankenfeldt, W., Goody, R.S., Itzen, A., 2009. RabGDI Displacement by DrrA from Legionella Is a Consequence of Its Guanine Nucleotide Exchange Activity. Mol. Cell 36, 1060–1072.

Vieweg, S., Mulholland, K., Brauning, B., Kacharia, N., Lai, Y.-C., Toth, R., Singh, P.K., Volpi, I., Sattler, M., Groll, M., Itzen, A., Muqit, M.M.K., 2020. PINK1-dependent phosphorylation of Serine111 within the SF3 motif of Rab GTPases impairs effector interactions and LRRK2 mediated phosphorylation at Threonine72. Biochem. J. https://doi.org/10.1042/bcj20190664

Zhang, Z., Marshall, A.G., 1998. A universal algorithm for fast and automated charge state deconvolution of electrospray mass-to-charge ratio spectra. J. Am. Soc. Mass Spectrom. 9, 225–33.
